# From aspiration to reality: a cross-country analysis of the implementation of lung cancer screening in Europe

**DOI:** 10.3389/fpubh.2026.1766560

**Published:** 2026-04-01

**Authors:** Charlotte Poon, Alejandra Martinez Zamora, Tim Wilsdon, Michael Hartevelt, Arnaud Papin

**Affiliations:** 1 Charles River Associates, London, United Kingdom; 2 Merck & Co Inc, Rahway, NJ, United States

**Keywords:** early detection, implementation, lung cancer, lung cancer screening, screening

## Abstract

**Background:**

Implementation of lung cancer screening (LCS) has been slow across geographical Europe. Only three countries have a formal LCS program: Croatia, Poland, and England.

**Objective:**

This study aims to review the status of LCS and identify the barriers to implementation of formal programs.

**Methods:**

A framework to assess the status and identify key gaps was used, consisting of five dimensions: (1) policy prioritization and governance, (2) clinical review, (3) program design, (4) implementation, and (5) awareness. The research was conducted in 10 countries including Belgium, Croatia, England, France, Germany, Greece, Italy, Poland, Spain, and Sweden. Across countries, the assessment framework was applied to identify key barriers. The assessment was validated with local experts.

**Results:**

Countries are at different stages of implementation, with large variability in the proposed program design. The cross-country analysis found common barriers. A crucial driver of stimulating political commitment is a local network of well-connected clinicians and patients. Although governments request local data to support decision making, in many countries, local data collection is minimal or stalled. Implementation is the most underdeveloped dimension in the framework, commitment to sufficient funding and infrastructure for scaling up pilots and post screening care delivery is a common barrier. Finally, awareness amongst the public and healthcare professionals requires improvement.

**Conclusion:**

Progress depends on a number of factors namely, political will, funding commitment, a local network of well-connected experts, availability of local evidence, and feasibility assessments.

## 1 Introduction

Globally, lung cancer remains the leading cause of cancer-related death. In 2020, it was estimated that lung cancer accounted for 20% of all cancer-related deaths in the European Union (EU) (1). In order to address the high burden of lung cancer, how to achieve earlier diagnosis has been widely debated (2, 3). The momentum for lung cancer screening (LCS) with low-dose computed tomography (LDCT) has grown considerably since the two landmark trials, the National Lung Screening Trial (NLST) and the Dutch-Belgian Lung

Cancer Screening trial (NELSON), which studied their effectiveness on impacting lung cancer outcomes (2).

In 2022, lung cancer screening received positive attention in the EU Beating Cancer plan and European Union Council recommendation aiming to reduce the burden of cancer (4). However, to date only three countries in Europe have recommended and began the implementation of a national LCS program: Croatia and Poland in 2020 and most recently, England in 2023 (5–7). Moreover, in Germany, implementation of a program is expected soon following the adoption of a bill in July 2024 legalizing LCS through LDCT; however, a reimbursement decision is pending (8). Even after the initiation of a formal program, there are still barriers affecting how quickly this is scaled up. For example, in England, capacity, workforce, and funding constraints have been identified as significant barriers for the large-scale implementation of a cohesive LCS program (9).

This study aims to complement our previous research on the design of LCS programs to identify the current status of programs and barriers affecting the steps up to and including implementation (2, 3). We first assess the status of 10 European countries, then consider the barriers that have hindered the implementation of different programs.

## 2 Methods

**Figure 1:**
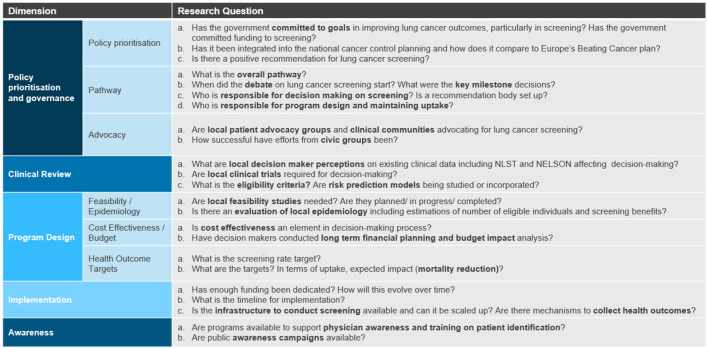
Assessment framework composed of five dimensions.

First, we developed a framework to assess the current status of LCS in the countries. As seen in Figure 1, the framework consists of five dimensions; namely, (1) policy prioritization and governance, (2) clinical review, (3) program design, (4) implementation, and (5) awareness. To develop this framework, we reviewed the existing CFIR framework and augmented this to reflect the key decision-making and adoption factors for LCS identified in our prior publications (10). Our first study reviews the key decision-making factors for lung cancer screening, concluding there are five drivers around issue recognition and political prioritization, clinical evidence, cost effectiveness and budget impact data, local feasibility demonstration and integrated decision-making (2). The second study reviews the factors affecting adoption when lung cancer screening is implemented and we concluded the drivers can be characterized around political, financial and infrastructural issues (3). From these findings, we developed the five dimensions of our research framework:

- The policy prioritization and governance dimension focuses on the level of government commitment to lung cancer as evidenced by inclusion of lung cancer into the National Cancer Plan (NCP) and committed funding. We also assess whether there is a clear pathway from evaluation to implementation with clearly defined responsibilities. Moreover, we evaluate the advocacy efforts of patient and medical communities to move forward the LCS debate.
- The clinical review dimension is evaluated based on the necessary data package requirements for decision-making, the perception of the clinical data currently available for LCS, particularly the two landmark studies NELSON and NLST, and the emerging eligibility criteria from ongoing pilots or government recommendations.
- The program design dimension is assessed based on how LCS programs are curated, including whether there is a need for feasibility and epidemiology studies and the review of cost-effectiveness and budget impact. This dimension also assesses the screening and health outcome targets.
- The implementation dimension assesses the readiness and availability of the necessary resources for the implementation of a LCS program including sufficient funding commitment, development of an implementation timeline, and adequate infrastructure to support screening and downstream services.
- Finally, the last dimension of the framework evaluates the development of awareness campaigns and trainings to combat stigma toward lung cancer.

In the second step, we selected countries across Europe. The countries were selected to represent those in different stages toward a program namely, countries with (1) an implemented LCS program, (2) an ongoing pilot and (3) in exploratory stages of a pilot. The countries within each category were selected if there was evidence of recent movement in the debate for LCS in the country e.g., announcement of a national program, or an evaluation by the Health Technology Assessment (HTA) body. We selected: Belgium, Croatia, England, France, Germany, Greece, Italy, Poland, Spain, and Sweden. The country selection was also made to ensure there is representation across different regions in Europe and where there is a relatively mature political system for transparent decision making with readily available information. We gathered information on the application of the current screening programs or pilots in terms of eligibility criteria, screening frequency and use of detection software.

In the third step, we undertook a literature search to gather information across the dimensions of the framework. This included a review of academic articles through a PubMed search, government official reports, non-governmental organization (NGO) publications and media reports up until October 2024. A keyword search was used to identify relevant literature. The search used combinations of terms such as: “LCS program,” “implementation,” “pilot studies,” “positive recommendation,” “key enablers” and “barriers.” We excluded studies that were out of date (earlier than 2015) or entirely theoretical or based on anecdotal evidence. The literature search was conducted in both English and local language and resulted in 128 documents included in the analysis. The framework was then applied to identify the critical gaps in the LCS programs across countries. We qualitatively assessed the level of action required for a successful LCS program for each dimension of the framework. Each area was qualitatively scored as either low if minimal action was required, medium if there were some areas which require action for a successful program, or high if there were critical gaps which required immediate action for a successful program.

Fourth, we consulted with local stakeholders representing perspectives from policy, academics, and patient representative angles to review and validate our assessment. This included one-on-one virtual meetings with twelve experts, one for each of the countries analyzed with the exception of the UK and Greece were we met with 2 experts and Poland which had no expert (Dr. David Baldwin,^1^ Mr. Ivica Belina,^2^ Dr. Richard Booton,^3^ Dr. Sebastien Couraud,^4^ Ms. Ebba Hallersjö,^5^ Dr. Nikos Koufos,^6^ Dr. Sofia Lampaki,^7^ Dr. Eugenio Paci,^8^ Mr. Sebastien Schmidt,^9^ Dr. Annemiek Snoeckx,^10^ Dr. Juan Carlos Trujillo,^11^ Ms. Eleanor Wheeler^12^). The experts reviewed draft country profiles, validated our country assessment, and identified top barriers that need to be addressed. We conducted semi-structured interviews with each expert to discuss our research methodology, the findings from the literature review including our barriers to implementation, our qualitative scoring on level of action required and our conclusions. The feedback from the experts were incorporated into the presented research findings.

## 3 Results

### 3.1 Overview of the LCS program landscape across Europe

**Figure 2:**
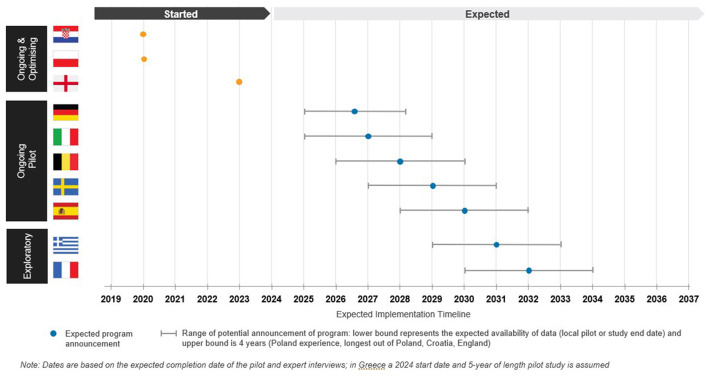
Expected directional timeline for implementing LCS. The orange dot represents the start date of the formal LCS programs. The blue dot represents the expect date of a LCS program announcement. The lower bound of the range is the date at which pilots are expected to end based on the publicly available data. Based on the average time from pilot / local study end to program announcement in Poland, England, and Croatia (NELSON trial readout used for Croatia), around 2 years is required from the completion of pilot to a positive recommendation to be announced. The upper bound of the range is based on the country that took the longest from pilot completion to a positive decision 4 years (Poland). For Germany, given the BMUV's bill and announced target dates, the range of uncertainty is smaller. The proposed timelines are based on trends from Poland, England and Croatia which substantially differ in political contexts and health system capacities^a^ (see text footnotes 14, 19),^b^ (19, 55–59). Dates are based on the expected completion date on the pilot and expert interviews; in Greece a 2024 start date and 5-year of length pilot study is assumed. ^a^NACIONALNI PROGRAM PREVENCIJE RAKA PLUCA.Pdf,”, https://zdravlje.gov.hr/UserDocsImages/2019%20Programi%20i%20projekti/ NACIONALNI%20PROGRAM%20PREVENCIJE%20RAKA%20PLU%C4%86A.pdf. ^b^RISP - Progetto Della Rete Italiana Screening-Polmonare.Pdf.

Based on the current status of LCS, we can segment the 10 countries reviewed in this study into three categories (see Figure 2 for expected timeline of implementation).

First, there is a category of “ongoing and optimizing” countries defined as those that have established a formal LCS program which is publicly funded and are actively screening the eligible population i.e. Croatia, England, and Poland. Croatia and Poland began their national LCS program in 2020. As of 2023, 22,000 people have been screened in Croatia and 16,946 LDCT scans have been performed in Poland (11).^13^ In July 2023, England announced the roll-out of a national program and as of September 2024, a total of 589,292 scans have been performed and 5,271 lung cancers have been diagnosed.^14^,^15^

Second, there is a category of “ongoing pilot” defined as countries with ongoing studies such as Belgium, Germany, Italy, Spain, and Sweden driven by the need for local feasibility and cost-effectiveness data for decision making. Germany has legalized LCS through a Federal Ministry for Environment, Nature, Conservation and Nuclear Safety (BMUV) ordinance on July 1, 2024. A Federal Joint Committee (G-BA) decision on implementation and reimbursement is now required and is expected as early as the start of 2025 (8, 12). In Italy and Spain, national pilots which were organized by the academic community started in 2021 and 2023, respectively (13).^16^,^17^ Regional pilots are ongoing in Sweden and Belgium with results expected in 2026 and 2027, respectively (14, 15).

The last category we define as “exploratory” countries who are in early planning stages for a local pilot, such as France and Greece. In 2022, the National Health Authority (HAS) reviewed LCS and recommended the French National Cancer Institute (INCa) to conduct a national pilot, anticipated for 2025–2030 (16, 17). In Greece, the Ministry of Health (MoH) announced a pilot across four hospitals in 2023; however, launch of these pilots has been delayed and there is uncertainty on a start date (18).^18^

### 3.2 Program design characteristics

In addition to the status of the screening program varying significantly across the countries included, there is significant heterogeneity across countries when comparing the program design.

**Figure 3:**
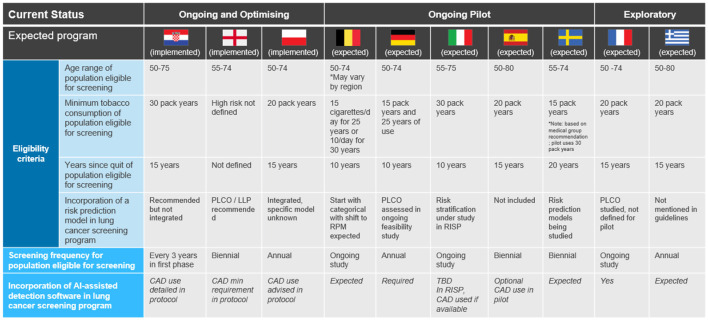
Overview of lung cancer screening program design (see text footnotes 14, 19, 20, 21, 23) (19, 55, 56, 59).^a^ In countries without an official recommendation, the eligibility criteria and frequency is defined from the pilot study methodology or medical group recommendation. ^a^Greece lung cancer screening medical societies recommendations - ΠPOTEINOMENH ΔIAΔIKAΣYM ΠPOΣYMΠTΩMATIKOY EΛEΓXOY KAPKINOY ΠNEYMONA (ΠEKΠ).

Figure 3 provides an overview of the eligibility criteria (the population deemed as high risk for lung cancer and that will be invited to be screened), screening frequency (how often population will be invited to be screened) and the use of detection software across the countries. For countries where a national program is not yet in place we include information based on the protocols established for the pilot programs giving some indication on the outlook. Although this could change, we have observed from the countries where a national program now exists, the program design followed closely to the pilot. There is one exception where the national protocol in Poland was updated based on literature from the international medical community.^19^

There is significant variation in the eligibility criteria for screening. The eligible age range varies with the widest range of 50–80 years in Spain and the narrowest, of 55–74 years in England and Sweden (19) (see text footnote 14).^20^ Likewise, there is no consistency in the minimum tobacco consumption required with eligibility criteria ranging from 15 to 30 pack years, nor the “years since quit” required with eligibility criteria ranging from 10 years to 20 years. Moreover, only England is currently using a risk prediction model (a mathematical equation that takes data on patient risks factor to estimate the probability of experiencing a healthcare outcome) (20). The two specific models are LLP Lung Cancer Risk model which estimates whether an individual will develop lung cancer within a 5 year period and PLCO_m2012_, based on a 6 year cancer incidence (21, 22). While Croatia and Poland started their national program with the simpler categorical risk approach, Poland later integrated a risk prediction model and Croatia plans to do so in the future (23).^21^,^22^

Screening frequency also varies significantly. The frequency differs between annual and biennial screening except for Croatia, who recommends a scan every 3 years for the first phase of implementation (2020–2025). Croatia is the only country to introduce a national program without a local pilot and the protocol suggests revisions can be made as more local data is collected (see text footnote 25).

All countries agreed that Computer Aided Detection software using AI should be incorporated to manage the workload of radiologists and improve accuracy and rates of missed lung nodules.

### 3.3 Status of LCS: barrier assessment

**Figure 4:**
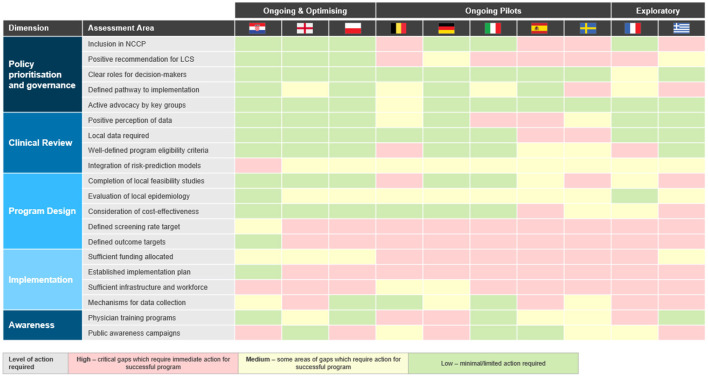
Overview of critical gaps across countries using the framework assessment.

The assessment framework has revealed where further action is required. Figure 4 provides an overview of the areas where minimal, medium or high level of action is needed. The section below summarizes the critical gaps between countries across the five dimensions as well as highlights facilitators identified from countries where minimal action is required. A detailed profile for each country can be found in Supplementary material A and B.

#### 3.3.1 Policy prioritization and governance

##### 3.3.1.1 Policy prioritization

There has been strong political commitment to improving lung cancer outcomes in the “ongoing and optimizing” countries (24, 25). In Croatia and England, lung cancer outcome targets were incorporated into national health plans which elevated screening as an important part of lung care and established a goalpost.^23^,^24^ Implementation of LCS in early adopter countries such as Croatia and Poland is directly related to political prioritization with screening employed as a tool to address the pressing need to improve lung cancer mortality (see text footnote 25).

Governments in France and Italy are more vocal about considering LCS, with commitments to dedicate funding to assess the implementation of LCS as a priority area in their national cancer plans (26, 27). However, in six of the seven “ongoing pilot” and “exploratory” countries, generating political momentum remains a critical gap. Belgium, Greece, Spain, and Sweden have not outlined commitments in LCS in their NCPs (28–31). However, updates to NCPs are expected in Greece and Sweden in 2025 and November 2024, respectively, which may advance the debate (32, 33).

##### 3.3.1.2 Pathway for decision-making

Generally, the responsibility of the decision makers in the evaluation process is clearly defined across all countries reviewed. In countries where cancer screening is regulated at a regional level such as in Belgium, Spain and Sweden, decentralization is a significant challenge leading to disparate implementation (34–36). For example, in Belgium, each region is responsible for organizing and implementing screening programs (34). This decentralization has led to varying implementation timelines across regions for other screening programs such as colorectal cancer screening which was introduced in Brussels in 2002 and not in Flanders until 2013 (37). As of 2024, only Flanders has been active in the debate for LCS.^25^

##### 3.3.1.3 Advocacy

Across all countries, a local patient advocacy group was active in the local debate with no critical gaps uncovered across countries. Given lower survivorship, experts have remarked that the patient advocacy community is less developed compared to other cancers. Where countries have been more successful, patient groups were involved in the decision-making process. For example, the patient group in England has participated in the National Screening Committee, the key committee for decision making (38). There are also examples of patient advocacy groups directly influencing the design or funding of pilot programs such as CASSANDRA in Spain (19, 39).

Moreover, in majority of the countries, the clinical community have an active role in shaping the local debate. For example, in Poland, a consensus paper between clinical experts was published for recommendations on implementation of LCS (see text footnote 24). Similarly, the local thoracic society and radiology group in Croatia was influential in designing the national guidelines (see text footnote 25). In Belgium, the Flemish Lung Cancer Task Force formed by medical societies is leading the local pilot and generating the political momentum for the implementation of a program in the Flemish region. On the other hand, other regions in Belgium lack a similar taskforce and have yet to plan any local pilots.

#### 3.3.2 Clinical review

Six of the 10 countries analyzed have critical gaps relating to the clinical review. Most countries in this study accepted the landmark studies, NELSON and NLST and reviewed the evidence along with several other randomized clinical trials. However, no formal evaluation of the NELSON or NLST study has been published in Italy (40). Critiques of the studies cited heterogeneity across randomized controlled trials (RCTs) and inability to translate the outcomes into a local context as concerns. Therefore, all country review bodies, with the exception of Croatia, have requested and/or reviewed local data. For example, in Belgium, along with a positive Health Care Knowledge Center (KCE) report, regional health authorities will require regional implementation studies for the adoption of LCS (see text footnote 10). In Sweden, the National Board of Health and Welfare have recommended feasibility studies in order to evaluate the implementation of screening in the local context. As of 2024, there is one ongoing feasibility study organized by the Regional Cancer Center Stockholm-Gotland. (41).

There are also lessons for establishing the appropriate model for eligibility. While governments recognize the need for risk prediction models, limited progress has been made across countries in identifying the appropriate model for the local context. The HANSE feasibility study in Germany which compared the NELSON and PLCO_m2012_ risk scores is a good example of how to evaluate which model would be optimal for implementation (42, 43). The study concluded the use of PLCO_m2012_ was more reliable and efficient in Germany (43).

#### 3.3.3 Program design

##### 3.3.3.1 Feasibility studies

Looking at the countries that are running feasibility studies, the pilot programs in Spain and Italy are operational, but the progress in the remaining countries is minimal or even stalled with critical gaps identified for three countries. In Sweden, there is currently only one pilot project underway in one region, organized by the Regional Cancer Center (RCC) Stockholm-Gotland but several other are being planned by RCC Syd, RCC Norr and RCC Vast.^26^ Similarly in Belgium, only one region, Flanders, has a pilot study which began this year.^27^ In Greece, pilot programs have been already announced by the MoH and were expected to start in 2023.^28^ However, it has been recently announced the pilot program would now begin in 2025 (44). While in France, there has been a number of local studies, the first national pilot conducted by INCa was only recently established (45).

##### 3.3.3.2 Cost-effectiveness

Cost effectiveness is not consistently reviewed across countries but where it is, local cost-effectiveness studies were needed. Critical gaps in Spain and Greece surrounding local data were identified. In Spain, the HTA body, RedETS, conducted a cost-effectiveness analysis and cited a lack of cost-effectiveness as one of the reasons leading to a negative recommendation in 2023.^29^ In order to address this concern, the CASSANDRA pilot will assess cost-effectiveness with leaders expecting similar results to the cost-effectiveness of other screening programs already implemented in Spain (see text footnote 29). In Greece, it will be important to gather cost-effectiveness data through pilots in order to persuade decision-makers and justify the investment in LCS (see text footnote 18).

##### 3.3.3.3 Heath outcome and screening targets

Setting health outcome targets remains a critical gap in nine out of the 10 countries analyzed. Both England and Poland have set out targets for their ongoing LCS programs, focusing on achieving a set number of screens per year as a scalability target rather than setting targets for improving lung cancer outcomes. For example, in England, the government announced estimates of as many as 9,000 persons per year detected with cancer through the LCS program (see text footnote 14). However, national goals for the program in respect to 5-year survival and screening rates have yet to established. In Poland, the national protocol established four overarching goals including increasing the number of lung cancer detected at earlier stages in the range of 42%−65% and outlined key areas that should be evaluated (e.g., assessment of the change in mortality rate and survival rate; see text footnote 24). However, no specific targets were set for these evaluation areas outlined in the protocol. It is unclear how the national program in Croatia has performed. Despite the Croatian national protocol and NCP defining long-term goals such reduction of lung cancer mortality by 20% in the next 5–10 years, there are no publicly available reports on the performance of the program since it began in 2019 (46) (see text footnote 19).

As Germany is at the first phase of roll out for a national program, it is currently unclear whether the G-BA will include screening and health outcome targets in the implementation guidelines that are currently in development. In addition to setting national targets, continued monitoring of the performance of a LCS program is needed.

As expected, countries which fall into the “ongoing pilot” and “exploratory” category have no health outcome or screening targets yet, given their stage of implementation.

#### 3.3.4 Implementation

##### 3.3.4.1 Funding

Across all countries that have implemented LCS, it is unclear whether sufficient funding has been committed for the continuity of the program. For example, in Poland, the first phase of implementation was co-financed by the MoH and the European Social Fund under a 3-year POWER grant.^30^ As described in the NCP, public funding is expected from 2024; however, it is uncertain whether sufficient funding has been secured for the continued expansion of the program.^31^ Similarly, in England the existing Lung Health Checks are expected to run until 2028 until formal rollout of the national program.^32^

Expert interviews have also highlighted concerns with the funding of downstream services. While the programs in England, Poland and Croatia are currently funded, there are concerns with the uptick in lung cancer care services as dedicated funding allocations for these areas have not been formalized.

##### 3.3.4.2 Infrastructure

Capacity for scaling up screening in the current programs in Croatia, England, and Poland is an issue. For example, in England, workforce and capacity constraints affecting the NHS pose the greatest challenge with a concern over the current imaging workforce shortage and expectation of greater attrition of radiologists in the next 5 years (9, 47). England has 8.8 CT scanners per million which is lower than the OECD average of 29.5 scanners.^33^,^34^ Moreover, government spending freezes create financial challenges for purchasing and upgrading the necessary equipment, including CTs, for the program (48) (see text footnote 3). Likewise, in Croatia and Poland, there is uncertainty in whether the current infrastructure is able to sustain the expansion of the LCS program including the surge in demand of downstream services (see text footnote 24).

#### 3.3.5 Awareness

##### 3.3.5.1 Physician awareness

Discussions with experts have revealed that training on LCS and accreditation for medical professionals is still naïve, compared to other cancers, and remains a critical gap in three countries. In addition, despite advocacy by medical societies for LCS, experts highlighted the need for greater awareness among general practitioners (GPs) in order to increase participation rates. There are countries which have ensured physician awareness is considered in the program organization, for example, in Croatia, GPs and community nurses are at the center of the national strategy with financial incentives in place to drive participation in outreach to the eligible population (49) (see text footnote 25). In Poland, the medical community developed training for radiologists on quality assurance and guidance for general practitioners (GPs) on the evidence and inclusion criteria for the screening program to encourage adoption (50). Moreover, in Italy, one of the objectives of the RISP pilot program is to develop guidelines and training programs for screening (51).

##### 3.3.5.2 Public awareness

The level of public awareness on LCS is low and remains a critical gap in four of the countries reviewed, despite broad acknowledgment from governments that this is important for adoption of screening. For example, the 2020 Smoking Survey by the Belgian Foundation Against Cancer found that only 54% of respondents were aware of the screening initiative (52). England, Italy, and Spain have made more progress in public awareness where targeted public campaigns for the local pilots were identified (53, 54).^35^ However, in Croatia, France, Germany, Poland, and Sweden public awareness campaigns were limited. For example, for the “Spyros Doxiadis” program in Greece, no campaigns specific to the first four pilots were identified, with limited public information available on the pilot initiation.

## 4 Discussion

The cross-country analysis reveals that generating political will for lung cancer and screening is critical for the implementation of a program. As is expected, obtaining a positive recommendation from the MoH and/or the HTA body, is a key step toward implementation. In all countries, excluding Croatia, review bodies have cited the need for local evidence to support a positive recommendation. However, in countries with limited political willingness to support and finance the collection of local evidence, countries reach an impasse. For example, in Spain, despite leaders of the national pilot, CASSANDRA, pressing the national government for financial support, there has been minimal government support for the pilot.

As seen in “ongoing and optimizing countries,” increased political will can be achieved by establishing national goals for lung cancer in NCCPs and positioning screening as a tool to decrease lung cancer burden. Moreover, establishing a national goal post could be impactful for stimulating homogeneity, knowledge sharing and collaboration across regions. Likewise, a local network of well-connected medical and patient champions is crucial for raising political will; however, the next step is for them to play a more active role in the decision-making process as an advisor in the screening committee or supporting the design and operationalisation of the pilots. At the basic level, this group are an invaluable partner in raising awareness, improving public awareness of lung cancer symptoms and the benefits and risks to LCS as seen in Croatia and Poland.

Across all dimensions, implementation is the most underdeveloped. Understandably, it is a downstream priority for many of the countries in scope of this study; however, it is and should be a pressing issue for “ongoing and optimizing” countries where screening is already available. The existing programs in Croatia, England, and Poland have not yet achieved peak screening and there are concerns on scalability and sufficiency of current resources to achieve this. Therefore, greater attention is required in the planning stage to understand how countries will scale up the pilot to a national program and dedicate long-term funding channels toward screening. Similarly, attention is needed on the downstream events following a positive screen if LCS is to be provided sustainably. Countries need to be prepared to relieve bottlenecks in the patient journey due to the uptick in cancer care services.

For a LCS program to be successful, increased awareness across GPs and the public is needed. Dispersion of expertise is important as the knowledge and awareness around LCS is still concentrated in respiratory or radiology communities which ultimately, translates into downstream challenges in accurately identifying target populations in the primary care setting when the program is running. Therefore, incorporation of physician trainings and awareness campaigns among GPs are key to encourage adoption of a screening program. Low public awareness of LCS remains a barrier to uptake. Thus, public awareness campaigns tackling the stigma behind lung cancer and informing of the benefits of LCS will be needed to meet screening targets.

Our study is not without limitations. Our set of factors are compiled from a review of the data available in public domains using key search terms and not a systematic literature review using established protocols. We recognize that there are other factors which are more complex to account for such as the underlying policy environment for health in the countries. Moreover, we review the factors in isolation but the implementation of LCS in countries is likely to be a dynamic process with interaction between multiple factors. In this analysis, we assume that the chain is only as strong as the weakest link i.e. the implementation of LCS is most limited by the largest gap in the system. Our study provides a qualitative assessment of LCS facilitators and barriers; however, future studies could integrate further quantitative assessments and analysis. Furthermore, we provide an overview of the screening program designs across countries with future studies needed to further evaluate the implications of the variation across programs on effectiveness and equity which can further inform program design.

## 5 Conclusion

Despite positive attention from the European Beating Cancer plan, the implementation of lung cancer programs by European governments has been slow. There are some notable exceptions from which we can draw policy lessons. These show that progress implementing LCS can be made, but it depends on several factors and availability of the following:

1. Political will is the first and most important priority to elevate lung cancer as a national priority and can be stimulated by setting national goals or targets for lung cancer outcomes
2. Funding commitment in assessing feasibility of LCS to create certainty in the completion of local pilot studies, where required for a positive clinical assessment. As in Croatia, international clinical data may be enough; however, undertaking local feasibility studies may help mitigate and plan for downstream implementation barriers
3. A local network of well-connected experts of patient and clinical societies to champion lung cancer as a priority
4. Evidence to inform good program design, to ensure the appropriate application of screening and successful adoption
5. Assessment on scalability for implementation planning in terms of delivering screening but also for the downstream events given the expected uptick in lung care services.

## Footnotes

1 “Expert Interview with Dr. David Baldwin (England, Chair of the UK Clinical Expert Group for Lung Cancer),” Personal communication, Moderated by CRA.

2 “Expert Interview with Mr. Ivica Belina (Croatia, President of the Coalition of Associations in Healthcare),” Personal communication, Moderated by CRA.

3 “Expert Interview with Dr. Richard Booton (England, Director of Manchester Lung Health Check),” Personal communication, Moderated by CRA.

4 “Expert Interview with Dr. Sebastien Couraud (France, Head of the Lyon-Sud Pulmonology Department),” Personal communication, Moderated by CRA.

5 “Expert Interview with Ms. Ebba Hallersjö (Sweden, Co-Founder of Vision Zero),” Personal communication, Moderated by CRA.

6 “Expert Interview with Dr. Nikos Koufos (Greece, Scientific Director of Bronchoscopy & Interventional Pulmonology Unit in Metropolitan Hospital),” Personal communication, Moderated by CRA.

7 “Expert Interview with Dr. Sofia Lampaki (Greece, Reader in Lung Cancer - Respiratory Medicine Aristotle University),” Personal communication, Moderated by CRA.

8 “Expert Interview with Dr. Eugenio Paci (Italy, PI of the ITALUNG RCT),” Personal communication, Moderated by CRA.

9 “Expert Interview with Mr. Sebastien Schmidt (Germany, Siemens Healthineers AG),” Personal communication, Moderated by CRA.

10 “Expert Interview with Dr. Annemiek Snoeckx (Belgium, PI of ZORALCS),” Personal communication, Moderated by CRA.

11 “Expert Interview with Dr. Juan Carlos Trujillo (Spain, Co-Coordinator of CASSANDRA Pilot),” Personal communication, Moderated by CRA.

12 “Expert Interview with Ms. Eleanor Wheeler (Health Policy Parthership Which Providers Secretariat for the Lung Cancer Policy Network),” Personal communication, Moderated by CRA.

13 “Expert Interview with Mr. Ivica Belina (Croatia, President of the Coalition of Associations in Healthcare.

14 GOV.UK, “New Lung Cancer Screening Roll out to Detect Cancer Sooner.

15 “Expert Interview with Dr. David Baldwin (England, Chair of the UK Clinical Expert Group for Lung Cancer).”

16 Progetto-Della-Rete-Italiana-Screening-Polmonare.Pdf. https://www.myecole.it/ooc/wp-content/uploads/2021/02/Progetto-della-Rete-Italiana-Screening-Polmonare.pdf

17 “Expert Interview with Dr. Juan Carlos Trujillo (Spain, Co-Coordinator of CASSANDRA Pilot).”

18 Expert Interview with Dr. Sofia Lampaki (Greece, Reader in Lung Cancer - Respiratory Medicine Aristotle University).

19 Zalacznik_17_Ogolnopolski_Program_WWRP.Pdf,” https://www.power.gov.pl/media/72320/Zalacznik_17_Ogolnopolski_Program_WWRP.pdf

20 Lung Cancer Screening - Current Situation in Sweden - Lung & Allergy Forum.

21 Poland Lung Cancer Screening Protocol - Ogólnopolski Program Wczesnego Wykrywania Raka Płuca (WWRP) Za Pomoca Niskodawkowej Tomografii Komputerowej (NDTK) – Połaczenie Prewencji Wtórnej z Pierwotna w Celu Poprawy Swiadomości Dotyczacej Raka Płuca Wśród Społeczeństwa i Personelu Ochrony Zdrowia.

22 Croatia National Protocol - NACIONALNI PROGRAM PREVENCIJE RAKA PLUCA.

23 Croatia National Cancer Control Plan 2020−2030.

24 The NHS long term plan.

25 Expert Interview with Dr. Annemiek Snoeckx (Belgium, PI of ZORALCS).

26 Lungcancerscreening - RCC Kunskapsbanken.

27 University Hospital, Antwerp, "Implementation of Lung Cancer Screening in the First Line Zone of ZORA (Flanders), using a low dose CT-scan.

28 Ενημερωτικό σημείωμα για τη σύσκε*Ppsi*η υπό τον Πρωθυπουργό Κυριάκο Μητσοτάκη με αντικείμενο τις προληπτικές εξετάσεις και την πρωτοβάθμια ϕροντίδα *|* Ο Πρωθυπουργός της Ελληνικής Δημοκρατίας.

29 Lidia Garcia Perez, “Evaluación del programa de cribado de cáncer de pulmón,” *Ministerio de Sanidad, Santa Cruz de Tenerife: Servicio Canario de la Salud, Santiago de Compostela: Agencia Gallega para la Gestión del Conocimiento en Salud, ACIS*.

30 Arnold, “Lung Cancer Screening in Poland.

31 Ministerstwo Zdrowia, “Narodowa Strategia Onkologiczna - Ministerstwo Zdrowia - Portal Gov.pl.

32 Expert Interview with Dr. David Baldwin (England, Chair of the UK Clinical Expert Group for Lung Cancer)” (Personal communication).

33 Charlotte Wickens, *Why Do Diagnostics Matter?*

34 Considerations to ensure optimum roll-out of targeted lung cancer screening over the next 5 years.

35 Tumore al polmone, è tempo di pensare a uno screening nazionale.
