# Supplementary material for: From aspiration to reality: a cross-country analysis of the implementation of lung cancer screening in Europe
Source: Front Public Health. 2026 Apr 1;14:1766560. doi: 10.3389/fpubh.2026.1766560 (PMC13081776; doi:10.3389/fpubh.2026.1766560)
Supplement: Supplementary file 1 [file Data_Sheet_1.docx]

From aspiration to reality: A cross-country analysis of the implementation of lung cancer screening in Europe

Supplemental Material A: Written summary of country profiles

Manuscript for submission to Frontiers in Public Health

2026

# 1. Appendix – Summary A: Written summary of country profiles

## Croatia

***Background / Progress***

Following the development and publication of screening guidelines in 2019 by the Ministry of Health and medical societies, Croatia became the first European country to formally launch a national LCS program in 2020.^[[1]](#endnote-2),^^[[2]](#endnote-3)^ As of 2023, the government announced that more than 22,000 people had been screened with 1,000 people suspected of having lung cancer. Although there have been internal evaluations of the program, results from these evaluations have not been published and no external evaluations were identified. Moreover, the fragmented healthcare system and lack of a clear network of oncology institutions responsible for post-screening follow-up and treatment has resulted in long waiting times.^[[3]](#endnote-4)^

***Framework Assessment***

*Policy Prioritisation and Governance*

Although Croatia spends 11% more on cancer treatment compared to the EU average, it has the second highest cancer mortality in the EU.^[[4]](#endnote-5),^^[[5]](#endnote-6)^ In order to reduce mortality, the country’s first comprehensive national cancer plan (NCP) outlining almost 150 objectives for cancer was approved by parliament and adopted in December of 2020.^[[6]](#endnote-7)^ That same year, Croatia became the first EU country to fully implement a LCS programme with the poor lung cancer outcomes and positive NLST and NELSON data as driving factors for its adoption.^[[7]](#endnote-8)^

Advocacy by key cancer centres and medical societies was essential in developing the national guidelines and moving forward the debate of LCS towards implementation. Leaders of the national LCS programme emphasize that lobbying the government was key in creating the political will for the roll-out of the program.^[[8]](#endnote-9)^

*Clinical Review*

The Ministry of Health recommended LCS based on the results of NLST and NELSON as well as the recommendations of various institutions world-wide. Due to the additional time and effort needed to apply for EU grants and conduct local studies, decision-makers did not believe a local pilot was necessary in light of the international data. Although risk assessment models have not yet been integrated, the national guideline notes that the use of these models can help reduce the number of false positive findings and increase the profitability of the program.^[[9]](#endnote-10)^

*Program Design*

The NCCP outlines that LCS with LDCT will be performed three times in the first phase (first 5 years) of the program for eligible persons who have an initial normal LDCT scan while those with scans showing emphysema features will be scanned every two years. Moreover, it clearly lays out targets for the program over a 10-year period including (1) reduction of lung cancer mortality by 25%, (2) achieving a 60% screening response rate in the target population, (3) raising the five-year survival rate from 6% to 15%.^[[10]](#endnote-11)^

*Implementation*

The 2019 national guideline outlined several implementation steps including implementation indicators and assigned responsibility to specific stakeholders which aided in the prompt roll-out of the program.^[[11]](#endnote-12)^ The budget for implementation comes from both the state budget of the Ministry of Health and the Croatian Institute for Health Insurance; however, it is not clear whether sufficient funding has been allocated for the long-term.^[[12]](#endnote-13)^ Although there are currently 20 accredited centres for screening which has been sufficient for the program thus far, it is uncertain whether the current resources (e.g., radiologists, CT scans) will be enough to sustain the program as it expands as well as meet the surge in downstream services.^[[13]](#endnote-14)^

*Awareness*

GPs and community nurses are at the centre of the national strategy to reach-out to the eligible population with financial incentives in place to drive participation of GPs.^[[14]](#endnote-15),^^[[15]](#endnote-16)^ Nevertheless, there is a need for more public awareness campaigns in order to combat stigma and increase participation among the general public.^[[16]](#endnote-17)^

## England

***Background / Progress***

In June 2023, building upon the successful National Health Services England pilot Targeted Lung Health Checks (TLHC) programme and the UK NSC recommendation, the Prime Minister announced the rollout of a national targeted LCS program which will require £270 million per year once implemented. The government has committed to reach 40% of the eligible population by March 2025 and aims to achieve full coverage by March 2030.^[[17]](#endnote-18)^ As of September 2024, over 2 million invitations had been sent for the TLHC and 958,109 lung health check were attended. Moreover, 589,292 scans were conducted with 5,271 lung cancers diagnosed. Of the lung cancers diagnosed, 62% were Stage 1, 13% were Stage 2, 13% were Stage 3 and 9% were Stage 4 supporting the distribution of diagnosed cancers in early stage (76% identified in stage 1 or 2).^[[18]](#endnote-19)^ So far, there has been a 57% uptake for lung heath checks and in the recent independent review of the NHS conducted in July 2024, Lord Darzi’s report suggests that notable improvements have been made in early detection of cancer in the UK since 2021, citing significant contributions from the TLHC program.^[[19]](#endnote-20)^

Despite government commitments for a national program, there are still some uncertainties in the transition between the NHSE Targeted Lung Health Checks and the proposed national screening program as well as the scope of this program (i.e., all the UK or only England).

***Framework Assessment***

*Policy Prioritisation and Governance*

Since 2019, NHSE has funded the expansion of the TLHC programme to 14 regions in England with the highest lung cancer mortality rates. The NHS included this expansion as a strategy for improving lung cancer outcomes in its 10-year Long Term Health Plan.^[[20]](#endnote-21)^ Apart from NHS commitment, there has been parliament commitment to LCS which was most evident in the announcement of the roll-out of the national program in 2023.^[[21]](#endnote-22)^

Academic and medical societies were crucial in the pilots and moving forward the debate. Several charitable organizations such as the Roy Castle Lung Cancer foundation and MacMillan Cancer Support have been part of evidence generation through funding local LCS pilots.

*Clinical Review*

The UK NSC reviewed the evidence package including nine RCTs, of which only the NLST and NELSON trials were considered adequately powered to evaluate clinical effectiveness of LCS. Nevertheless, UK NSC evaluated two local RCTs, LSUT and UKLS, as well as various UK pilot studies and meta-analyses to understand the local acceptability of a screening program, a key parameter in the evaluation.^[[22]](#endnote-23)^ In determining the eligibility criteria and risk prediction model, the standard protocol developed for the TLHC program in 2019 was followed which recommended the use of LLPversion2 and PLCOm2012 as they were the only validated risk prediction models at that time.^[[23]](#endnote-24)^

*Program Design*

Following the 2019 TLHC protocols, current and former smokers will be identified through GP records and high-risk individuals will be offered a LDCT; however, this will require for comprehensive and data completeness in primary care records.^[[24]](#endnote-25),^^[[25]](#endnote-26)^ Individuals considered high risk will be invited to scans every two years until they surpass the higher age limit. Once fully implemented, it is estimated that 992,000 LDCT scans would be performed each year with the program detecting cancer in as many as 9,000 people per year.^[[26]](#endnote-27)^ While the national program has already started, screening rate targets have yet to be determined and goals for early detection, five-year survival and lung cancer outcomes are still to be established.

*Implementation*

Despite the announced roll-out, there has been no public assessment for the expected surge in demand for diagnostics and treatment expected due to screening. It is expected that total spending will be held flat from 2025-2026 onwards despite inflation concerns which may create additional funding hurdles.^[[27]](#endnote-28)^ Moreover, experts responsible for local implementation of the programs highlight that the government has issued a capital freeze which makes it impossible for screening centres to acquire or lease the necessary equipment, including CTs, for the implementation and scaling of a screening program. Furthermore, currently, centres have to seek funding from three different sources: NHSE, ICBs, and specialized commissioning so a more centralized and timely funding system is essential.^[[28]](#endnote-29)^

Even more so, the workforce and capacity constraints affecting the NHS pose the biggest challenges. Currently, there is concern over the imaging workforce shortage with greater attrition of radiologists expected in the next 5 years.^[[29]](#endnote-30)^ The UK has 8.8 CT scanners per million which is lower to the OECD average of 25.9 yet demand has surged by over 25% over the past 5 years.^[[30]](#endnote-31)^ There are several workforce pressures faced by NHSE which will require developing workforce capacity and employing technology when available in order to maximise efficiency. There has been no assessment on the healthcare system impact of a national screening program with a limited diagnostic capacity and workforce potentially leading to bottle-necks in the patient pathway and reduced access to timely treatment.^[[31]](#endnote-32)^

*Awareness*

Several charitable organizations such as the Roy Castle Lung Cancer foundation and MacMillan Cancer Support have engaged in public awareness campaigns for the TLHC program.^[[32]](#endnote-33),^^[[33]](#endnote-34)^ With the roll-out of a wider program, HCP awareness campaigns on LCS and its benefits will be needed to ensure uptake and adherence of screening, particularly for GPs at the forefront of recruitment; however, formal training and awareness campaigns are yet to be announced and implemented.^[[34]](#endnote-35)^

## Poland

***Background / Progress***

Poland rolled-out the first three-year implementation phase of the national LCS program in 2020.^[[35]](#endnote-36),^^[[36]](#endnote-37)^ In the first phase, 16,946 LDCT scans were performed across 31 implementer sites including six Centres of Excellence.^[[37]](#endnote-38)^ In collaboration with International Early Lung Cancer Action Program (I-ELCAP), a cloud-based platform has been designed to support the delivery of a centralised programme and has been rolled out in all participating sites.^[[38]](#endnote-39)^

The first phase of implementation was co-financed by the Ministry of Health and the European Social Fund under a three-year POWER grant with EU funding in the Siselian micro-region totalling USD 957,0312.^[[39]](#endnote-40)^ As set in the 2020-2030 National Oncology Strategy (NSO), public government funding is expected to start in 2024; however, it is not clear whether a concrete commitment for further funding has been arrived at yet.^[[40]](#endnote-41)^ Moreover, the 2019 National Lung cancer screening Protocol outlined areas for evaluation such as change in mortality rate with an evaluation expected at the end of 2023.^[[41]](#endnote-42)^ However, no public evaluation of the program is available as of mid-2024.

***Framework Assessment***

*Policy Prioritisation and Governance*

The government prioritization of lung cancer outcomes as evidenced by the funding of early regional pilots and incorporation of lung cancer outcome goals in the 2020-2030 NSO was instrumental for the 2020 approval of the phased roll-out of a national programme.^[[42]](#endnote-43),^^[[43]](#endnote-44)^ Moreover, clear roles and responsibility within the Ministry of Health on the approval, implementation and evaluation of a screening program aided in the prompt roll-out of the program following the publication of the National Protocol in 2019.^[[44]](#endnote-45)^

The academic community played a major role in building the political will for the implementation of a national program by engaging with high-profile politicians, running regional pilots, and publishing position papers.^[[45]](#endnote-46)^

*Clinical Review*

The Ministry of Health highlighted two international clinical studies, NLST and the UKLS pilot study, as providing evidence on the effectiveness of LDCT LCS in the 2019 National Protocol for Lung cancer screening. Given that NELSON was ongoing at the time of the protocol development, NELSON results were not available during the clinical review. Moreover, since 2008 to 2019, several LCS pilots were conducted in Szczecin, Gdańsk, Poznań and Warsaw where almost 50,000 people were screened. This pool of local data was also evaluated with the protocol noting the data outlined the scale of the nicotine addiction problem in Poland as well as confirmed the effectiveness of LDCT.^[[46]](#endnote-47)^

*Program Design*

The national protocol outlines LCS through annual LDCT scans for those that meet the eligibility criteria. Given the demographic data and eligibility criteria for the program, the protocol estimated that between 2 to 3 million people would be eligible for screening and that 48,000 tests could be carried out given current capacity. In light of the local data, the national protocol set out to increase awareness on lung cancer prevention & tobacco counselling programs, to train at least 692 medical staff on lung cancer prevention and to increase the number of lung cancer detected at earlier stages by 42% to 65%. Although the protocol listed areas that should be evaluated for the program, it did not outline specific goals or targets for these areas. Furthermore, there is no formal, published screening rate target in the protocol nor the NSO.^[[47]](#endnote-48)^

*Implementation*

The program is transitioning from EU grants to public funding; however, it is unclear whether enough public funding has been dedicated to maintaining and expanding the program.^[[48]](#endnote-49)^ Although the program is being rolled out in a phased approach the protocol does not outline a clear implementation plan for the subsequent expansion phases. In terms of the required infrastructure, the national protocol evaluated the medical staff available in Poland and found that there were 2,834 lung specialists and 3,501 radiologists and imaging specialists. However, the protocol did not determine whether the number of HCPs or the number of currently available equipment was enough to sustain the programme in the long-term.^[[49]](#endnote-50)^

*Awareness*

The medical community developed training for radiologists on quality assurance and guidance for general practitioners (GPs) on the evidence and inclusion criteria for the screening program to encourage adoption.^[[50]](#endnote-51)^ In order to raise awareness among the general population, several television stations, radio programmes and news outlets have interviewed key experts and published material on the LCS programme.^[[51]](#endnote-52)^Although public awareness campaigns have been established, low uptake of the national programme and public interest remains a problem.

## Belgium

***Background / Progress***

Although the NELSON trial included participants from the Flemish region of Belgium, implementation of LCS has been quite slow partly due to the decentralized healthcare system.^[[52]](#endnote-53)^ In Belgium, cancer screening is organized and delivered at a regional level so LCS would need to be accepted separately in each region.^[[53]](#endnote-54)^ Of the 3 regions, only Flanders has an organized task force that is advocating and building momentum for the implementation of LCS.^[[54]](#endnote-55)^ In April 2024, the national KCE, a national body that advises public authorities, published its HTA report on LCS which concluded that it was cost-effective but recommended authorities to consider further factors (e.g., budget impact and capacity).^[[55]](#endnote-56)^

***Framework Assessment***

*Policy Prioritisation and Governance*

There is minimal political will in Belgium for LCS which is further exacerbated by the decentralized decision-making. To date, apart from the Flanders region, no other region in Belgium has reviewed the potential for a screening program. Moreover, the NCP was last published in 2008 and lacks updated targets for lung cancer.^[[56]](#endnote-57)^ It is uncertain whether an NCP will become a priority with the new government.^[[57]](#endnote-58)^

A group of Flemish Key Scientific Leaders (KSLs) formed the Flemish Lung cancer screening Taskforce which has been instrumental in advocating for LCS in Flanders and organizing a regional implementation study. Moreover, the taskforce hopes to submit a dossier to the Flemish government by 2026 for LCS.^[[58]](#endnote-59)^ No similar task force nor has been organized in other regions.

*Clinical Review*

The KCE reviewed evidence from systematic reviews and RCTs and concluded that LCS is effective in reducing lung cancer-specific and overall mortality. Nevertheless, the report also highlighted the potential harms and recommended that the Federal Agency for Nuclear Control (FANC) conduct a study on radiation protection as is compulsory before LCS can be introduced.^[[59]](#endnote-60)^ Along with a positive KCE report, regional implementation studies are needed for the adoption of LCS.^[[60]](#endnote-61)^

*Program Design*

In 2024, a Flemish feasibility study in Antwerp began. The study is expected to have the first round of scans in 2025 and to finish by 2027. There are no similar initiatives for implementation studies in the other regions.^[[61]](#endnote-62),^^[[62]](#endnote-63)^ As part of their report, the KCE conducted a cost-effectiveness analysis which found LCS cost-effective if the willingness to pay is between €20,000-€30,000 per quality-adjusted life years (QALY) gained. Furthermore, the report recommended conducting a budget-impact analysis if LCS were to be adopted. ^[[63]](#endnote-64)^

*Implementation*

Given there is no recommendation for LCS implementation yet, there is no dedicated government funding nor implementation plan. Since population-based screening programs are implemented at a regional level, the implementation timelines may vary by region. For example, colorectal cancer screening was implemented in Brussels in 2002 and in Flanders until 2013.^[[64]](#endnote-65)^ Furthermore, the KCE recommended that the organisational impact in terms of availability of infrastructure and required capacity should be estimated and considered when deciding on LCS.^[[65]](#endnote-66)^

*Awareness*

Stigma as well as low awareness of LCS among GPs may limit participation rates.^[[66]](#endnote-67)^ Public perception of screening was assessed in the 2020 Smoking Survey by the Belgian Foundation Against Cancer which found that only 54% of respondents were aware of screening initiative.

## Germany

***Background / Progress***

In Germany, the legal framework establishes two independent decision makers for lung-cancer screening: the Federal Ministry of the Environment, Nature Conservation, Nuclear Safety and Consumer Protection (BMUV) who makes decisions regarding radiation protection and the Federal Joint Committee of Health Insurances and Providers (G-BA) who makes decisions regarding reimbursement and implementation.^[[67]](#endnote-68)^ On July 1, 2024, the BMUV’s Lung Cancer Early Detection Ordinance came into force which legalizes LCS and outlines the minimal technology requirements for its safe use.^[[68]](#endnote-69)^ The next step for a national LCS program will be for the G-BA to decide on the reimbursement and implementation as pending the decision, LDCT screening would only be accessible out-of-pocket.^[[69]](#endnote-70)^

***Framework Assessment***

*Policy Prioritisation and Governance*

Although the G-BA consultation process on implementation guidelines started early 2024 with the aim of delivering a formal G-BA decision within 18 months of the publication of the BMUV ordinance as dictated by the law, there is concern that the G-BA decision will be delayed.^[[70]](#endnote-71),^^[[71]](#endnote-72)^ Moreover, medical societies have expressed concern with the lag time between BMUV ordinance and G-BA’s implementation guideline as it may lead to screenings outside of a structured, quality-assured program.^[[72]](#endnote-73)^ Specialty societies such as the German Society for Pneumology and Respiratory Medicine and the German Society for Thoracic Surgery were key in pushing the debate through the publication of multiple position papers.^[[73]](#endnote-74)^

*Clinical Review*

Both IQWIG and BfS, recommending bodies to the G-BA and BMUV, respectively, recommended screening with LDCT after reviewing the clinical effectiveness in 8 RCTs, including one German study.^[[74]](#endnote-75),^^[[75]](#endnote-76)^ Following this evaluation, the BMUV recommended LCS for people ages 55-74 who are ever-smokers and who have (1) consumed cigarettes for at least 25 years and at least 15 pack-years or (2) ongoing cigarette consumption or quit smoking less than 10 years ago.^[[76]](#endnote-77)^ In order to ensure the optimal benefit-risk ratio and protection from radiation, the BfS recommended incorporating a risk model that can more accurately assess individual cancer risk.^[[77]](#endnote-78)^ To this end, the ongoing HANSE study is evaluating the NELSON model and the PLCO_m2012_ model for use in Germany.^[[78]](#endnote-79)^

*Program Design*

The program will use LDCT scans and the BMUV ordinance outlines the technical requirements to ensure an acceptable benefit-risk ratio.^[[79]](#endnote-80)^ Although an official interval has not been established, the BMUV indirectly recommended an annual screening interval. Medical societies are also in favour of an annual interval; however, G-BA may decide otherwise depending on the availability of the necessary technical and personnel resources.^[[80]](#endnote-81)^ Moreover, despite having an early cancer detection law since 2013, there are no clear national targets for lung cancer nor LCS.

*Implementation*

It is expected that the national LCS programme may start to be implemented as early as 2025. To date, no formal announcements have been made on funding nor the implementation as guidelines are yet to be developed and published by the G-BA. The BfS and medical societies recommend the national lung cancer programme to leverage the 73 established lung cancer centres and to create an interdisciplinary network with certified outpatient and inpatient partners.^[[81]](#endnote-82)^ Moreover, medical societies have proposed a detailed organization of the structure for LCS; however, the scalability and sufficiency of resources remains unknown.^[[82]](#endnote-83)^

*Awareness*

As of September 2024, no physician awareness campaigns were identified; however, specialty societies acknowledge that training and appropriate certification will be needed for general practitioners and other HCPs who will play an important role in the identification and referral of patients to the program.^[[83]](#endnote-84)^ Similarly, no public awareness campaigns were identified. Given the BMUV ordinance, it is expected that formal trainings and awareness campaigns will be developed soon.

## Italy

***Background / Progress***

Since the early 2000s, several Italian RCTs including the Multicentric Italian Lung Detection (MILD), ITALUNG and DANTE trials have shown the effectiveness of LCS with LDCT. The last HTA and evidence review was in 2014 and resulted in a negative recommendation for LCS.^[[84]](#endnote-85)^ In 2021, the Italian Lung Screening Network (RISP) project was launched as a national pilot to evaluate the implementation of a LCS program throughout the national territory and demonstrate a significant reduction in lung cancer mortality.^[[85]](#endnote-86)^ The RISP project is expected to be completed in 2024 and results published in 2026.^[[86]](#endnote-87)^ Moreover, there is a second pilot project, that is coordinated by ISPRO and funded by the Italian Ministry of Health’s Centre for Disease Control and Prevention (CCM). This pilot project has enrolled 1143 subjects and is currently in the process of publishing results.^[[87]](#endnote-88)^

***Framework Assessment***

*Policy Prioritisation and Governance*

The National Oncology Plan 2023-2027 was adopted in January 2023 and discusses the updated European Council recommendations on screening for lung cancer.^[[88]](#endnote-89),^^[[89]](#endnote-90)^ Moreover, in July 2021, the Ministry of Health budgeted €1 million euros for each of the years 2021 and 2022 to be allocated to the centres of the RISP project.^[[90]](#endnote-91)^ The RISP pilot will be able to obtain new European funding in 2024 through its involvement in the European 4-ITLR and SOLACE LCS research projects. However, experts emphasize that more funding is necessary to allow RISP to expand its activities in the next two years, calling on the Ministry of Health to allocate further funding.^[[91]](#endnote-92)^ Moreover, an updated HTA review has yet to be announced which may delay decision-making on a national LCS program. The Alliance for Lung Cancer Advocacy, Support and Education (ALCASE) has been advocating for free LCS since 2015. ALCASE have written open letters to the Ministry of Health and have collected over 18,000 signatures on a petition.^[[92]](#endnote-93),^^[[93]](#endnote-94)^

*Clinical Review*

Since the 2014 HTA review published prior to the results of NELSON, there has been no published formal evaluation of the NELSON or NLST data.^[[94]](#endnote-95)^ However, the Ministry of Health has expressed a preference in the European data from trials such as NELSON.^[[95]](#endnote-96)^ It is unclear whether local data will be needed for decision-making in Italy. Nevertheless, there have been several studies conducted in Italy including the COSMOS 10-year non-randomised trial evaluating LDCT in Milan and several randomized control trials such as the MILD study (4,999 participants), the ITALUNG trial (3,206 participants), and the DANTE study (2,450 participants).^[[96]](#endnote-97)^ The three RCTs were included in the 2014 HTA evaluation; however, it was noted that not all data from these trials was available at the time of the evaluation.^[[97]](#endnote-98)^

*Program Design*

The RISP program has been underway since 2021 and will screen 10,000 individuals over 18-24 months.^[[98]](#endnote-99)^ As of November 2023, approximately 9,400 scans have already been carried out and between recruitment and registration, approximately 19,000 people have been reached.^[[99]](#endnote-100)^ In terms of cost-effectiveness, a 2020 study found a favourable cost-effectiveness ratio for LCS in Italy in smokers (≥30 pack-years) of 55–79 years.^[[100]](#endnote-101)^ In addition, one of the secondary objective of the RISP program is to evaluate the costs and cost-effectiveness of each arm of the study, which differs by screening frequency.^[[101]](#endnote-102)^

*Implementation*

There is no dedicated government funding nor an implementation timeline at this time for the implementation of a LCS program as there is currently no positive recommendation for its implementation from the health authorities. Furthermore, there are no guidelines on recruitment nor studies on the scalability of LCS including the impact on the workforce and the infrastructure capacity for screening and downstream services or treatment.^[[102]](#endnote-103)^ Although, there are 18 participating centres in the RISP program, made up of IRCCS, university centres and hospitals which cover a large part of the national territory, more centres may be needed for a full-scale national program.^[[103]](#endnote-104),^^[[104]](#endnote-105)^

*Awareness*

Moreover, several stakeholders, including industry and NGOs, have launched public campaigns to increase awareness of lung cancer and underscore the availability of the current RISP pilot program.^[[105]](#endnote-106)^ In terms of physician awareness and training, organizers of RISP hope to develop guidelines and training programs for LDCT screening and management.^[[106]](#endnote-107)^

## Spain

***Background / Progress***

In 2021, the Spanish HTA agency, RedETS, started plans to re-evaluate LCS following the publication of the NELSON data.^[[107]](#endnote-108)^ That same year, nine medical societies along with several patient advocacy groups (PAGs) announced the first 5-year national pilot program, CASSANDRA, which started in 2023.^[[108]](#endnote-109)^ In August 2023, RedETS published a negative recommendation for LCS concluding that there was no sufficient evidence for the effectiveness, safety and cost-effectiveness of LCS and that it only met 6 out of the 18 criteria for a national screening program. However, the RedETS report recommended that additional evidence be collected through local pilot programs.^[[109]](#endnote-110)^

***Framework Assessment***

*Policy Prioritisation and Governance*

There is little national political will and commitment for LCS. Although leaders of the CASSANDRA project have pressed the national government for financial support of the national pilot, support has been minimal with charitable organizations funding most of it.^[[110]](#endnote-111),^^[[111]](#endnote-112),^^[[112]](#endnote-113)^ Moreover, the negative HTA recommendation is a limiting factor for the integration of LCS into the portfolio of common services which, by law, all regions reimburse.^[[113]](#endnote-114)^ Given the low commitment from the national government, the autonomous communities of Galicia and Madrid have announced support and pursual of local pilots to provide evidence for reimbursement in the regional complementary portfolio.^[[114]](#endnote-115),^^[[115]](#endnote-116)^

Medical societies, PAGs, the Lung Ambition Alliance and several charitable organizations have been at the forefront in advocating for LCS, organizing the national pilot program, and creating public awareness campaigns.^[[116]](#endnote-117),^^[[117]](#endnote-118)^ Following the negative HTA evaluation, medical societies and PAGs emitted statements and were vocal in their disapproval of the evaluation.^[[118]](#endnote-119),^^[[119]](#endnote-120)^

*Clinical Review*

RedETS included 9 RCTs in their evaluation and concluded that although there is a slight reduction in disease-specific mortality, there was insufficient evidence of a global mortality benefit. Moreover, the report highlighted the lack of local data as there was no available data from CASSANDRA.^[[120]](#endnote-121)^ As of February 2024, CASSANDRA has started in 5 centres with it expected to expand to over 40 public hospitals across all regions of Spain, except Galicia which is conducting its own pilot.^[[121]](#endnote-122)^ Current evaluations and the pilot study in Spain are defining eligibility criteria with age and smoking status while hoping to collect enough data to create a Spanish risk model.^[[122]](#endnote-123)^

*Program Design*

In its report, RedETS evaluated the feasibility and organizational aspects of a LCS program and noted limited evidence of local feasibility. Moreover, a cost-effectiveness was conducted which reported a lack of cost-effectiveness, a key factor in the negative recommendation.^[[123]](#endnote-124)^ Given no positive decision on LCS, target screening rate and goals have yet to be defined.

*Implementation*

There is no dedicated government funding despite CASSANDRA only having funding for the first 2 years of the program.^[[124]](#endnote-125)^ Moreover, there is limited evidence on the necessary infrastructure for LCS as RedETS reports there is currently not enough human or material resources to sustain a national program. Moreover, the report highlights the absence of indicators and digital system to measure the outcomes of a program.^[[125]](#endnote-126)^

Awareness

Established physician training programs have not been established due to the lack of a positive HTA outcome. Patient advocacy groups (AEACaP and the Spanish Group of Cancer Patients – GEPAC) and the Lung Ambition Alliance have created several public awareness campaigns such as #PonleCaraATiempo and #TardeoTemprano through social media outlets to motivate stopping of tobacco consumption and early lung cancer detection programs.^[[126]](#endnote-127)^

## Sweden

***Background / Progress***

In Sweden, although a screening program is recommended at the national level, each of the 21 regions decides on the implementation and is responsible for financing and providing health services. In addition, there are 6 regional cancer centres (RCC) and the Confederation of RCCs in Sweden which allows cooperation among them coordinates the implementation of screening programs. ^[[127]](#endnote-128)^

In 2022, the the National Board of Health and Welfare (Socialstyrelsen or SoS), a recommending body to the MoH and regional authorities, called for local implementation studies and has decided to not further investigate LCS until further evidence is provided.^[[128]](#endnote-129)^ In 2023, RCC Stockholm-Gotland started the first pilot study which had been delayed since 2018.^[[129]](#endnote-130)^

***Framework Assessment***

*Policy Prioritisation and Governance*

Although lung cancer has not been a priority in national policy, an updated national cancer strategy expected in 2024 may increase political commitment.^[[130]](#endnote-131),^^[[131]](#endnote-132)^ Nevertheless, decentralization remains a barrier to prompt decision making and homogenous implementation of screening. Moreover, implementation of LCS remains at a crossroad until enough evidence is gathered and the SoS issues a positive recommendation.^[[132]](#endnote-133)^

Since 2019, academic communities, notably SLUSG, have been in dialogue and pressuring the National Board of Health and Welfare to recommend a national LCS program.^[[133]](#endnote-134)^ SLUSG holds yearly meetings to discuss progress of the program and hold joint meetings every three years with other interested HCPs in the Nordics regions.^[[134]](#endnote-135)^

*Clinical Review*

Although the SoS does not question the clinical evidence; it has considered that LCS does not meet the criteria for further investigation of a general recommendation mainly due to the screening population being determined by a lifestyle factor.^[[135]](#endnote-136)^ The SoS has recommended feasibility studies to be conducted to better understand LCS in the Swedish context but does not expect all RCCs to conduct feasibility studies.^[[136]](#endnote-137),^^[[137]](#endnote-138)^ In particular, the SoS would like to see evidence on how to reach the at-risk population, the proportion who meet the screening criteria, the proportion and frequency of false positives, among other data.^[[138]](#endnote-139)^

*Program Design*

There is currently one pilot project underway organised by RCC Stockholm-Gotland and several other are being planned by RCC Syd, RCC Norr and RCC Vast. It is expected that each feasibility study would run for 2 years and screen 1000 people with the RCC Sotckholm-Gotland pilot anticipated to end in 2027. The studies are expected to follow inclusion criteria as the planning group’s study synopsis. The first pilot program was planned to screen women ages 55 to 74 with increased risk of lung cancer (at least 30 pack years). As of November 2023, the first pilot has screened 650 women with 6% being referred to an oncology centre. RCC Stockholm has agreed to expand regional pilot for 3 more years and include 1000 more individuals, both women and men. ^[[139]](#endnote-140)^

*Implementation*

As part of their evaluation, the SoS will assess the resources needed and whether there is the required infrastructure for a program; however, to date there has been no formal evaluation of the needed resources and scalability.^[[140]](#endnote-141)^ Although Sweden performs high in health indicators, waiting times are still an issue with wait time and quality of care differing across regions.^[[141]](#endnote-142)^ The limited number of radiologists and decentralized IT systems remain a barrier for implementation.^[[142]](#endnote-143)^

*Awareness*

Currently, no physician training programs, or awareness campaigns were identified, Sweden has collaborated with the United Kingdom to identify learnings from their experience with Lung Health Checks and LCS that could be applied in the Swedish context. One of the learnings being explored is suggesting the screenings as a “wellness” check in order to increase participation rates in high-risk individuals who might otherwise not participate as well as reduce feelings of guilt associated with smoking.^[[143]](#endnote-144)^

## France

***Background / Progress***

In February 2021, the Cancer National Institute (INCa) and President Macron launched the 10-year National Cancer Plan where there was a commitment to the assessment of a LCS programme.^[[144]](#endnote-145)^ The following year, the National Health Authority (HAS) updated their 2016 evaluation on LCS with LDCT which had previously resulted in a negative recommendation. Given the data showing a reduction in lung cancer-specific mortality. HAS recommended INCa to implement a pilot programme with the objective of gathering local data to answer outstanding questions.^[[145]](#endnote-146)^

***Framework Assessment***

*Policy Prioritisation and Governance*

One of the goals of the 2021-2029 French Ten-Year Cancer Control Strategy published by INCa and presented by President Macron was the “establishment of an organised LCS programme, once the data indicate a favourable benefit-risk balance.” Moreover, during the announcement of this ten-year strategy, President Macron promised €1.74 billion across 2021-2025 to finance the goals and actions proposed in the cancer plan with almost half of the budget dedicated to research. This financial commitment was 20% higher commitments made for the past three cancer plans.^[[146]](#endnote-147),^^[[147]](#endnote-148)^ Moreover, The Ministry of Health and the National Institute of Cancer awarded €1.8 million to the CASCADE national trial studying LCS in women who investigator highlight are underrepresented in most LCS studies.^[[148]](#endnote-149)^ Nevertheless, the financial investment and potential low participation rate will be major concern for decision-makers.^[[149]](#endnote-150)^Medical societies have been collaborating in local LCS research as well as advocating for the implementation of a LCS programme.^[[150]](#endnote-151)^

*Clinical Review*

HAS analysed 4 systematic reviews, 3 of which were meta-analyses, out 46 publications well as 4 international recommendations (UPSTF, American College of Chest Physician, EUnetHTA, INESSS) out of 39 published between 2016-2021 for the update of their 2016 assessment of LCS with LDCT. HAS criticized the comparison against radiography in the NLST study as no screening is the standard in France and concluded that the conditions of the study were not representative of the French context. From reviewing the new evidence, HAS concluded that LCS Lung cancer showed a significative reduction of lung cancer-specific mortality and a significative reduction of cancers detected at an advanced state compared to no screening. Currently, evidence is still incomplete and insufficiently robust for the implementation of an organized LCS with LDCT programme and recommended a national local pilot.^[[151]](#endnote-152)^ INCa is developing national guidelines for the pilot programme which are expected by the end of 2023.^[[152]](#endnote-153),^^[[153]](#endnote-154)^

*Program Design*

HAS noted that the heterogeneity of the clinical trials makes it impossible to define a screening protocol or define the most appropriate method for an organized screening programme.^[[154]](#endnote-155)^ Therefore, one of the objectives of the pilot programme and the complementary studies to be defined by INCa is to refine the eligibility criteria and recruitment strategy.^[[155]](#endnote-156)^ In addition, a study led by the International Agency for Research on Cancer estimated the screening benefits for different eligibility criteria. The study found that depending on the eligibility criteria applied, 2 to 4 million individuals could be eligible for screening in France and 11,100 to 14,200 lung cancer deaths may be preventable over 5 years.^[[156]](#endnote-157)^ Moreover, INCa underscored that additional studies on LCS could include evaluating the economic impact of systematic LCS; however, it has yet to announce any studies.^[[157]](#endnote-158)^

*Implementation*

Funding has been dedicated for the national cancer plan which is responsible of implementing a pilot programme; however, no funding has been announced for the implementation of an organized LCS programme as it has not been yet recommended by HAS.^[[158]](#endnote-159)^ Moreover, HAS noted that for the implementation of an organized LCS programme, it is necessary to have an information system capable to gather all data and deliver results as well as analyse the impact a screening programme would have on the health system in particular the equipment and staffing needed.^[[159]](#endnote-160)^ However, feasibility studies have yet to determine if the current system can support a national screening programme or the requirements that are needed to be addressed prior to its implementation.

*Awareness*

In 2020, the National Federation of Radiologists (FNMR) campaigned and called on the health authorities to act on LCS with LDCT and were joined by the National Union of the Respiratory Apparatus (SAR) and the National Union of Radiotherapists (SNRO) for a similar campaign in 2021.^[[160]](#endnote-161),^^[[161]](#endnote-162)^ Moreover, the Thoracic Imaging Society offers e-learning modules and workshops for all French radiologists that desire to get training for LCS; however training for GPs and other specialists remains limited.^[[162]](#endnote-163)^ In addition, in 2021, a collective of lung cancer specialists (e.g., oncologists, radiologists) and patient associations launched the “Ensemble Nous Poumons” campaign aimed at raising awareness for lung cancer including early detection benefits and evidence.^[[163]](#endnote-164)^

## Greece

***Background / Progress***

In 2020, the private Metropolitan Hospital in Athens launched the first organized screening program for early diagnosis of lung cancer in Greece, offering screening at a low-cost or free for vulnerable people.^[[164]](#endnote-165)^ Between October 2021 and April 2023, the Hellenic Task Force on Lung Cancer Screening drafted the Guidelines and Proposed Screening Procedure for Lung Cancer.^[[165]](#endnote-166)^ In January 2022, a bill was adopted by the Ministry of Health approving the National Prevention Program “Spyros Doxiadis”, a program based on prevention and the coordination and interconnection of all relevant health services in the country.^[[166]](#endnote-167)^ By May 2023, the Ministry of Health announced a LCS pilot program using LDCT across four hospitals.^[[167]](#endnote-168)^ However, the start of the pilots was delayed, with the program expected to start in 2025.^[[168]](#endnote-169)^

***Framework Assessment***

*Policy Prioritisation and Governance*

Although the Minister of Health emphasized that preventative screening programmes were the main vehicle to prevent cancer in a roundtable discussion on the Europe’s Beating Cancer Plan, limited action has been taking to prioritize LCS.^[[169]](#endnote-170)^ Greece currently does not have a national cancer plan as the previous plan expired in 2015.^[[170]](#endnote-171)^ The Ministry of Health has stated an updated national cancer plan is in progress with a finalised plan expected in 2025.^[[171]](#endnote-172)^ Despite a commitment to LCS pilots, there is great uncertainty surrounding the status of these pilots under the "Spyros Doxiadis“ Program. The total budget for this program covering prevention and screening programs is over €300 million, funded by the Recovery and Resilience Fund Greece 2.0 and the Next Generation EU plan.^[[172]](#endnote-173)^ However, it is not clear yet how much would be allocated to a LCS program. Medical societies have been at the forefront of pushing forward the LCS debate and the Hellenic Task Force on Lung Cancer Screening has created guidelines for LCS.^[[173]](#endnote-174)^

*Clinical Review*

There has been no formal government evaluation of LCS. The Fairlife foundation funded a study modelling the impact of a LCS program in Greece which used the NLST and NELSON trials as benchmarks.^[[174]](#endnote-175)^ The results of the study were presented in a 2021 conference which had the Deputy Ministry of Health in attendance.^[[175]](#endnote-176)^ Moreover, previous cancer screening programs did not appear to require detailed local feasibility studies for implementation and rather were based on evidence and official guidelines from various large international agencies such as the American Cancer Society and the NHS breast and cervical screening programs. ^[[176]](#endnote-177)^

*Program Design*

In addition to the pilots announced by the Ministry of Health under the “Spyros Doxiadis” program, as of 2024, there have been two LCS studies in Greece. A pilot study from January 2016 to March 2022 assessed 1500 individuals aged >45 years with smoking history of at least 15 “pack years”. In this study, adherence to screening was over 90% and it was concluded that real-world data agreed with other large RCTs, although detailed results are not publicly available.^[[177]](#endnote-178)^ Moreover, the organized screening program organized by the private Metropolitan Hospital in Athens offers screening to current or ex-smokers aged 50 and over who have quit smoking in the last 15 years and have a history of smoking 20-30 pack years. Although the program has been described as a success and awarded an honorary medal in 2022, data from the program is not publicly available yet.^[[178]](#endnote-179),^^[[179]](#endnote-180)^ Furthermore, the guidelines developed by the Hellenic Task Force outline specific technology requirements as well as recommends bi-annual and annual screening intervals for low-risk candidates and high-risk candidates, respectively.^[[180]](#endnote-181)^

*Implementation*

The Guidelines and Proposed Screening Procedure for Lung Cancer published in April 2023 suggest screening should be carried out by accredited tertiary centres or units (either mobile or fixed). The guidelines recommend these tertiary health centres to be responsible for performing LDCT scans within the framework of an official national program, distributing the results to the candidate and referring physician, planning the next follow-up appointment, and organizing the candidate’s smoking cessation program. Moreover, it recommends that the tertiary health centres to also take responsibility for recording the candidate’s epidemiological data using a standardized form and adding this information to the National Cancer Registry which was approved in October 2023 and yet to be implemented.^[[181]](#endnote-182),^^[[182]](#endnote-183)^ Nevertheless, there is limited evidence on the existence of the necessary infrastructure for LCS in Greece.

*Awareness*

The Hellenic Task Force on Lung Cancer Screening has created guidelines for LCS which also include standarised training of healthcare professionals.^[[183]](#endnote-184)^ Moreover, in collaboration with industry and FairLife Lung Cancer Care, a non-profit organization founded in 2020 to raise awareness for lung cancer prevention, have presented data at local conferences as well as created public awareness campaigns such as the “Breath of Life” 2020 campaign and the “Beware of the Gap” 2023 campaign.^[[184]](#endnote-185),^^[[185]](#endnote-186),^^[[186]](#endnote-187)^

1. “Croatia National Protocol - NACIONALNI PROGRAM PREVENCIJE RAKA PLUĆA,” accessed October 15, 2024, https://zdravlje.gov.hr/UserDocsImages/2019%20Programi%20i%20projekti/NACIONALNI%20PROGRAM%20PREVENCIJE%20RAKA%20PLU%C4%86A.pdf. [↑](#endnote-ref-2)
2. “Croatia National Cancer Control Plan 2020 – 2030,” accessed October 15, 2024, https://www.iccp-portal.org/system/files/plans/NPPR_ENG_final.pdf. [↑](#endnote-ref-3)
3. Expert interview with Mr. Ivica Belina (Croatia), Moderated by CRA, Personal communication. [↑](#endnote-ref-4)
4. “State of Health in the EU Croatia Country Health Profile 2021,” accessed October 15, 2024, https://health.ec.europa.eu/system/files/2021-12/2021_chp_hr_english.pdf. [↑](#endnote-ref-5)
5. Zoran Radosavljevic, “Croatian MEP: Public Health System Facing Big Cancer Crisis,” www.euractiv.com, May 3, 2023, https://www.euractiv.com/section/diabetes-cancer-hepatitis/news/croatian-mep-public-health-system-facing-big-cancer-crisis/. [↑](#endnote-ref-6)
6. “Croatia National Cancer Control Plan 2020 – 2030.” [↑](#endnote-ref-7)
7. Suzanne Wait et al., “Implementing Lung Cancer Screening in Europe: Taking a Systems Approach,” *JTO Clinical and Research Reports* 3, no. 5 (April 22, 2022): 100329, https://doi.org/10.1016/j.jtocrr.2022.100329. [↑](#endnote-ref-8)
8. “Lung Screening – from Clinical Studies to Established Programs,” accessed October 15, 2024, https://events.siemens-healthineers.com/sessions/symposium/update-on-lung-cancer-screening-in-europe. [↑](#endnote-ref-9)
9. “Croatia National Protocol - NACIONALNI PROGRAM PREVENCIJE RAKA PLUĆA.” [↑](#endnote-ref-10)
10. “Croatia National Protocol - NACIONALNI PROGRAM PREVENCIJE RAKA PLUĆA.” [↑](#endnote-ref-11)
11. “Croatia National Protocol - NACIONALNI PROGRAM PREVENCIJE RAKA PLUĆA.” [↑](#endnote-ref-12)
12. “Croatia National Protocol - NACIONALNI PROGRAM PREVENCIJE RAKA PLUĆA.” [↑](#endnote-ref-13)
13. Expert interview with Mr. Ivica Belina (Croatia). [↑](#endnote-ref-14)
14. “Lung Cancer Screening: 2022 Could Be a Turning Point for Europe | Cancerworld Magazine,” January 14, 2022, https://cancerworld.net/lung-cancer-screening-2022-could-be-a-turning-point-for-europe/. [↑](#endnote-ref-15)
15. “Croatia National Protocol - NACIONALNI PROGRAM PREVENCIJE RAKA PLUĆA.” [↑](#endnote-ref-16)
16. Expert interview with Mr. Ivica Belina (Croatia). [↑](#endnote-ref-17)
17. “New Lung Cancer Screening Roll out to Detect Cancer Sooner,” GOV.UK, n.d., https://www.gov.uk/government/news/new-lung-cancer-screening-roll-out-to-detect-cancer-sooner. [↑](#endnote-ref-18)
18. Expert interview with Dr. David Baldwin (England), Moderated by CRA, Personal communication. [↑](#endnote-ref-19)
19. Independent Investigation of the National Health Service in England [↑](#endnote-ref-20)
20. “The NHS Long Term Plan,” accessed October 15, 2024, https://www.longtermplan.nhs.uk/wp-content/uploads/2019/08/nhs-long-term-plan-version-1.2.pdf. [↑](#endnote-ref-21)
21. “New Lung Cancer Screening Roll out to Detect Cancer Sooner.” [↑](#endnote-ref-22)
22. “Lung Cancer - UK National Screening Committee (UK NSC) - GOV.UK,” accessed October 15, 2024, https://view-health-screening-recommendations.service.gov.uk/lung-cancer/. [↑](#endnote-ref-23)
23. “Standard Protocol Prepared for the Targeted Lung Health Checks Programme,” accessed October 15, 2024, https://www.england.nhs.uk/wp-content/uploads/2019/02/B1646-standard-protocol-targeted-lung-health-checks-programme-v2.pdf. [↑](#endnote-ref-24)
24. “New Lung Cancer Screening Roll out to Detect Cancer Sooner.” [↑](#endnote-ref-25)
25. Emma L O’Dowd et al., “Defining the Road Map to a UK National Lung Cancer Screening Programme,” *The Lancet Oncology* 24, no. 5 (May 2023): e207–18, https://doi.org/10.1016/S1470-2045(23)00104-3. [↑](#endnote-ref-26)
26. “New Lung Cancer Screening Roll out to Detect Cancer Sooner.” [↑](#endnote-ref-27)
27. “Health Funding Data Analysis,” The British Medical Association is the trade union and professional body for doctors in the UK., accessed October 15, 2024, https://www.bma.org.uk/advice-and-support/nhs-delivery-and-workforce/funding/health-funding-data-analysis. [↑](#endnote-ref-28)
28. Expert interview with Dr. Richard Booton (England), Moderated by CRA, Personal communication. [↑](#endnote-ref-29)
29. “Considerations to Ensure Optimum Roll-out of Targeted Lung Cancer Screening over the next Five Years” (British Society of Thoracic Imaging and The Royal College of Radiologists, n.d.), https://www.rcr.ac.uk/sites/default/files/final_pdf_considerations_to_ensure_optimum_roll-out_of_targeted_lung_cancer_screening.pdf. [↑](#endnote-ref-30)
30. Charlotte Wickens, “Why Do Diagnostics Matter?,” n.d. [↑](#endnote-ref-31)
31. Expert interview with Dr. Richard Booton (England). [↑](#endnote-ref-32)
32. “Manchester’s Lung Health Check Pilot,” accessed October 15, 2024, https://mft.nhs.uk/app/uploads/sites/12/2019/02/lung-health-check-manchester-report_tcm9-309848.pdf. [↑](#endnote-ref-33)
33. “Our Role in Lung Health Checks - Roy Castle Lung Cancer Foundation,” July 27, 2021, https://roycastle.org/lung-health-checks/our-role-in-lung-health-checks/. [↑](#endnote-ref-34)
34. “Lung Cancer - UK National Screening Committee (UK NSC) - GOV.UK.” [↑](#endnote-ref-35)
35. Dev Ops, “Lung Cancer Screening in Poland Pilot Program Is Restarted After COVID-19 Lockdown,” *ILCN.Org (ILCN/WCLC)* (blog), January 21, 2021, https://www.ilcn.org/lung-cancer-screening-in-poland-pilot-program-is-restarted-after-covid-19-lockdown/. [↑](#endnote-ref-36)
36. Henry Arnold, “Lung Cancer Screening in Poland,” The Lung Cancer Policy Network, November 28, 2022, https://www.lungcancerpolicynetwork.com/lung-cancer-screening-in-poland/. [↑](#endnote-ref-37)
37. “Narodowa Strategia Onkologiczna - Ministerstwo Zdrowia - Portal Gov.pl,” Ministerstwo Zdrowia, n.d., https://www.gov.pl/web/zdrowie/narodowa-strategia-onkologiczna-nso. [↑](#endnote-ref-38)
38. Arnold, “Lung Cancer Screening in Poland.” [↑](#endnote-ref-39)
39. “O projektcie,” Lung Check, accessed October 15, 2024, https://www.lungcheck.pl/o-projekcie. [↑](#endnote-ref-40)
40. “Narodowa Strategia Onkologiczna - Ministerstwo Zdrowia - Portal Gov.pl.” [↑](#endnote-ref-41)
41. “Poland Lung Cancer Screening Protocol - Ogólnopolski Program Wczesnego Wykrywania Raka Płuca (WWRP) Za Pomocą Niskodawkowej Tomografii Komputerowej (NDTK) – Połączenie Prewencji Wtórnej z Pierwotną w Celu Poprawy Świadomości Dotyczącej Raka Płuca Wśród Społeczeństwa i Personelu Ochrony Zdrowia,” accessed October 15, 2024, https://www.power.gov.pl/media/72320/Zalacznik_17_Ogolnopolski_Program_WWRP.pdf. [↑](#endnote-ref-42)
42. Ops, “Lung Cancer Screening in Poland Pilot Program Is Restarted After COVID-19 Lockdown.” [↑](#endnote-ref-43)
43. “Narodowa Strategia Onkologiczna - Ministerstwo Zdrowia - Portal Gov.pl.” [↑](#endnote-ref-44)
44. “Organizacja Programu,” Lung Check, accessed October 15, 2024, https://www.lungcheck.pl/rada-programowa. [↑](#endnote-ref-45)
45. Witold Rzyman et al., “Consensus Statement on a Screening Programme for the Detection of Early Lung Cancer in Poland,” *Advances in Respiratory Medicine* 86, no. 1 (2018): 53–74, https://doi.org/10.5603/ARM.2018.0009. [↑](#endnote-ref-46)
46. “Poland Lung Cancer Screening Protocol - Ogólnopolski Program Wczesnego Wykrywania Raka Płuca (WWRP) Za Pomocą Niskodawkowej Tomografii Komputerowej (NDTK) – Połączenie Prewencji Wtórnej z Pierwotną w Celu Poprawy Świadomości Dotyczącej Raka Płuca Wśród Społeczeństwa i Personelu Ochrony Zdrowia.” [↑](#endnote-ref-47)
47. “Poland Lung Cancer Screening Protocol - Ogólnopolski Program Wczesnego Wykrywania Raka Płuca (WWRP) Za Pomocą Niskodawkowej Tomografii Komputerowej (NDTK) – Połączenie Prewencji Wtórnej z Pierwotną w Celu Poprawy Świadomości Dotyczącej Raka Płuca Wśród Społeczeństwa i Personelu Ochrony Zdrowia.” [↑](#endnote-ref-48)
48. “Narodowa Strategia Onkologiczna - Ministerstwo Zdrowia - Portal Gov.pl.” [↑](#endnote-ref-49)
49. “Poland Lung Cancer Screening Protocol - Ogólnopolski Program Wczesnego Wykrywania Raka Płuca (WWRP) Za Pomocą Niskodawkowej Tomografii Komputerowej (NDTK) – Połączenie Prewencji Wtórnej z Pierwotną w Celu Poprawy Świadomości Dotyczącej Raka Płuca Wśród Społeczeństwa i Personelu Ochrony Zdrowia.” [↑](#endnote-ref-50)
50. “Szkolenia,” Lung Check, accessed October 15, 2024, https://www.lungcheck.pl/szkolenia. [↑](#endnote-ref-51)
51. “Szkolenia.” [↑](#endnote-ref-52)
52. Harry J. de Koning et al., “Reduced Lung-Cancer Mortality with Volume CT Screening in a Randomized Trial,” *New England Journal of Medicine* 382, no. 6 (February 6, 2020): 503–13, https://doi.org/10.1056/NEJMoa1911793. [↑](#endnote-ref-53)
53. Sebahat Ocak et al., “Lung Cancer in Belgium,” *Journal of Thoracic Oncology* 16, no. 10 (October 1, 2021): 1610–21, https://doi.org/10.1016/j.jtho.2021.07.022. [↑](#endnote-ref-54)
54. Annelin Marien and |Annelin Marien|, “Tegen 2026 willen longartsen screening naar longkanker bij Vlaamse bevolking: ‘Vroegtijdig opsporen zorgt voor kwart minder sterfgevallen,’” hln.be, August 18, 2022, https://www.hln.be/medisch/tegen-2026-willen-longartsen-screening-naar-longkanker-bij-vlaamse-bevolking-vroegtijdig-opsporen-zorgt-voor-kwart-minder-sterfgevallen~a8751d0e/. [↑](#endnote-ref-55)
55. Fabian Desimpel et al., *Lung Cancer Screening in a High-Risk Population*, 1st ed., KCE Reports - Health Technology Assessment (HTA) (BE: Belgian Health Care Knowledge Centre (KCE), 2024), https://doi.org/10.57598/R379C. [↑](#endnote-ref-56)
56. “Cancer,” SPF Santé publique, November 29, 2016, https://www.health.belgium.be/fr/cancer. [↑](#endnote-ref-57)
57. Expert interview with Dr. Annemiek Snoeckx (Belgium), Moderated by CRA, Personal communication. [↑](#endnote-ref-58)
58. Marien and Marien|, “Tegen 2026 willen longartsen screening naar longkanker bij Vlaamse bevolking.” [↑](#endnote-ref-59)
59. Desimpel et al., *Lung Cancer Screening in a High-Risk Population*. [↑](#endnote-ref-60)
60. Expert interview with Dr. Annemiek Snoeckx (Belgium). [↑](#endnote-ref-61)
61. Expert interview with Dr. Annemiek Snoeckx (Belgium). [↑](#endnote-ref-62)
62. University Hospital, Antwerp, “Feasibility Study of Lung Cancer Screening in the Flemish Region, the ZORALCS Study,” Clinical trial registration (clinicaltrials.gov, February 27, 2024), https://clinicaltrials.gov/study/NCT06293833. [↑](#endnote-ref-63)
63. Desimpel et al., *Lung Cancer Screening in a High-Risk Population*. [↑](#endnote-ref-64)
64. OECD, *EU Country Cancer Profile: Belgium 2023*, EU Country Cancer Profiles (OECD, 2023), https://doi.org/10.1787/9a976db3-en. [↑](#endnote-ref-65)
65. Desimpel et al., *Lung Cancer Screening in a High-Risk Population*. [↑](#endnote-ref-66)
66. Expert interview with Dr. Annemiek Snoeckx (Belgium). [↑](#endnote-ref-67)
67. Jens Vogel-Claussen et al., “Design and Rationale of the HANSE Study: A Holistic German Lung Cancer Screening Trial Using Low-Dose Computed Tomography,” *RöFo - Fortschritte auf dem Gebiet der Röntgenstrahlen und der bildgebenden Verfahren* 194, no. 12 (December 2022): 1333–45, https://doi.org/10.1055/a-1853-8291. [↑](#endnote-ref-68)
68. Bundesumweltministeriums, “Bundesumweltministerium lässt künftig Lungenkrebsfrüherkennung mittels Niedrigdosis-Computertomographie zu- BMUV - Pressemitteilung,” Bundesministerium für Umwelt, Naturschutz, nukleare Sicherheit und Verbraucherschutz, February 28, 2024, https://www.bmuv.de/PM11003. [↑](#endnote-ref-69)
69. Deutsche Röntgengesellschaft e.V, “Durchbruch Auf Dem Weg Zu Einem Früherkennungsprogramm Für Lungenkrebs | DRG.De,” n.d., https://www.drg.de. [↑](#endnote-ref-70)
70. “Initiation of the Consultation Procedure: Evaluation of Lung Cancer Early Detection Using Low-Dose Computed Tomography in Smokers - Federal Joint Committee,” accessed October 15, 2024, https://www.g-ba.de/beschluesse/6390/. [↑](#endnote-ref-71)
71. Expert interview with Mr. Sebastien Schmidt (Germany), Moderated by CRA, Personal communication. [↑](#endnote-ref-72)
72. Jens Vogel-Claussen et al., “Positionspapier zur Implementierung eines nationalen organisierten Programms in Deutschland zur Früherkennung von Lungenkrebs in Risikopopulationen mittels Low-dose-CT-Screening inklusive Management von abklärungsbedürftigen Screeningbefunden,” *RöFo - Fortschritte auf dem Gebiet der Röntgenstrahlen und der bildgebenden Verfahren* 196, no. 02 (February 2024): 134–53, https://doi.org/10.1055/a-2178-2846. [↑](#endnote-ref-73)
73. Dag Wormanns et al., “Joint Statement of the German Radiological Society and the German Respiratory Society on a Quality-Assured Early Detection Program for Lung Cancer with Low-Dose CT,” *RöFo - Fortschritte auf dem Gebiet der Röntgenstrahlen und der bildgebenden Verfahren* 191, no. 11 (November 2019): 993–97, https://doi.org/10.1055/a-0998-4399. [↑](#endnote-ref-74)
74. “[S19-02] Lung Cancer Screening Using Low-Dose Computed Tomography,” IQWiG, accessed October 15, 2024, https://www.iqwig.de/en/projects/s19-02.html. [↑](#endnote-ref-75)
75. Org_SG, “Lungenkrebsfrüherkennung mittels Niedrigdosis-Computertomographie - Wissenschaftliche Bewertung des Bundesamtes für Strahlenschutz gemäß § 84 Absatz 3 Strahlenschutzgesetz” (Bundesamt für Strahlenschutz (BfS), August 20, 2021), https://doris.bfs.de/jspui/handle/urn:nbn:de:0221-2021082028027. [↑](#endnote-ref-76)
76. Bundesumweltministeriums, “Referentenentwurf einer Verordnung über die Zulässigkeit der Anwendung der Niedrigdosis-Computertomographie zur Früherkennung von Lungenkrebs bei Rauchern (Lungenkrebs-Früherkennungs-Verordnung)- BMUV - Gesetze und Verordnungen,” bmuv.de, June 21, 2024, https://www.bmuv.de/GE1017. [↑](#endnote-ref-77)
77. Org_SG, “Lungenkrebsfrüherkennung mittels Niedrigdosis-Computertomographie - Wissenschaftliche Bewertung des Bundesamtes für Strahlenschutz gemäß § 84 Absatz 3 Strahlenschutzgesetz.” [↑](#endnote-ref-78)
78. Vogel-Claussen et al., “Design and Rationale of the HANSE Study.” [↑](#endnote-ref-79)
79. Bundesumweltministeriums, “Referentenentwurf einer Verordnung über die Zulässigkeit der Anwendung der Niedrigdosis-Computertomographie zur Früherkennung von Lungenkrebs bei Rauchern (Lungenkrebs-Früherkennungs-Verordnung)- BMUV - Gesetze und Verordnungen.” [↑](#endnote-ref-80)
80. Vogel-Claussen et al., “Positionspapier zur Implementierung eines nationalen organisierten Programms in Deutschland zur Früherkennung von Lungenkrebs in Risikopopulationen mittels Low-dose-CT-Screening inklusive Management von abklärungsbedürftigen Screeningbefunden.” [↑](#endnote-ref-81)
81. Org_SG, “Lungenkrebsfrüherkennung mittels Niedrigdosis-Computertomographie - Wissenschaftliche Bewertung des Bundesamtes für Strahlenschutz gemäß § 84 Absatz 3 Strahlenschutzgesetz.” [↑](#endnote-ref-82)
82. Vogel-Claussen et al., “Positionspapier zur Implementierung eines nationalen organisierten Programms in Deutschland zur Früherkennung von Lungenkrebs in Risikopopulationen mittels Low-dose-CT-Screening inklusive Management von abklärungsbedürftigen Screeningbefunden.” [↑](#endnote-ref-83)
83. Vogel-Claussen et al. [↑](#endnote-ref-84)
84. Mario Silva et al., “Low-Dose CT for Lung Cancer Screening: Position Paper from the Italian College of Thoracic Radiology,” *La Radiologia Medica* 127, no. 5 (May 1, 2022): 543–59, https://doi.org/10.1007/s11547-022-01471-y. [↑](#endnote-ref-85)
85. “MInistero Della Salute - Piano Oncologico Nazionale: Documento Di Pianificazione e Indirizzo per La Prevenzione e Il Contrasto Del Cancro 2023-2027,” accessed October 15, 2024, https://www.salute.gov.it/imgs/C_17_pubblicazioni_3291_allegato.pdf. [↑](#endnote-ref-86)
86. “RISP - Progetto Della Rete Italiana Screening-Polmonare.Pdf,” accessed October 15, 2024, https://www.myecole.it/ooc/wp-content/uploads/2021/02/Progetto-della-Rete-Italiana-Screening-Polmonare.pdf. [↑](#endnote-ref-87)
87. “Progetto Pilota Di Un Programma Di Screening per Il Tumore Polmonare Integrato Con La Cessazione Del Fumo: Percorsi, Selezione Dei Soggetti e Protocolli Diagnostici, in Vista Di Una Valutazione HTA | CCM - Network,” accessed January 15, 2025, https://www.ccm-network.it/progetto.jsp?id=node/2046&idP=740. [↑](#endnote-ref-88)
88. “Council Updates Its Recommendation to Screen for Cancer,” Consilium, accessed October 16, 2024, https://www.consilium.europa.eu/en/press/press-releases/2022/12/09/council-updates-its-recommendation-to-screen-for-cancer/. [↑](#endnote-ref-89)
89. “MInistero Della Salute - Piano Oncologico Nazionale: Documento Di Pianificazione e Indirizzo per La Prevenzione e Il Contrasto Del Cancro 2023-2027.” [↑](#endnote-ref-90)
90. “RISP - Progetto Della Rete Italiana Screening-Polmonare.Pdf.” [↑](#endnote-ref-91)
91. “Tumore al polmone, il programma di screening italiano fa scuola,” la Repubblica, November 22, 2023, https://www.repubblica.it/salute/2023/11/22/news/tumore_al_polmone_il_programma_di_screening_italiano_fa_scuola-421017269/. [↑](#endnote-ref-92)
92. Deanna, “ALCASE Italia,” ALCASE Italia, accessed October 16, 2024, https://alcase.it/. [↑](#endnote-ref-93)
93. “Rete italiana screening polmonare | cancro del polmone | alcase.eu,” accessed October 16, 2024, https://www.alcase.eu/home/rete-italiana-screening-polmonare/. [↑](#endnote-ref-94)
94. “Adapted HTA REPORT 2014 - Screening per Il Tumore Del Polmone.,” accessed October 16, 2024, https://www.salute.gov.it/imgs/C_17_ReportDispositivi_17_0_documentoITA.pdf. [↑](#endnote-ref-95)
95. “Workshop Screening Cancro Polmone Razionale,” accessed October 15, 2024, https://www.alcase.eu/wp-content/uploads/2019/01/WS_screening-cancro-polmone-RAZIONALE_14-diic2018.pdf. [↑](#endnote-ref-96)
96. “Adapted HTA REPORT 2014 - Screening per Il Tumore Del Polmone.” [↑](#endnote-ref-97)
97. “Adapted HTA REPORT 2014 - Screening per Il Tumore Del Polmone.” [↑](#endnote-ref-98)
98. “RISP - Progetto Della Rete Italiana Screening-Polmonare.Pdf.” [↑](#endnote-ref-99)
99. “Tumore al polmone, è tempo di pensare a uno screening nazionale,” la Repubblica, November 16, 2023, https://www.repubblica.it/salute/dossier/labrevolution/2023/11/16/news/tumore_polmone_screening_indagine_fumatori-420521803/. [↑](#endnote-ref-100)
100. Giulia Veronesi et al., “Favorable Incremental Cost-Effectiveness Ratio for Lung Cancer Screening in Italy,” *Lung Cancer* 143 (May 1, 2020): 73–79, https://doi.org/10.1016/j.lungcan.2020.03.015. [↑](#endnote-ref-101)
101. “RISP - Progetto Della Rete Italiana Screening-Polmonare.Pdf.” [↑](#endnote-ref-102)
102. Expert interview with Dr. Eugenio Paci (Italy), Moderated by CRA, Personal communication. [↑](#endnote-ref-103)
103. “Programma R.I.S.P. - Rete Italiana Screening Polmonare,” accessed October 16, 2024, https://programmarisp.it/. [↑](#endnote-ref-104)
104. “RISP - Progetto Della Rete Italiana Screening-Polmonare.Pdf.” [↑](#endnote-ref-105)
105. “Tumore al polmone, è tempo di pensare a uno screening nazionale.” [↑](#endnote-ref-106)
106. “RISP - Progetto Della Rete Italiana Screening-Polmonare.Pdf.” [↑](#endnote-ref-107)
107. “Lung Ambition Alliance,” accessed October 15, 2024, https://www.astrazeneca.es/areas-terapeuticas/oncologia/Lung_ambition_alliance.html. [↑](#endnote-ref-108)
108. “44th Conference – I-ELCAP,” accessed October 16, 2024, https://www.ielcap.org/home/ielcap/events/previous-meetings/44th-conference/. [↑](#endnote-ref-109)
109. Lidia Garcia Perez, “Evaluación del programa de cribado de cáncer de pulmón,” *Ministerio de Sanidad, Santa Cruz de Tenerife: Servicio Canario de la Salud, Santiago de Compostela: Agencia Gallega para la Gestión del Conocimiento en Salud, ACIS*, n.d. [↑](#endnote-ref-110)
110. Expert interview with Dr. Juan Carlos Trujillo (Spain), Moderated by CRA, Personal communication. [↑](#endnote-ref-111)
111. “Cribado Del Cáncer de Pulmón Mediante TC de Baja Dosis - Proyecto Piloto Nacional - Documento de Actuacion,” accessed October 15, 2024, https://sect.es/images/site/boletines/2022/enero/img/Documento_resumen.pdf. [↑](#endnote-ref-112)
112. “44th Conference – I-ELCAP.” [↑](#endnote-ref-113)
113. “Ministerio de Sanidad - Áreas - Cartera de Servicios Complementaria de Las Comunidades Autónomas y de Las Mutualidades de Funcionarios,” accessed October 15, 2024, https://www.sanidad.gob.es/profesionales/prestacionesSanitarias/CarteraDeServicios/ComplemenariaCS/CS-Complementaria.htm. [↑](#endnote-ref-114)
114. “Galicia seleccionará con inteligencia artificial a los candidatos a un cribado de cáncer de colon y próstata,” La Voz de Galicia, October 14, 2023, https://www.lavozdegalicia.es/noticia/sociedad/2023/10/14/galicia-aplicara-inteligencia-artificial-cribados-cancer-pulmon/0003_202310G14P24991.htm. [↑](#endnote-ref-115)
115. Comunidad de Madrid, “Díaz Ayuso anuncia un programa piloto propio de cribado de cáncer de pulmón para incluirlo como prestación de cartera básica de servicios,” Comunidad de Madrid, September 25, 2023, https://www.comunidad.madrid/noticias/2023/09/25/diaz-ayuso-anuncia-programa-piloto-propio-cribado-cancer-pulmon-incluirlo-prestacion-cartera-basica-servicios. [↑](#endnote-ref-116)
116. “Cribado Del Cáncer de Pulmón Mediante TC de Baja Dosis - Proyecto Piloto Nacional - Documento de Actuacion.” [↑](#endnote-ref-117)
117. “Lung Ambition Alliance,” accessed October 16, 2024, https://www.astrazeneca.es/areas-terapeuticas/oncologia/Lung_ambition_alliance.html. [↑](#endnote-ref-118)
118. AEACAP, “AEACaP exige soluciones para afrontar el cáncer de pulmón como la nueva epidemia oculta,” AEACAP - Asociación Afectados Cáncer de Pulmón, May 25, 2023, https://afectadoscancerdepulmon.com/aeacap-exige-soluciones-para-afrontar-el-cancer-de-pulmon-como-la-nueva-epidemia-oculta/. [↑](#endnote-ref-119)
119. “¿Por Qué No Se Implanta El Cribado de Cáncer de Pulmón En España? | Salud,” accessed October 15, 2024, https://www.elmundo.es/ciencia-y-salud/salud/2023/09/22/650c773921efa0b9398b457c.html. [↑](#endnote-ref-120)
120. “Cribado Del Cáncer de Pulmón Mediante TC de Baja Dosis - Proyecto Piloto Nacional - Documento de Actuacion.” [↑](#endnote-ref-121)
121. Expert interview with Dr. Juan Carlos Trujillo (Spain). [↑](#endnote-ref-122)
122. “Cribado Del Cáncer de Pulmón Mediante TC de Baja Dosis - Proyecto Piloto Nacional - Documento de Actuacion.” [↑](#endnote-ref-123)
123. “Cribado Del Cáncer de Pulmón Mediante TC de Baja Dosis - Proyecto Piloto Nacional - Documento de Actuacion.” [↑](#endnote-ref-124)
124. Expert interview with Dr. Juan Carlos Trujillo (Spain). [↑](#endnote-ref-125)
125. Garcia Perez, “Evaluación del programa de cribado de cáncer de pulmón.” [↑](#endnote-ref-126)
126. AEACAP, “AEACaP participa en el lanzamiento de Alia , una plataforma digital al servicio de pacientes y profesionales de cáncer de pulmón,” AEACAP - Asociación Afectados Cáncer de Pulmón, February 3, 2023, https://afectadoscancerdepulmon.com/aeacap-participa-en-el-lanzamiento-de-alia-una-plataforma-digital-al-servicio-de-pacientes-y-profesionales-de-cancer-de-pulmon/. [↑](#endnote-ref-127)
127. “Screening - RCC,” accessed October 15, 2024, https://www.cancercentrum.se/samverkan/regional-cancer-centres/screening/. [↑](#endnote-ref-128)
128. Katrin Trysell, “Socialstyrelsen: För tidigt att ta ställning till screening,” *Läkartidningen* (blog), May 11, 2022, https://lakartidningen.se/aktuellt/nyheter/2022/05/socialstyrelsen-for-tidigt-att-ta-stallning/. [↑](#endnote-ref-129)
129. Katrin Trysell, “Lungcancerscreening: Snart Går Startskottet i Stockholm,” *Läkartidningen* (blog), May 11, 2022, https://lakartidningen.se/aktuellt/nyheter/2022/05/lungcancerscreening-snart-gar-startskottet-i-stockholm/. [↑](#endnote-ref-130)
130. 4 Mar 2024, “Mef Nilbert Leads the Update of a New Swedish Cancer Strategy | LUCC,” August 12, 2024, https://www.lucc.lu.se/article/mef-nilbert-leads-update-new-swedish-cancer-strategy. [↑](#endnote-ref-131)
131. Expert interview with Ms. Ebba Hallersjö (Sweden), Moderated by CRA, Personal communication. [↑](#endnote-ref-132)
132. “Lung Cancer Screening - RCC Knowledge Bank,” accessed October 15, 2024, https://kunskapsbanken.cancercentrum.se/diagnoser/lungcancer/vardprogram/lungcancerscreening/. [↑](#endnote-ref-133)
133. “Lung Cancer Screening - Current Situation in Sweden - Lung & Allergy Forum,” accessed October 15, 2024, https://etidning.slmf.se/p/lung-allergiforum/nr-4-2022-12-08/a/lungcancerscreening-aktuellt-lage-i-sverige/1915/815853/34423657. [↑](#endnote-ref-134)
134. “Svenska Lungcancerstudiegruppens insamlingsstiftelse,” *Svenska Lungcancerstudiegruppen* (blog), accessed October 16, 2024, https://slusg.org/om-oss/. [↑](#endnote-ref-135)
135. “Lungcancerscreening - RCC Kunskapsbanken,” n.d., https://kunskapsbanken.cancercentrum.se/diagnoser/lungcancer/vardprogram/lungcancerscreening/. [↑](#endnote-ref-136)
136. “Lungcancerscreening - RCC Kunskapsbanken.” [↑](#endnote-ref-137)
137. Expert interview with Ms. Ebba Hallersjö (Sweden). [↑](#endnote-ref-138)
138. Olivia Wigzell, “Nationella screeningprogram,” *Socialstyrelsen*, n.d., https://www.socialstyrelsen.se/globalassets/sharepoint-dokument/artikelkatalog/nationella-screeningprogram/2019-4-12.pdf. [↑](#endnote-ref-139)
139. “Lung Cancer Screening - RCC Knowledge Bank.” [↑](#endnote-ref-140)
140. Wigzell, “Nationella screeningprogram.” [↑](#endnote-ref-141)
141. “Breathing a New Era: A Comparative Analysis of Lung Cancer Policies across Europe,” accessed October 15, 2024, https://www.eiu.com/graphics/marketing/pdf/Lung-Cancer%20in-Europe-EIU-2019-9-10-final.pdf. [↑](#endnote-ref-142)
142. Trysell, “Lungcancerscreening.” [↑](#endnote-ref-143)
143. Wait et al., “Implementing Lung Cancer Screening in Europe.” [↑](#endnote-ref-144)
144. “La Stratégie Décennale de Lutte Contre Les Cancers 2021-2030 - Stratégie de Lutte Contre Les Cancers En France,” accessed October 15, 2024, https://www.e-cancer.fr/Institut-national-du-cancer/Strategie-de-lutte-contre-les-cancers-en-France/La-strategie-decennale-de-lutte-contre-les-cancers-2021-2030. [↑](#endnote-ref-145)
145. “Dépistage du cancer bronchopulmonaire par scanner thoracique faible dose sans injection : actualisation de l’avis de 2016,” Haute Autorité de Santé, n.d., https://www.has-sante.fr/jcms/p_3310940/fr/depistage-du-cancer-bronchopulmonaire-par-scanner-thoracique-faible-dose-sans-injection-actualisation-de-l-avis-de-2016. [↑](#endnote-ref-146)
146. “La Stratégie Décennale de Lutte Contre Les Cancers 2021-2030 - Stratégie de Lutte Contre Les Cancers En France.” [↑](#endnote-ref-147)
147. “Une nouvelle stratégie nationale pour faire reculer le cancer en France,” February 4, 2021, https://www.lemonde.fr/planete/article/2021/02/04/une-nouvelle-strategie-nationale-pour-faire-reculer-le-cancer_6068728_3244.html. [↑](#endnote-ref-148)
148. Mathieu Lederlin et al., “Lung Cancer Screening: French Radiologists Should Prepare for It,” *Diagnostic and Interventional Imaging* 102, no. 4 (April 1, 2021): 197–98, https://doi.org/10.1016/j.diii.2021.02.004. [↑](#endnote-ref-149)
149. Expert interview with Dr. Sebastien Couraud (France), Moderated by CRA, Personal communication. [↑](#endnote-ref-150)
150. “Dépistage du cancer bronchopulmonaire par scanner thoracique faible dose sans injection.” [↑](#endnote-ref-151)
151. “Dépistage du cancer bronchopulmonaire par scanner thoracique faible dose sans injection.” [↑](#endnote-ref-152)
152. *04/07/2022 - Webinaire “Programme Pilote Dépistage Des Cancers Du Poumon Pour Fumeurs et Ex-Fumeurs,”* 2022, https://www.youtube.com/watch?v=LWaBVaNCFIE. [↑](#endnote-ref-153)
153. Expert interview with Dr. Sebastien Couraud (France). [↑](#endnote-ref-154)
154. “Dépistage du cancer bronchopulmonaire par scanner thoracique faible dose sans injection.” [↑](#endnote-ref-155)
155. “Dépistage Du Cancer Du Poumon : Lancement d’un Projet Pilote Par l’Institut - Actualités,” accessed October 16, 2024, https://www.e-cancer.fr/Actualites-et-evenements/Actualites/Depistage-du-cancer-du-poumon-lancement-d-un-projet-pilote-par-l-Institut. [↑](#endnote-ref-156)
156. Feng, “A Modeling Study of Eligibility Criteria for National Lung Cancer Screening in France,” n.d. [↑](#endnote-ref-157)
157. “Dépistage Du Cancer Du Poumon : Lancement d’un Projet Pilote Par l’Institut - Actualités.” [↑](#endnote-ref-158)
158. “Une nouvelle stratégie nationale pour faire reculer le cancer en France.” [↑](#endnote-ref-159)
159. “Dépistage du cancer bronchopulmonaire par scanner thoracique faible dose sans injection.” [↑](#endnote-ref-160)
160. “Le Mois sans Tabac Ravive Les Besoins de Scanner Low Dose Pour Le Dépistage Précoce Du Cancer Du Poumon,” accessed October 16, 2024, https://www.thema-radiologie.fr/actualites/2815/le-mois-sans-tabac-ravive-les-besoins-de-scanner-low-dose-pour-le-depistage-precoce-du-cancer-du-poumon.html. [↑](#endnote-ref-161)
161. “Les Sociétés Savantes Se Mobilisent à Nouveau En Faveur Du Dépistage Du Cancer Du Poumon Par Scanner Low Dose,” accessed October 16, 2024, https://www.thema-radiologie.fr/actualites/3131/les-societes-savantes-se-mobilisent-a-nouveau-en-faveur-du-depistage-du-cancer-du-poumon-par-scanner-low-dose.html. [↑](#endnote-ref-162)
162. Lederlin et al., “Lung Cancer Screening.” [↑](#endnote-ref-163)
163. “Le Scanner Low Dose Recommandé Par Le Collectif Ensemble Nous Poumons,” accessed October 16, 2024, https://www.thema-radiologie.fr/actualites/3096/le-scanner-low-dose-recommande-par-le-collectif-ensemble-nous-poumons.html. [↑](#endnote-ref-164)
164. “Metropolitan Hospital: Το Πρόγραμμα «Πνευμόνων Υγεία» Που Σώζει Ζωές,” Το site για την καλή Υγεία, την σωστή Διατροφή και την Ευεξία - ygeiamou.gr, December 1, 2022, https://www.ygeiamou.gr/επιστημονικές-εξελίξεις/309190/metropolitan-hospital-to-programma-pnevmonon-igia-pou-sozi-zoes/. [↑](#endnote-ref-165)
165. “Greece Lung Cancer Screening Medical Societies Recommendations - ΠΡΟΤΕΙΝΟΜΕΝΗ ΔΙΑΔΙΚΑΣΙΑ ΠΡΟΣΥΜΠΤΩΜΑΤΙΚΟΥ ΕΛΕΓΧΟΥ ΚΑΡΚΙΝΟΥ ΠΝΕΥΜΟΝΑ (ΠΕΚΠ),” accessed October 15, 2024, https://ellok.org/wp-content/uploads/2023/11/%CE%A0%CE%A1%CE%9F%CE%A4%CE%95%CE%99%CE%9D%CE%9F%CE%9C%CE%95%CE%9D%CE%97-%CE%94%CE%99%CE%91%CE%94%CE%99%CE%9A%CE%91%CE%A3%CE%99%CE%91-%CE%A0%CE%95%CE%9A%CE%A0-19.04.2023.pdf. [↑](#endnote-ref-166)
166. “Πρόγραμμα ‘Σπύρος Δοξιάδης’: Ποιοι Πολίτες Δικαιούνται Δωρεάν Προληπτικές Εξετάσεις Τους Επόμενους Μήνες,” Iatropedia, January 22, 2020, https://www.iatropedia.gr/eidiseis/programma-spyros-doksiadis-poioi-polites-dikaiountai-dorean-proliptikes-eksetaseis-tous-epomenous-mines/126246/. [↑](#endnote-ref-167)
167. webteam, “Ενημερωτικό σημείωμα για τη σύσκεψη υπό τον Πρωθυπουργό Κυριάκο Μητσοτάκη με αντικείμενο τις προληπτικές εξετάσεις και την πρωτοβάθμια φροντίδα | Ο Πρωθυπουργός της Ελληνικής Δημοκρατίας,” April 20, 2023, https://www.primeminister.gr/2023/04/20/31715. [↑](#endnote-ref-168)
168. “Δωρεάν Προληπτικές Εξετάσεις: Ξεκινούν Από Το Νέο Έτος Για Τον Καρκίνο Πνεύμονα Σε 4 Νοσοκομεία,” *HealthReport.Gr* (blog), accessed November 11, 2024, https://www.healthreport.gr/dorean-proliptikes-exetaseis-xekinoyn-apo-to-neo-etos-gia-ton-karkino-pneymona-se-4-nosokomeia/. [↑](#endnote-ref-169)
169. “Πλεύρης: «Όχημα» για την καταπολέμηση του καρκίνου τα προγράμματα προληπτικού ελέγχου – Τα μεγάλα στοιχήματα,” Insider, January 23, 2023, https://www.insider.gr/politiki/260266/karkinos-ohima-gia-tin-katapolemisi-toy-ta-programmata-proliptikoy-eleghoy-ta. [↑](#endnote-ref-170)
170. “Greece National Cancer Plan - ΕΘΝΙΚΟ ΣΧΕΔΙΟ ΔΡΑΣΗΣ ΓΙΑ ΤΟΝ ΚΑΡΚΙΝΟ 2011-2015,” accessed October 16, 2024, https://www.anti-cancer.gr/catalogue/SxedioDrasis_24selido.pdf. [↑](#endnote-ref-171)
171. “Εθνικό Σχέδιο Δράσης για την πρόληψη και την αντιμετώπιση του καρκίνου,” Υπουργείο Υγείας, accessed October 16, 2024, https://www.moh.gov.gr/articles/ministry/grafeio-typoy/press-releases/11810-ethniko-sxedio-drashs-gia-thn-prolhpsh-kai-thn-antimetwpish-toy-karkinoy. [↑](#endnote-ref-172)
172. “Πλεύρης.” [↑](#endnote-ref-173)
173. “Greece Lung Cancer Screening Medical Societies Recommendations - ΠΡΟΤΕΙΝΟΜΕΝΗ ΔΙΑΔΙΚΑΣΙΑ ΠΡΟΣΥΜΠΤΩΜΑΤΙΚΟΥ ΕΛΕΓΧΟΥ ΚΑΡΚΙΝΟΥ ΠΝΕΥΜΟΝΑ (ΠΕΚΠ).” [↑](#endnote-ref-174)
174. Kyriakos Souliotis et al., “Lung Cancer Screening in Greece: A Modelling Study to Estimate the Impact on Lung Cancer Life Years,” *Cancers* 14, no. 22 (November 8, 2022): 5484, https://doi.org/10.3390/cancers14225484. [↑](#endnote-ref-175)
175. “Lung Cancer: 25% Mortality Reduction by Screening Implementation FairLife LCC’s Online Conference: «Lung Cancer – Prevention and Early Diagnosis: Lung Cancer Screening Programme in Greece, Are We Ready? »,” *FairLife L.C.C.* (blog), November 25, 2021, https://fairlifelcc.com/en/lung-cancer-25-mortality-reduction-by-screening-implementation-fairlife-lccs-online-conference-lung-cancer-prevention-and-early-diagnosis-lung-cancer-screening-programme-2/. [↑](#endnote-ref-176)
176. Nikolaos Vrachnis and Nikolaos Vlachadis, “Guidelines on Cervical and Breast Cancer Screening in Greece,” *The Lancet* 385, no. 9970 (February 28, 2015): 772, https://doi.org/10.1016/S0140-6736(15)60434-2. [↑](#endnote-ref-177)
177. “ESMO Congress 2022 | OncologyPRO,” accessed October 16, 2024, https://oncologypro.esmo.org/meeting-resources/esmo-congress-2022/a-pilot-study-of-a-lung-cancer-screening-program-with-low-dose-computed-tomography-in-high-risk-individuals-in-greece. [↑](#endnote-ref-178)
178. “Metropolitan Hospital.” [↑](#endnote-ref-179)
179. “Ασημένια διάκριση για το Πρόγραμμα Προληπτικού Ελέγχου «Πνευμόνων Υγεία» για τον καρκίνο του πνεύμονα στο Metropolitan Hospital,” ProtoThema, July 7, 2022, https://www.protothema.gr/ugeia/article/1262344/asimenia-diakrisi-programma-proliptikou-eleghou-pneumonon-ugeia-karkino-tou-pneumona-sto-metropolitan-hospital/. [↑](#endnote-ref-180)
180. “Greece Lung Cancer Screening Medical Societies Recommendations - ΠΡΟΤΕΙΝΟΜΕΝΗ ΔΙΑΔΙΚΑΣΙΑ ΠΡΟΣΥΜΠΤΩΜΑΤΙΚΟΥ ΕΛΕΓΧΟΥ ΚΑΡΚΙΝΟΥ ΠΝΕΥΜΟΝΑ (ΠΕΚΠ).” [↑](#endnote-ref-181)
181. “Greece Lung Cancer Screening Medical Societies Recommendations - ΠΡΟΤΕΙΝΟΜΕΝΗ ΔΙΑΔΙΚΑΣΙΑ ΠΡΟΣΥΜΠΤΩΜΑΤΙΚΟΥ ΕΛΕΓΧΟΥ ΚΑΡΚΙΝΟΥ ΠΝΕΥΜΟΝΑ (ΠΕΚΠ).” [↑](#endnote-ref-182)
182. Marianthi Pelekanaki, “Greek Cancer Plan to Be Tabled Soon amid Concerning Mortality Rates,” www.euractiv.com, December 18, 2023, https://www.euractiv.com/section/health-consumers/news/greek-cancer-plan-to-be-tabled-soon-amid-concerning-mortality-rates/. [↑](#endnote-ref-183)
183. “Greece Lung Cancer Screening Medical Societies Recommendations - ΠΡΟΤΕΙΝΟΜΕΝΗ ΔΙΑΔΙΚΑΣΙΑ ΠΡΟΣΥΜΠΤΩΜΑΤΙΚΟΥ ΕΛΕΓΧΟΥ ΚΑΡΚΙΝΟΥ ΠΝΕΥΜΟΝΑ (ΠΕΚΠ).” [↑](#endnote-ref-184)
184. “Our Vision,” *FairLife L.C.C.* (blog), accessed October 16, 2024, https://fairlifelcc.com/en/about/our-vision/. [↑](#endnote-ref-185)
185. “Πρόγραμμα - Ο Καρκίνος του Πνεύμονα σε Πρώτο Πλάνο,” *FairLife L.C.C.* (blog), accessed October 16, 2024, https://fairlifelcc.com/o-karkinos-toy-pneymona-se-proto-plano/programma/. [↑](#endnote-ref-186)
186. “Καρκίνος Πνεύμονα: Η Κοινωνική Ανισότητα Εμπόδιο Στην Αντιμετώπιση Των Ασθενών,” Το site για την καλή Υγεία, την σωστή Διατροφή και την Ευεξία - ygeiamou.gr, November 28, 2023, https://www.ygeiamou.gr/ειδήσεις/361984/karkinos-pnevmona-i-kinoniki-anisotita-empodio-stin-antimetopisi-ton-asthenon/.

     **Bibliography**

     *04/07/2022 - Webinaire “Programme Pilote Dépistage Des Cancers Du Poumon Pour Fumeurs et Ex-Fumeurs,”* 2022. https://www.youtube.com/watch?v=LWaBVaNCFIE.

     “44th Conference – I-ELCAP.” Accessed October 16, 2024. https://www.ielcap.org/home/ielcap/events/previous-meetings/44th-conference/.

     “Adapted HTA REPORT 2014 - Screening per Il Tumore Del Polmone.” Accessed October 16, 2024. https://www.salute.gov.it/imgs/C_17_ReportDispositivi_17_0_documentoITA.pdf.

     AEACAP. “AEACaP exige soluciones para afrontar el cáncer de pulmón como la nueva epidemia oculta.” AEACAP - Asociación Afectados Cáncer de Pulmón, May 25, 2023. https://afectadoscancerdepulmon.com/aeacap-exige-soluciones-para-afrontar-el-cancer-de-pulmon-como-la-nueva-epidemia-oculta/.

     ———. “AEACaP participa en el lanzamiento de Alia , una plataforma digital al servicio de pacientes y profesionales de cáncer de pulmón.” AEACAP - Asociación Afectados Cáncer de Pulmón, February 3, 2023. https://afectadoscancerdepulmon.com/aeacap-participa-en-el-lanzamiento-de-alia-una-plataforma-digital-al-servicio-de-pacientes-y-profesionales-de-cancer-de-pulmon/.

     Arnold, Henry. “Lung Cancer Screening in Poland.” The Lung Cancer Policy Network, November 28, 2022. https://www.lungcancerpolicynetwork.com/lung-cancer-screening-in-poland/.

     “Breathing a New Era: A Comparative Analysis of Lung Cancer Policies across Europe.” Accessed October 15, 2024. https://www.eiu.com/graphics/marketing/pdf/Lung-Cancer%20in-Europe-EIU-2019-9-10-final.pdf.

     Bundesumweltministeriums. “Bundesumweltministerium lässt künftig Lungenkrebsfrüherkennung mittels Niedrigdosis-Computertomographie zu- BMUV - Pressemitteilung.” Bundesministerium für Umwelt, Naturschutz, nukleare Sicherheit und Verbraucherschutz, February 28, 2024. https://www.bmuv.de/PM11003.

     ———. “Referentenentwurf einer Verordnung über die Zulässigkeit der Anwendung der Niedrigdosis-Computertomographie zur Früherkennung von Lungenkrebs bei Rauchern (Lungenkrebs-Früherkennungs-Verordnung)- BMUV - Gesetze und Verordnungen.” bmuv.de, June 21, 2024. https://www.bmuv.de/GE1017.

     “Considerations to Ensure Optimum Roll-out of Targeted Lung Cancer Screening over the next Five Years.” British Society of Thoracic Imaging and The Royal College of Radiologists, n.d. https://www.rcr.ac.uk/sites/default/files/final_pdf_considerations_to_ensure_optimum_roll-out_of_targeted_lung_cancer_screening.pdf.

     Consilium. “Council Updates Its Recommendation to Screen for Cancer.” Accessed October 16, 2024. https://www.consilium.europa.eu/en/press/press-releases/2022/12/09/council-updates-its-recommendation-to-screen-for-cancer/.

     “Cribado Del Cáncer de Pulmón Mediante TC de Baja Dosis - Proyecto Piloto Nacional - Documento de Actuacion.” Accessed October 15, 2024. https://sect.es/images/site/boletines/2022/enero/img/Documento_resumen.pdf.

     “Croatia National Cancer Control Plan 2020 – 2030.” Accessed October 15, 2024. https://www.iccp-portal.org/system/files/plans/NPPR_ENG_final.pdf.

     “Croatia National Protocol - NACIONALNI PROGRAM PREVENCIJE RAKA PLUĆA.” Accessed October 15, 2024. https://zdravlje.gov.hr/UserDocsImages/2019%20Programi%20i%20projekti/NACIONALNI%20PROGRAM%20PREVENCIJE%20RAKA%20PLU%C4%86A.pdf.

     Deanna. “ALCASE Italia.” ALCASE Italia. Accessed October 16, 2024. https://alcase.it/.

     “Dépistage Du Cancer Du Poumon : Lancement d’un Projet Pilote Par l’Institut - Actualités.” Accessed October 16, 2024. https://www.e-cancer.fr/Actualites-et-evenements/Actualites/Depistage-du-cancer-du-poumon-lancement-d-un-projet-pilote-par-l-Institut.

     Desimpel, Fabian, Janis Luyten, Cécile Camberlin, Célia Primus-de Jong, Leen Verleye, and Mattias Neyt. *Lung Cancer Screening in a High-Risk Population*. 1st ed. KCE Reports - Health Technology Assessment (HTA). BE: Belgian Health Care Knowledge Centre (KCE), 2024. https://doi.org/10.57598/R379C.

     “ESMO Congress 2022 | OncologyPRO.” Accessed October 16, 2024. https://oncologypro.esmo.org/meeting-resources/esmo-congress-2022/a-pilot-study-of-a-lung-cancer-screening-program-with-low-dose-computed-tomography-in-high-risk-individuals-in-greece.

     e.V, Deutsche Röntgengesellschaft. “Durchbruch Auf Dem Weg Zu Einem Früherkennungsprogramm Für Lungenkrebs | DRG.De,” n.d. https://www.drg.de.

     Expert interview with Dr. Annemiek Snoeckx (Belgium). Moderated by CRA, Personal communication.

     Expert interview with Dr. David Baldwin (England). Moderated by CRA, Personal communication.

     Expert interview with Dr. Eugenio Paci (Italy). Moderated by CRA, Personal communication.

     Expert interview with Dr. Juan Carlos Trujillo (Spain). Moderated by CRA, Personal communication.

     Expert interview with Dr. Richard Booton (England). Moderated by CRA, Personal communication.

     Expert interview with Dr. Sebastien Couraud (France). Moderated by CRA, Personal communication.

     Expert interview with Mr. Ivica Belina (Croatia). Moderated by CRA, Personal communication.

     Expert interview with Mr. Sebastien Schmidt (Germany). Moderated by CRA, Personal communication.

     Expert interview with Ms. Ebba Hallersjö (Sweden). Moderated by CRA, Personal communication.

     FairLife L.C.C. “Lung Cancer: 25% Mortality Reduction by Screening Implementation FairLife LCC’s Online Conference: «Lung Cancer – Prevention and Early Diagnosis: Lung Cancer Screening Programme in Greece, Are We Ready? »,” November 25, 2021. https://fairlifelcc.com/en/lung-cancer-25-mortality-reduction-by-screening-implementation-fairlife-lccs-online-conference-lung-cancer-prevention-and-early-diagnosis-lung-cancer-screening-programme-2/.

     FairLife L.C.C. “Our Vision.” Accessed October 16, 2024. https://fairlifelcc.com/en/about/our-vision/.

     FairLife L.C.C. “Πρόγραμμα - Ο Καρκίνος του Πνεύμονα σε Πρώτο Πλάνο.” Accessed October 16, 2024. https://fairlifelcc.com/o-karkinos-toy-pneymona-se-proto-plano/programma/.

     Feng. “A Modeling Study of Eligibility Criteria for National Lung Cancer Screening in France,” n.d.

     Garcia Perez, Lidia. “Evaluación del programa de cribado de cáncer de pulmón.” *Ministerio de Sanidad, Santa Cruz de Tenerife: Servicio Canario de la Salud, Santiago de Compostela: Agencia Gallega para la Gestión del Conocimiento en Salud, ACIS*, n.d.

     GOV.UK. “New Lung Cancer Screening Roll out to Detect Cancer Sooner,” n.d. https://www.gov.uk/government/news/new-lung-cancer-screening-roll-out-to-detect-cancer-sooner.

     “Greece Lung Cancer Screening Medical Societies Recommendations - ΠΡΟΤΕΙΝΟΜΕΝΗ ΔΙΑΔΙΚΑΣΙΑ ΠΡΟΣΥΜΠΤΩΜΑΤΙΚΟΥ ΕΛΕΓΧΟΥ ΚΑΡΚΙΝΟΥ ΠΝΕΥΜΟΝΑ (ΠΕΚΠ).” Accessed October 15, 2024. https://ellok.org/wp-content/uploads/2023/11/%CE%A0%CE%A1%CE%9F%CE%A4%CE%95%CE%99%CE%9D%CE%9F%CE%9C%CE%95%CE%9D%CE%97-%CE%94%CE%99%CE%91%CE%94%CE%99%CE%9A%CE%91%CE%A3%CE%99%CE%91-%CE%A0%CE%95%CE%9A%CE%A0-19.04.2023.pdf.

     “Greece National Cancer Plan - ΕΘΝΙΚΟ ΣΧΕΔΙΟ ΔΡΑΣΗΣ ΓΙΑ ΤΟΝ ΚΑΡΚΙΝΟ 2011-2015.” Accessed October 16, 2024. https://www.anti-cancer.gr/catalogue/SxedioDrasis_24selido.pdf.

     Haute Autorité de Santé. “Dépistage du cancer bronchopulmonaire par scanner thoracique faible dose sans injection : actualisation de l’avis de 2016,” n.d. https://www.has-sante.fr/jcms/p_3310940/fr/depistage-du-cancer-bronchopulmonaire-par-scanner-thoracique-faible-dose-sans-injection-actualisation-de-l-avis-de-2016.

     HealthReport.gr. “Δωρεάν Προληπτικές Εξετάσεις: Ξεκινούν Από Το Νέο Έτος Για Τον Καρκίνο Πνεύμονα Σε 4 Νοσοκομεία.” Accessed November 11, 2024. https://www.healthreport.gr/dorean-proliptikes-exetaseis-xekinoyn-apo-to-neo-etos-gia-ton-karkino-pneymona-se-4-nosokomeia/.

     Iatropedia. “Πρόγραμμα ‘Σπύρος Δοξιάδης’: Ποιοι Πολίτες Δικαιούνται Δωρεάν Προληπτικές Εξετάσεις Τους Επόμενους Μήνες,” January 22, 2020. https://www.iatropedia.gr/eidiseis/programma-spyros-doksiadis-poioi-polites-dikaiountai-dorean-proliptikes-eksetaseis-tous-epomenous-mines/126246/.

     “Initiation of the Consultation Procedure: Evaluation of Lung Cancer Early Detection Using Low-Dose Computed Tomography in Smokers - Federal Joint Committee.” Accessed October 15, 2024. https://www.g-ba.de/beschluesse/6390/.

     Insider. “Πλεύρης: «Όχημα» για την καταπολέμηση του καρκίνου τα προγράμματα προληπτικού ελέγχου – Τα μεγάλα στοιχήματα,” January 23, 2023. https://www.insider.gr/politiki/260266/karkinos-ohima-gia-tin-katapolemisi-toy-ta-programmata-proliptikoy-eleghoy-ta.

     IQWiG. “[S19-02] Lung Cancer Screening Using Low-Dose Computed Tomography.” Accessed October 15, 2024. https://www.iqwig.de/en/projects/s19-02.html.

     Koning, Harry J. de, Carlijn M. van der Aalst, Pim A. de Jong, Ernst T. Scholten, Kristiaan Nackaerts, Marjolein A. Heuvelmans, Jan-Willem J. Lammers, et al. “Reduced Lung-Cancer Mortality with Volume CT Screening in a Randomized Trial.” *New England Journal of Medicine* 382, no. 6 (February 6, 2020): 503–13. https://doi.org/10.1056/NEJMoa1911793.

     la Repubblica. “Tumore al polmone, è tempo di pensare a uno screening nazionale,” November 16, 2023. https://www.repubblica.it/salute/dossier/labrevolution/2023/11/16/news/tumore_polmone_screening_indagine_fumatori-420521803/.

     la Repubblica. “Tumore al polmone, il programma di screening italiano fa scuola,” November 22, 2023. https://www.repubblica.it/salute/2023/11/22/news/tumore_al_polmone_il_programma_di_screening_italiano_fa_scuola-421017269/.

     “La Stratégie Décennale de Lutte Contre Les Cancers 2021-2030 - Stratégie de Lutte Contre Les Cancers En France.” Accessed October 15, 2024. https://www.e-cancer.fr/Institut-national-du-cancer/Strategie-de-lutte-contre-les-cancers-en-France/La-strategie-decennale-de-lutte-contre-les-cancers-2021-2030.

     La Voz de Galicia. “Galicia seleccionará con inteligencia artificial a los candidatos a un cribado de cáncer de colon y próstata,” October 14, 2023. https://www.lavozdegalicia.es/noticia/sociedad/2023/10/14/galicia-aplicara-inteligencia-artificial-cribados-cancer-pulmon/0003_202310G14P24991.htm.

     “Le Mois sans Tabac Ravive Les Besoins de Scanner Low Dose Pour Le Dépistage Précoce Du Cancer Du Poumon.” Accessed October 16, 2024. https://www.thema-radiologie.fr/actualites/2815/le-mois-sans-tabac-ravive-les-besoins-de-scanner-low-dose-pour-le-depistage-precoce-du-cancer-du-poumon.html.

     “Le Scanner Low Dose Recommandé Par Le Collectif Ensemble Nous Poumons.” Accessed October 16, 2024. https://www.thema-radiologie.fr/actualites/3096/le-scanner-low-dose-recommande-par-le-collectif-ensemble-nous-poumons.html.

     Lederlin, Mathieu, Constance de Margerie-Mellon, Samia Boussouar, Sébastien Bommart, and Caroline Caramella. “Lung Cancer Screening: French Radiologists Should Prepare for It.” *Diagnostic and Interventional Imaging* 102, no. 4 (April 1, 2021): 197–98. https://doi.org/10.1016/j.diii.2021.02.004.

     “Les Sociétés Savantes Se Mobilisent à Nouveau En Faveur Du Dépistage Du Cancer Du Poumon Par Scanner Low Dose.” Accessed October 16, 2024. https://www.thema-radiologie.fr/actualites/3131/les-societes-savantes-se-mobilisent-a-nouveau-en-faveur-du-depistage-du-cancer-du-poumon-par-scanner-low-dose.html.

     “Lung Ambition Alliance.” Accessed October 15, 2024. https://www.astrazeneca.es/areas-terapeuticas/oncologia/Lung_ambition_alliance.html.

     “Lung Ambition Alliance.” Accessed October 16, 2024. https://www.astrazeneca.es/areas-terapeuticas/oncologia/Lung_ambition_alliance.html.

     “Lung Cancer - UK National Screening Committee (UK NSC) - GOV.UK.” Accessed October 15, 2024. https://view-health-screening-recommendations.service.gov.uk/lung-cancer/.

     “Lung Cancer Screening - Current Situation in Sweden - Lung & Allergy Forum.” Accessed October 15, 2024. https://etidning.slmf.se/p/lung-allergiforum/nr-4-2022-12-08/a/lungcancerscreening-aktuellt-lage-i-sverige/1915/815853/34423657.

     “Lung Cancer Screening - RCC Knowledge Bank.” Accessed October 15, 2024. https://kunskapsbanken.cancercentrum.se/diagnoser/lungcancer/vardprogram/lungcancerscreening/.

     “Lung Cancer Screening: 2022 Could Be a Turning Point for Europe | Cancerworld Magazine,” January 14, 2022. https://cancerworld.net/lung-cancer-screening-2022-could-be-a-turning-point-for-europe/.

     Lung Check. “O projektcie.” Accessed October 15, 2024. https://www.lungcheck.pl/o-projekcie.

     Lung Check. “Organizacja Programu.” Accessed October 15, 2024. https://www.lungcheck.pl/rada-programowa.

     Lung Check. “Szkolenia.” Accessed October 15, 2024. https://www.lungcheck.pl/szkolenia.

     “Lung Screening – from Clinical Studies to Established Programs.” Accessed October 15, 2024. https://events.siemens-healthineers.com/sessions/symposium/update-on-lung-cancer-screening-in-europe.

     “Lungcancerscreening - RCC Kunskapsbanken,” n.d. https://kunskapsbanken.cancercentrum.se/diagnoser/lungcancer/vardprogram/lungcancerscreening/.

     Madrid, Comunidad de. “Díaz Ayuso anuncia un programa piloto propio de cribado de cáncer de pulmón para incluirlo como prestación de cartera básica de servicios.” Comunidad de Madrid, September 25, 2023. https://www.comunidad.madrid/noticias/2023/09/25/diaz-ayuso-anuncia-programa-piloto-propio-cribado-cancer-pulmon-incluirlo-prestacion-cartera-basica-servicios.

     “Manchester’s Lung Health Check Pilot.” Accessed October 15, 2024. https://mft.nhs.uk/app/uploads/sites/12/2019/02/lung-health-check-manchester-report_tcm9-309848.pdf.

     Mar 2024, 4. “Mef Nilbert Leads the Update of a New Swedish Cancer Strategy | LUCC,” August 12, 2024. https://www.lucc.lu.se/article/mef-nilbert-leads-update-new-swedish-cancer-strategy.

     Marien, Annelin, and |Annelin Marien|. “Tegen 2026 willen longartsen screening naar longkanker bij Vlaamse bevolking: ‘Vroegtijdig opsporen zorgt voor kwart minder sterfgevallen.’” hln.be, August 18, 2022. https://www.hln.be/medisch/tegen-2026-willen-longartsen-screening-naar-longkanker-bij-vlaamse-bevolking-vroegtijdig-opsporen-zorgt-voor-kwart-minder-sterfgevallen~a8751d0e/.

     “Ministerio de Sanidad - Áreas - Cartera de Servicios Complementaria de Las Comunidades Autónomas y de Las Mutualidades de Funcionarios.” Accessed October 15, 2024. https://www.sanidad.gob.es/profesionales/prestacionesSanitarias/CarteraDeServicios/ComplemenariaCS/CS-Complementaria.htm.

     “Ministero Della Salute - Piano Oncologico Nazionale: Documento Di Pianificazione e Indirizzo per La Prevenzione e Il Contrasto Del Cancro 2023-2027.” Accessed October 15, 2024. https://www.salute.gov.it/imgs/C_17_pubblicazioni_3291_allegato.pdf.

     Ministerstwo Zdrowia. “Narodowa Strategia Onkologiczna - Ministerstwo Zdrowia - Portal Gov.pl,” n.d. https://www.gov.pl/web/zdrowie/narodowa-strategia-onkologiczna-nso.

     Ocak, Sebahat, Kurt Tournoy, Thierry Berghmans, Ingel Demedts, Rodolphe Durieux, Annelies Janssens, Luigi Moretti, et al. “Lung Cancer in Belgium.” *Journal of Thoracic Oncology* 16, no. 10 (October 1, 2021): 1610–21. https://doi.org/10.1016/j.jtho.2021.07.022.

     O’Dowd, Emma L, Richard W Lee, Ahsan R Akram, Emily C Bartlett, Stephen H Bradley, Kate Brain, Matthew E J Callister, et al. “Defining the Road Map to a UK National Lung Cancer Screening Programme.” *The Lancet Oncology* 24, no. 5 (May 2023): e207–18. https://doi.org/10.1016/S1470-2045(23)00104-3.

     OECD. *EU Country Cancer Profile: Belgium 2023*. EU Country Cancer Profiles. OECD, 2023. https://doi.org/10.1787/9a976db3-en.

     Ops, Dev. “Lung Cancer Screening in Poland Pilot Program Is Restarted After COVID-19 Lockdown.” *ILCN.Org (ILCN/WCLC)* (blog), January 21, 2021. https://www.ilcn.org/lung-cancer-screening-in-poland-pilot-program-is-restarted-after-covid-19-lockdown/.

     Org_SG. “Lungenkrebsfrüherkennung mittels Niedrigdosis-Computertomographie - Wissenschaftliche Bewertung des Bundesamtes für Strahlenschutz gemäß § 84 Absatz 3 Strahlenschutzgesetz.” Bundesamt für Strahlenschutz (BfS), August 20, 2021. https://doris.bfs.de/jspui/handle/urn:nbn:de:0221-2021082028027.

     “Our Role in Lung Health Checks - Roy Castle Lung Cancer Foundation,” July 27, 2021. https://roycastle.org/lung-health-checks/our-role-in-lung-health-checks/.

     Pelekanaki, Marianthi. “Greek Cancer Plan to Be Tabled Soon amid Concerning Mortality Rates.” www.euractiv.com, December 18, 2023. https://www.euractiv.com/section/health-consumers/news/greek-cancer-plan-to-be-tabled-soon-amid-concerning-mortality-rates/.

     “Poland Lung Cancer Screening Protocol - Ogólnopolski Program Wczesnego Wykrywania Raka Płuca (WWRP) Za Pomocą Niskodawkowej Tomografii Komputerowej (NDTK) – Połączenie Prewencji Wtórnej z Pierwotną w Celu Poprawy Świadomości Dotyczącej Raka Płuca Wśród Społeczeństwa i Personelu Ochrony Zdrowia.” Accessed October 15, 2024. https://www.power.gov.pl/media/72320/Zalacznik_17_Ogolnopolski_Program_WWRP.pdf.

     “¿Por Qué No Se Implanta El Cribado de Cáncer de Pulmón En España? | Salud.” Accessed October 15, 2024. https://www.elmundo.es/ciencia-y-salud/salud/2023/09/22/650c773921efa0b9398b457c.html.

     “Progetto Pilota Di Un Programma Di Screening per Il Tumore Polmonare Integrato Con La Cessazione Del Fumo: Percorsi, Selezione Dei Soggetti e Protocolli Diagnostici, in Vista Di Una Valutazione HTA | CCM - Network.” Accessed January 15, 2025. https://www.ccm-network.it/progetto.jsp?id=node/2046&idP=740.

     “Programma R.I.S.P. - Rete Italiana Screening Polmonare.” Accessed October 16, 2024. https://programmarisp.it/.

     ProtoThema. “Ασημένια διάκριση για το Πρόγραμμα Προληπτικού Ελέγχου «Πνευμόνων Υγεία» για τον καρκίνο του πνεύμονα στο Metropolitan Hospital,” July 7, 2022. https://www.protothema.gr/ugeia/article/1262344/asimenia-diakrisi-programma-proliptikou-eleghou-pneumonon-ugeia-karkino-tou-pneumona-sto-metropolitan-hospital/.

     Radosavljevic, Zoran. “Croatian MEP: Public Health System Facing Big Cancer Crisis.” www.euractiv.com, May 3, 2023. https://www.euractiv.com/section/diabetes-cancer-hepatitis/news/croatian-mep-public-health-system-facing-big-cancer-crisis/.

     “Rete italiana screening polmonare | cancro del polmone | alcase.eu.” Accessed October 16, 2024. https://www.alcase.eu/home/rete-italiana-screening-polmonare/.

     “RISP - Progetto Della Rete Italiana Screening-Polmonare.Pdf.” Accessed October 15, 2024. https://www.myecole.it/ooc/wp-content/uploads/2021/02/Progetto-della-Rete-Italiana-Screening-Polmonare.pdf.

     Rzyman, Witold, Joanna Didkowska, Robert Dziedzic, Tomasz Grodzki, Tadeusz Orłowski, Edyta Szurowska, Renata Langfort, et al. “Consensus Statement on a Screening Programme for the Detection of Early Lung Cancer in Poland.” *Advances in Respiratory Medicine* 86, no. 1 (2018): 53–74. https://doi.org/10.5603/ARM.2018.0009.

     “Screening - RCC.” Accessed October 15, 2024. https://www.cancercentrum.se/samverkan/regional-cancer-centres/screening/.

     Silva, Mario, Giulia Picozzi, Nicola Sverzellati, Sandra Anglesio, Maurizio Bartolucci, Edoardo Cavigli, Annalisa Deliperi, et al. “Low-Dose CT for Lung Cancer Screening: Position Paper from the Italian College of Thoracic Radiology.” *La Radiologia Medica* 127, no. 5 (May 1, 2022): 543–59. https://doi.org/10.1007/s11547-022-01471-y.

     Souliotis, Kyriakos, Christina Golna, Pavlos Golnas, Ioannis-Anestis Markakis, Helena Linardou, Dimitra Sifaki-Pistolla, and Evi Hatziandreou. “Lung Cancer Screening in Greece: A Modelling Study to Estimate the Impact on Lung Cancer Life Years.” *Cancers* 14, no. 22 (November 8, 2022): 5484. https://doi.org/10.3390/cancers14225484.

     SPF Santé publique. “Cancer,” November 29, 2016. https://www.health.belgium.be/fr/cancer.

     “Standard Protocol Prepared for the Targeted Lung Health Checks Programme.” Accessed October 15, 2024. https://www.england.nhs.uk/wp-content/uploads/2019/02/B1646-standard-protocol-targeted-lung-health-checks-programme-v2.pdf.

     “State of Health in the EU Croatia Country Health Profile 2021.” Accessed October 15, 2024. https://health.ec.europa.eu/system/files/2021-12/2021_chp_hr_english.pdf.

     Svenska Lungcancerstudiegruppen. “Svenska Lungcancerstudiegruppens insamlingsstiftelse.” Accessed October 16, 2024. https://slusg.org/om-oss/.

     The British Medical Association is the trade union and professional body for doctors in the UK. “Health Funding Data Analysis.” Accessed October 15, 2024. https://www.bma.org.uk/advice-and-support/nhs-delivery-and-workforce/funding/health-funding-data-analysis.

     “The NHS Long Term Plan.” Accessed October 15, 2024. https://www.longtermplan.nhs.uk/wp-content/uploads/2019/08/nhs-long-term-plan-version-1.2.pdf.

     Trysell, Katrin. “Lungcancerscreening: Snart Går Startskottet i Stockholm.” *Läkartidningen* (blog), May 11, 2022. https://lakartidningen.se/aktuellt/nyheter/2022/05/lungcancerscreening-snart-gar-startskottet-i-stockholm/.

     ———. “Socialstyrelsen: För tidigt att ta ställning till screening.” *Läkartidningen* (blog), May 11, 2022. https://lakartidningen.se/aktuellt/nyheter/2022/05/socialstyrelsen-for-tidigt-att-ta-stallning/.

     “Une nouvelle stratégie nationale pour faire reculer le cancer en France.” February 4, 2021. https://www.lemonde.fr/planete/article/2021/02/04/une-nouvelle-strategie-nationale-pour-faire-reculer-le-cancer_6068728_3244.html.

     University Hospital, Antwerp. “Feasibility Study of Lung Cancer Screening in the Flemish Region, the ZORALCS Study.” Clinical trial registration. clinicaltrials.gov, February 27, 2024. https://clinicaltrials.gov/study/NCT06293833.

     Veronesi, Giulia, Niccolò Navone, Pierluigi Novellis, Elisa Dieci, Luca Toschi, Laura Velutti, Michela Solinas, Elena Vanni, Marco Alloisio, and Simone Ghislandi. “Favorable Incremental Cost-Effectiveness Ratio for Lung Cancer Screening in Italy.” *Lung Cancer* 143 (May 1, 2020): 73–79. https://doi.org/10.1016/j.lungcan.2020.03.015.

     Vogel-Claussen, Jens, Torsten Gerriet Blum, Stefan Andreas, Torsten T. Bauer, Jörg Barkhausen, Volker Harth, Hans-Ulrich Kauczor, et al. “Positionspapier zur Implementierung eines nationalen organisierten Programms in Deutschland zur Früherkennung von Lungenkrebs in Risikopopulationen mittels Low-dose-CT-Screening inklusive Management von abklärungsbedürftigen Screeningbefunden.” *RöFo - Fortschritte auf dem Gebiet der Röntgenstrahlen und der bildgebenden Verfahren* 196, no. 02 (February 2024): 134–53. https://doi.org/10.1055/a-2178-2846.

     Vogel-Claussen, Jens, Florian Lasch, Benjamin-Alexander Bollmann, Katharina May, Alexander Kuhlmann, Gerald Schmid-Bindert, Rudolf Kaaks, Jörg Barkhausen, Sabine Bohnet, and Martin Reck. “Design and Rationale of the HANSE Study: A Holistic German Lung Cancer Screening Trial Using Low-Dose Computed Tomography.” *RöFo - Fortschritte auf dem Gebiet der Röntgenstrahlen und der bildgebenden Verfahren* 194, no. 12 (December 2022): 1333–45. https://doi.org/10.1055/a-1853-8291.

     Vrachnis, Nikolaos, and Nikolaos Vlachadis. “Guidelines on Cervical and Breast Cancer Screening in Greece.” *The Lancet* 385, no. 9970 (February 28, 2015): 772. https://doi.org/10.1016/S0140-6736(15)60434-2.

     Wait, Suzanne, Arturo Alvarez-Rosete, Tasnime Osama, Dani Bancroft, Robin Cornelissen, Ante Marušić, Pilar Garrido, et al. “Implementing Lung Cancer Screening in Europe: Taking a Systems Approach.” *JTO Clinical and Research Reports* 3, no. 5 (April 22, 2022): 100329. https://doi.org/10.1016/j.jtocrr.2022.100329.

     webteam. “Ενημερωτικό σημείωμα για τη σύσκεψη υπό τον Πρωθυπουργό Κυριάκο Μητσοτάκη με αντικείμενο τις προληπτικές εξετάσεις και την πρωτοβάθμια φροντίδα | Ο Πρωθυπουργός της Ελληνικής Δημοκρατίας,” April 20, 2023. https://www.primeminister.gr/2023/04/20/31715.

     Wickens, Charlotte. “Why Do Diagnostics Matter?,” n.d.

     Wigzell, Olivia. “Nationella screeningprogram.” *Socialstyrelsen*, n.d. https://www.socialstyrelsen.se/globalassets/sharepoint-dokument/artikelkatalog/nationella-screeningprogram/2019-4-12.pdf.

     “Workshop Screening Cancro Polmone Razionale.” Accessed October 15, 2024. https://www.alcase.eu/wp-content/uploads/2019/01/WS_screening-cancro-polmone-RAZIONALE_14-diic2018.pdf.

     Wormanns, Dag, Hans-Ulrich Kauczor, Gerald Antoch, Jürgen Biederer, Felix J. F. Herth, Jens Vogel-Claussen, Niels Reinmuth, Michael Pfeifer, Board of Directors, Deutsche Röntgengesellschaft, and Board of Directors, Deutsche Gesellschaft für Pneumologie und Beatmungsmedizin. “Joint Statement of the German Radiological Society and the German Respiratory Society on a Quality-Assured Early Detection Program for Lung Cancer with Low-Dose CT.” *RöFo - Fortschritte auf dem Gebiet der Röntgenstrahlen und der bildgebenden Verfahren* 191, no. 11 (November 2019): 993–97. https://doi.org/10.1055/a-0998-4399.

     Το site για την καλή Υγεία, την σωστή Διατροφή και την Ευεξία - ygeiamou.gr. “Metropolitan Hospital: Το Πρόγραμμα «Πνευμόνων Υγεία» Που Σώζει Ζωές,” December 1, 2022. https://www.ygeiamou.gr/επιστημονικές-εξελίξεις/309190/metropolitan-hospital-to-programma-pnevmonon-igia-pou-sozi-zoes/.

     Το site για την καλή Υγεία, την σωστή Διατροφή και την Ευεξία - ygeiamou.gr. “Καρκίνος Πνεύμονα: Η Κοινωνική Ανισότητα Εμπόδιο Στην Αντιμετώπιση Των Ασθενών,” November 28, 2023. https://www.ygeiamou.gr/ειδήσεις/361984/karkinos-pnevmona-i-kinoniki-anisotita-empodio-stin-antimetopisi-ton-asthenon/.

     Υπουργείο Υγείας. “Εθνικό Σχέδιο Δράσης για την πρόληψη και την αντιμετώπιση του καρκίνου.” Accessed October 16, 2024. https://www.moh.gov.gr/articles/ministry/grafeio-typoy/press-releases/11810-ethniko-sxedio-drashs-gia-thn-prolhpsh-kai-thn-antimetwpish-toy-karkinoy. [↑](#endnote-ref-187)
