# Supplementary material for: From aspiration to reality: a cross-country analysis of the implementation of lung cancer screening in Europe
Source: Front Public Health. 2026 Apr 1;14:1766560. doi: 10.3389/fpubh.2026.1766560 (PMC13081776; doi:10.3389/fpubh.2026.1766560)
Supplement: Supplementary file 2 [file Data_Sheet_2.docx]

From aspiration to reality: A cross-country analysis of the implementation of lung cancer screening in Europe

Supplementary Material B: Visual summary assessment for each country across 5 dimensions of the framework

Manuscript for submission to Frontiers in Public Health

Article type: Policy and Practice Reviews

2026

# Appendix – Summary B: Visual overview of country profiles

## Croatia

Figure 5. Timeline of key events in the implementation of a national LCS program in Croatia


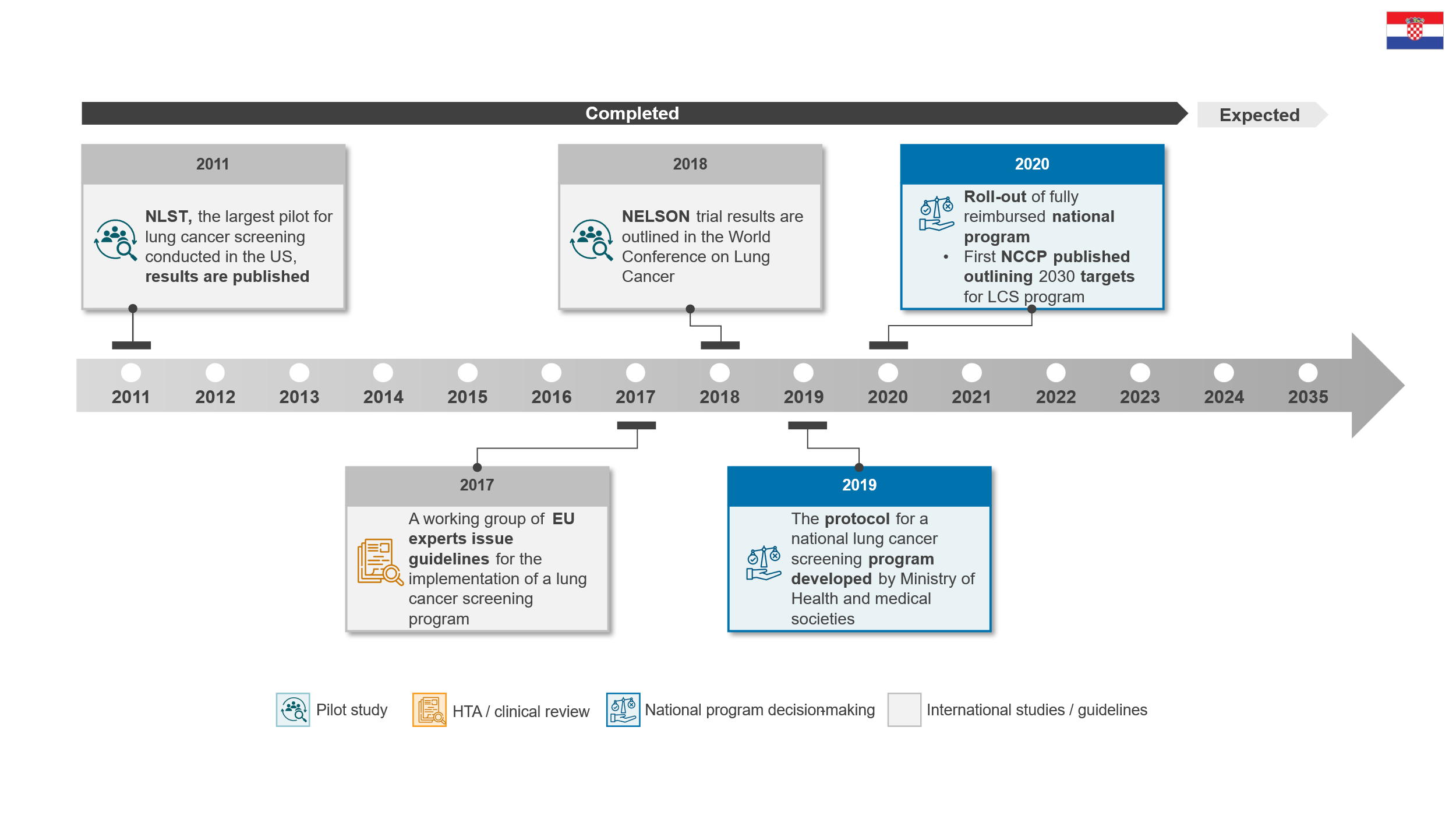


Figure 6a. Assessment for Croatia for policy prioritisation & governance and clinical review dimensions


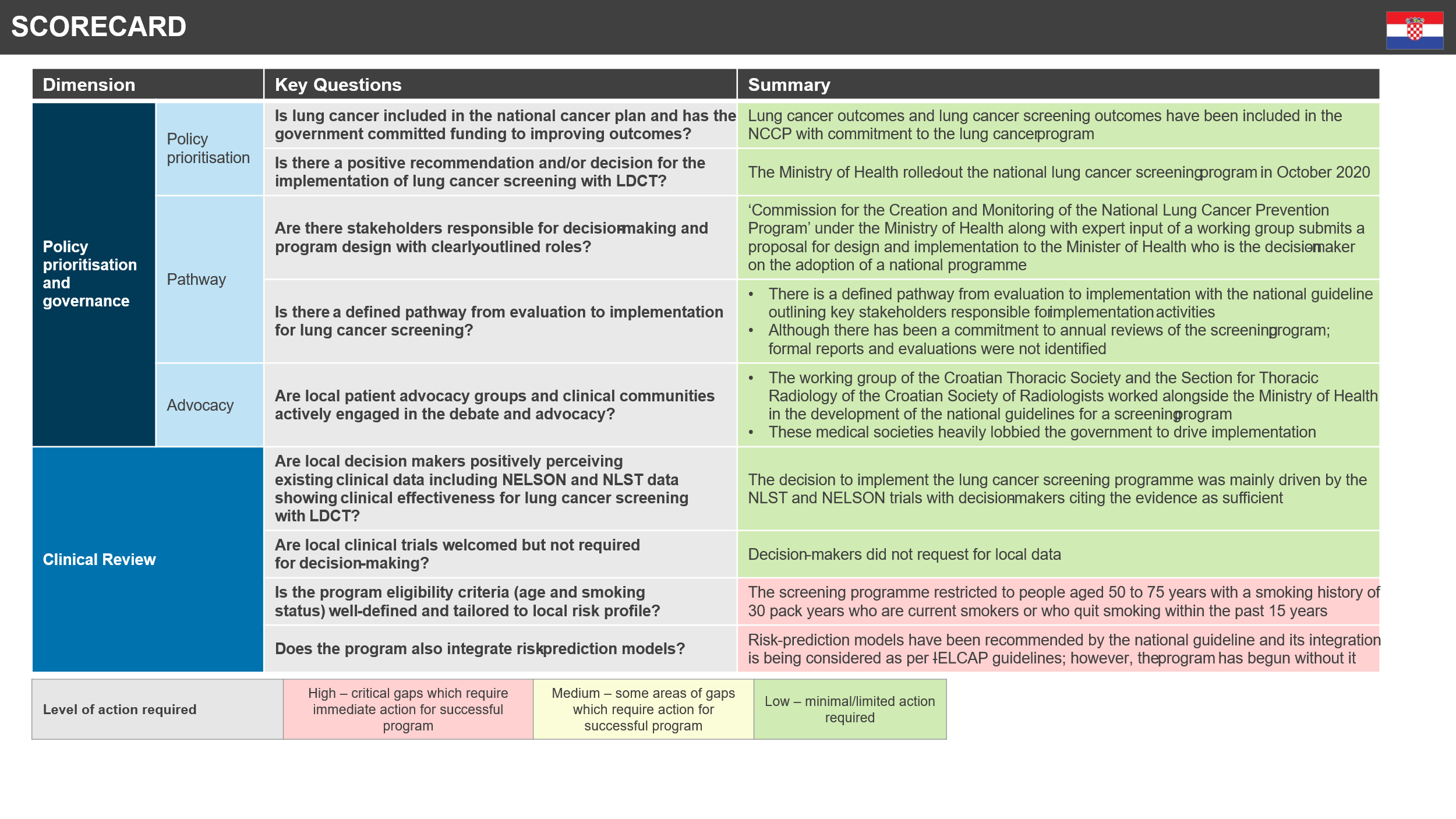


Figure 6b. Assessment for Croatia for program design dimension


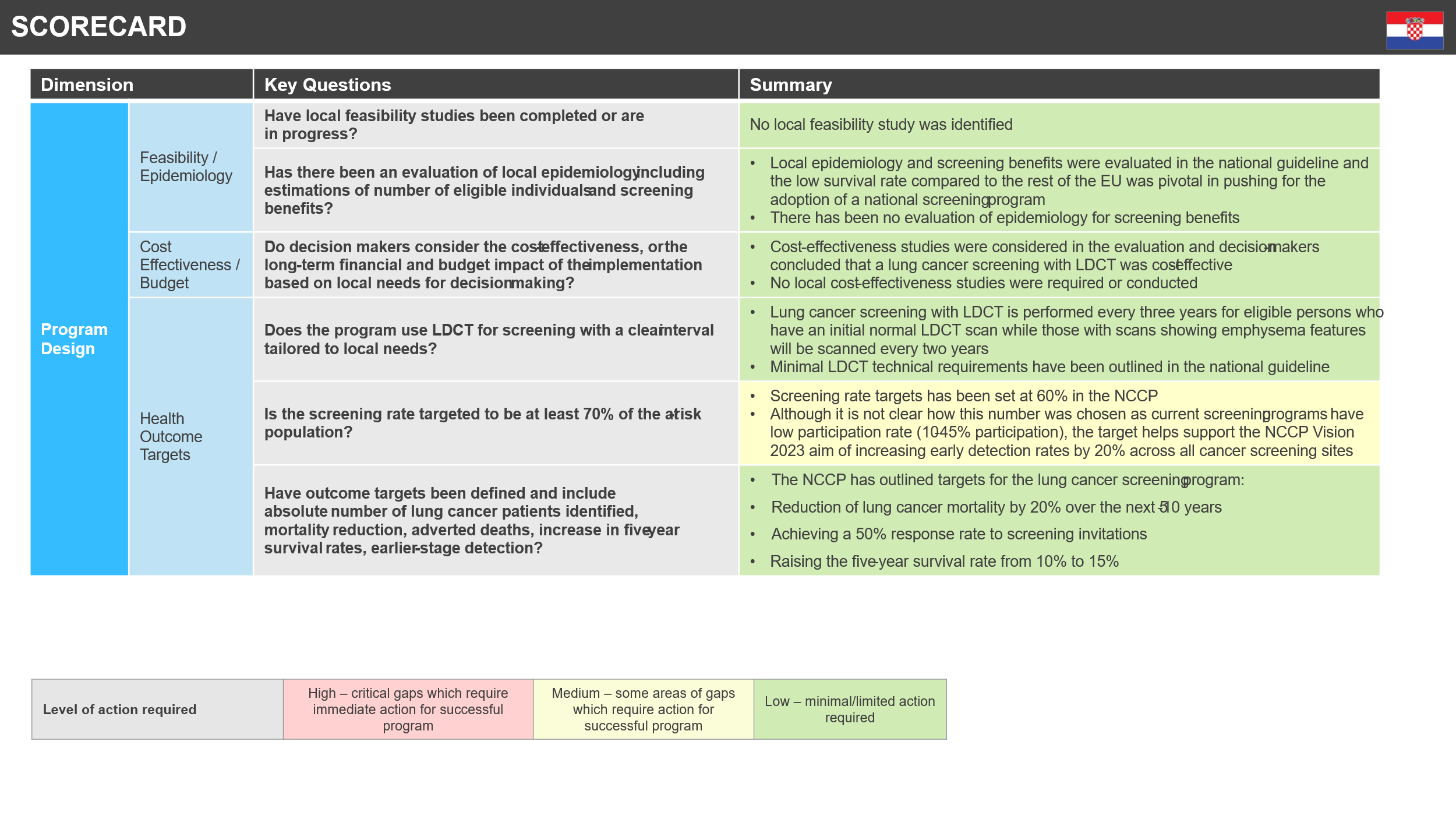


Figure 6c. Assessment for Croatia for implementation & awareness dimensions


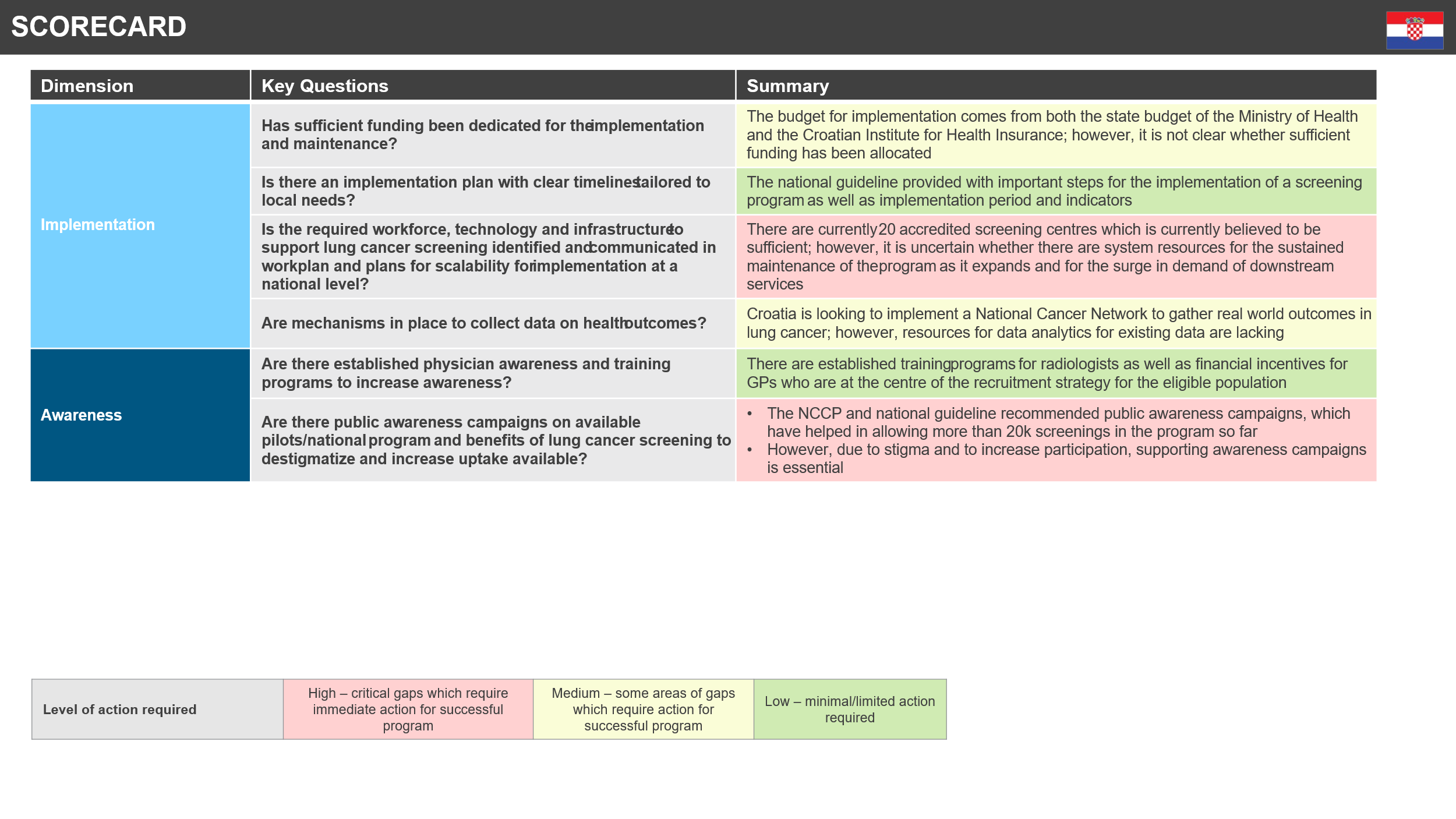


## England

Figure 7. Timeline of key events in the implementation of a national LCS program in England


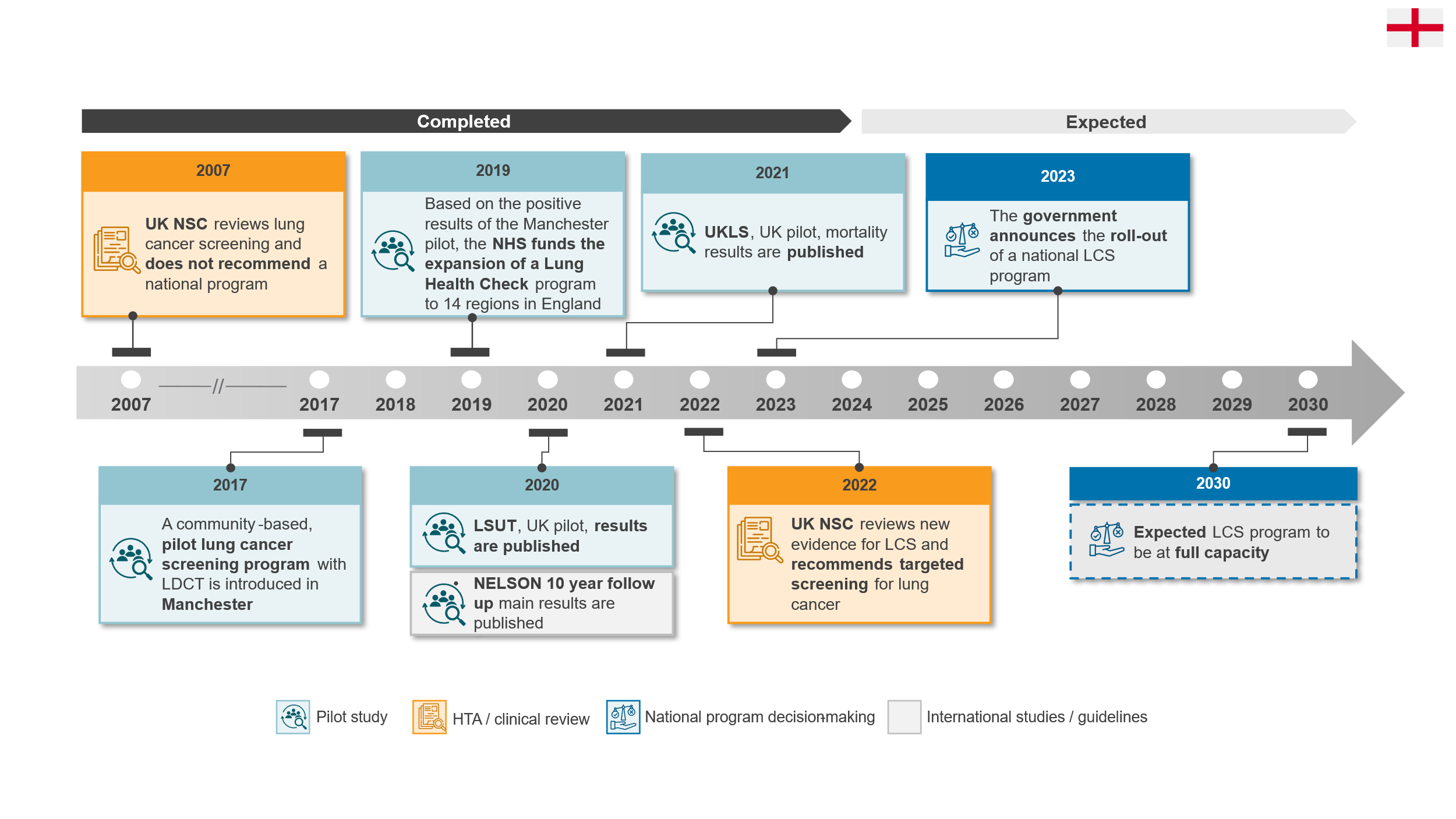


Figure 8a. Assessment for England for policy prioritisation & governance and clinical review dimensions


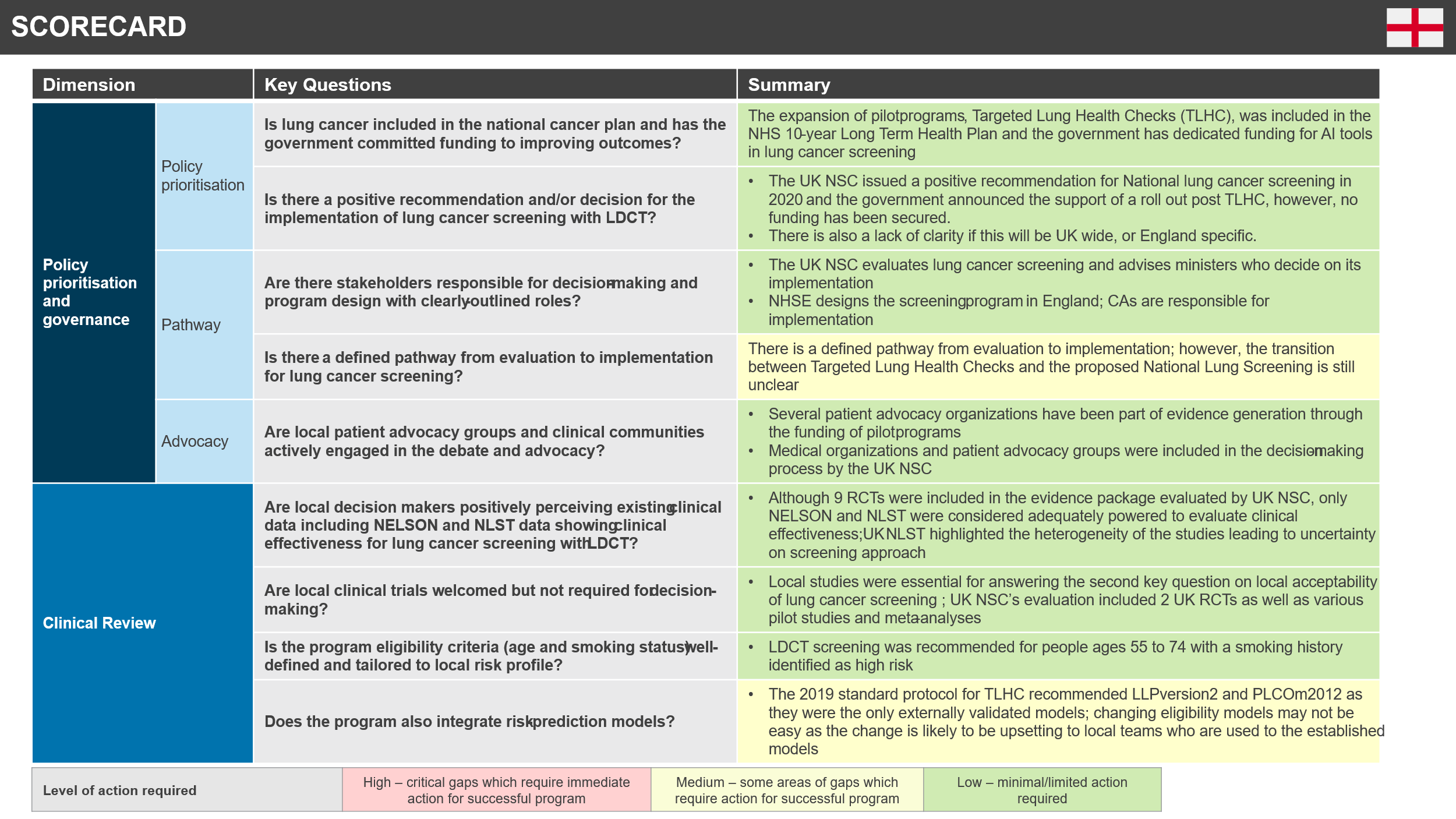


Figure 8b. Assessment for England for program design dimension


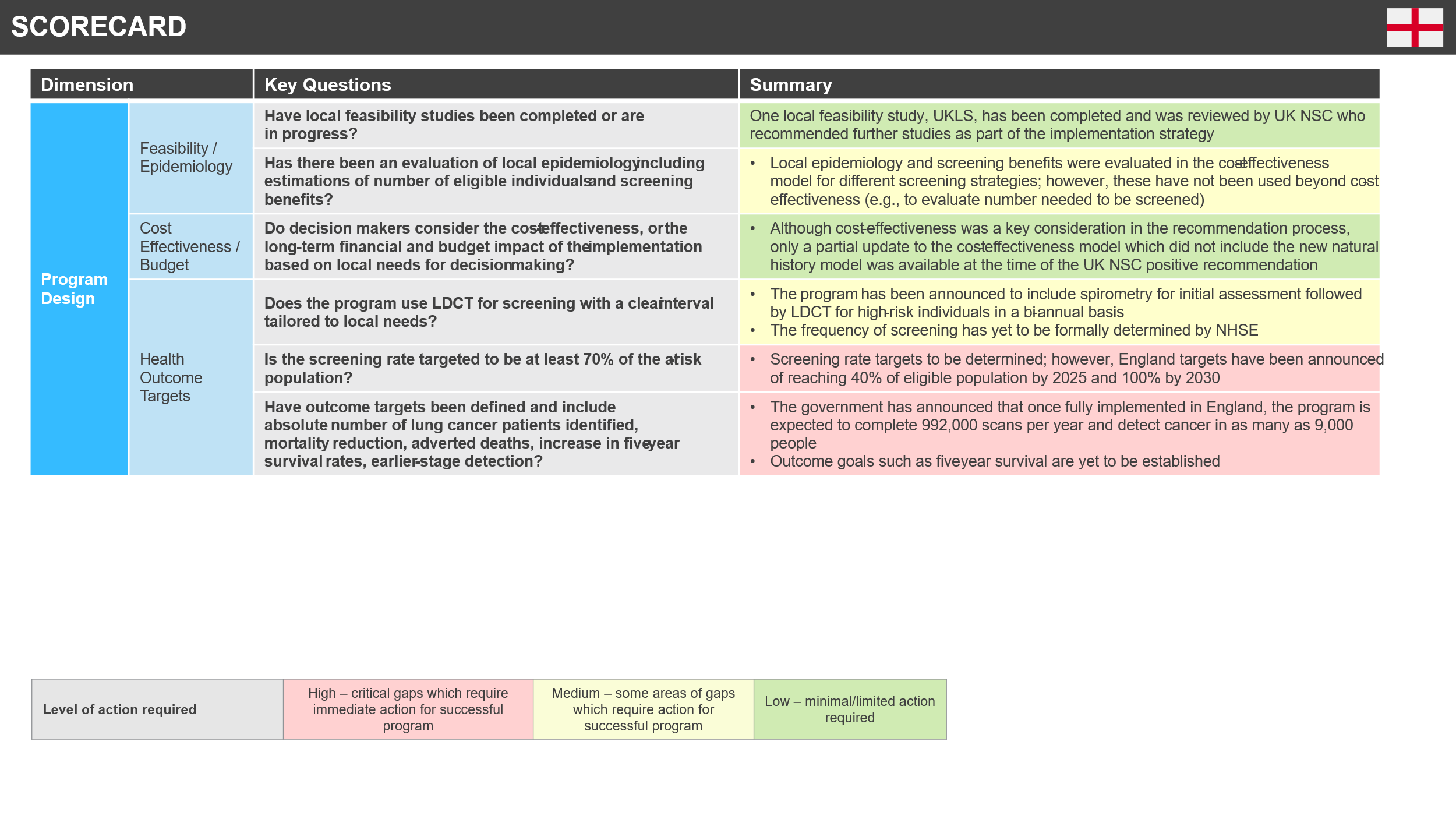


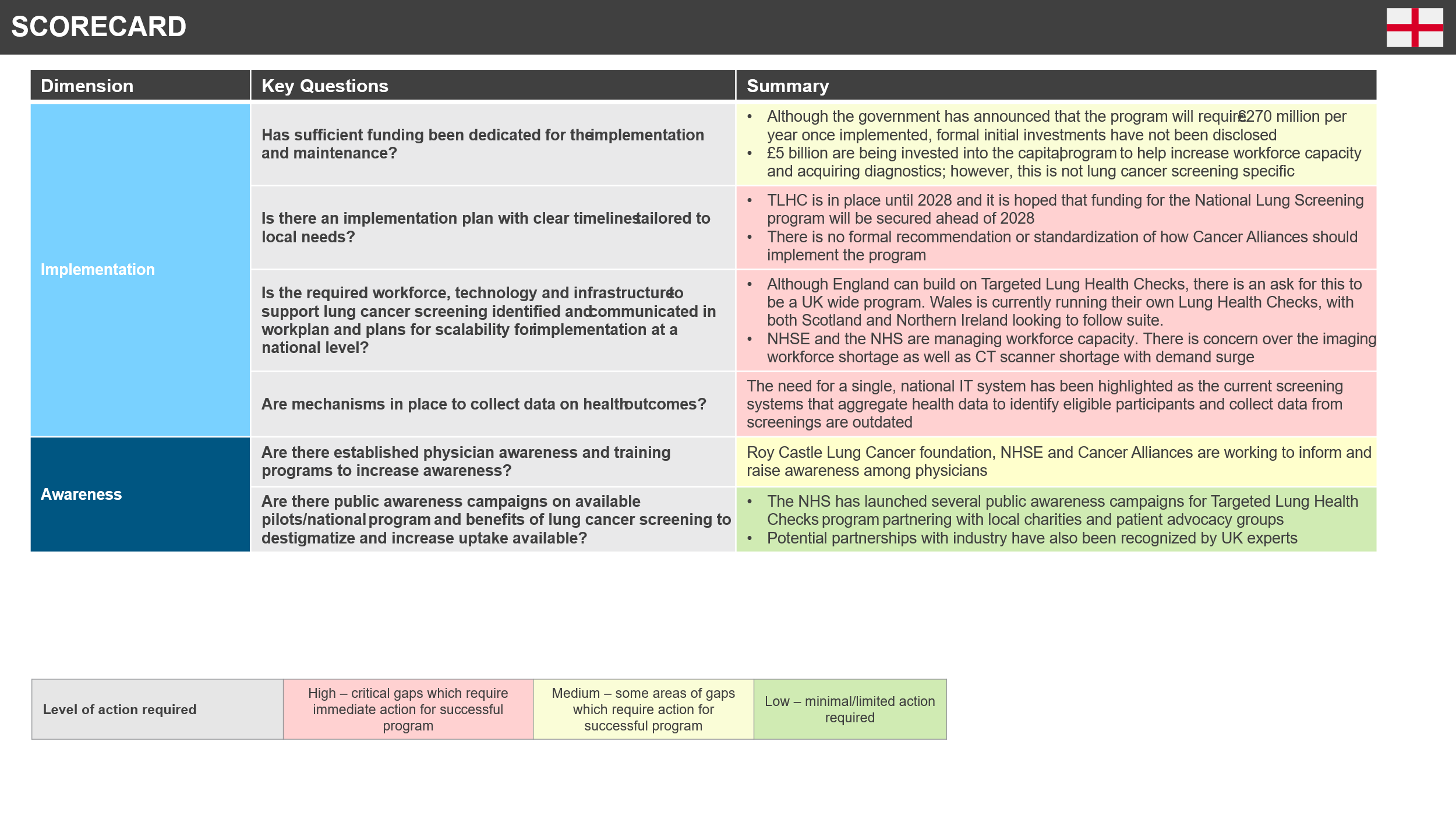
Figure 8c. Assessment for England for implementation & awareness dimensions

## Poland

Figure 9. Timeline of key events in the implementation of a national LCS program in Poland


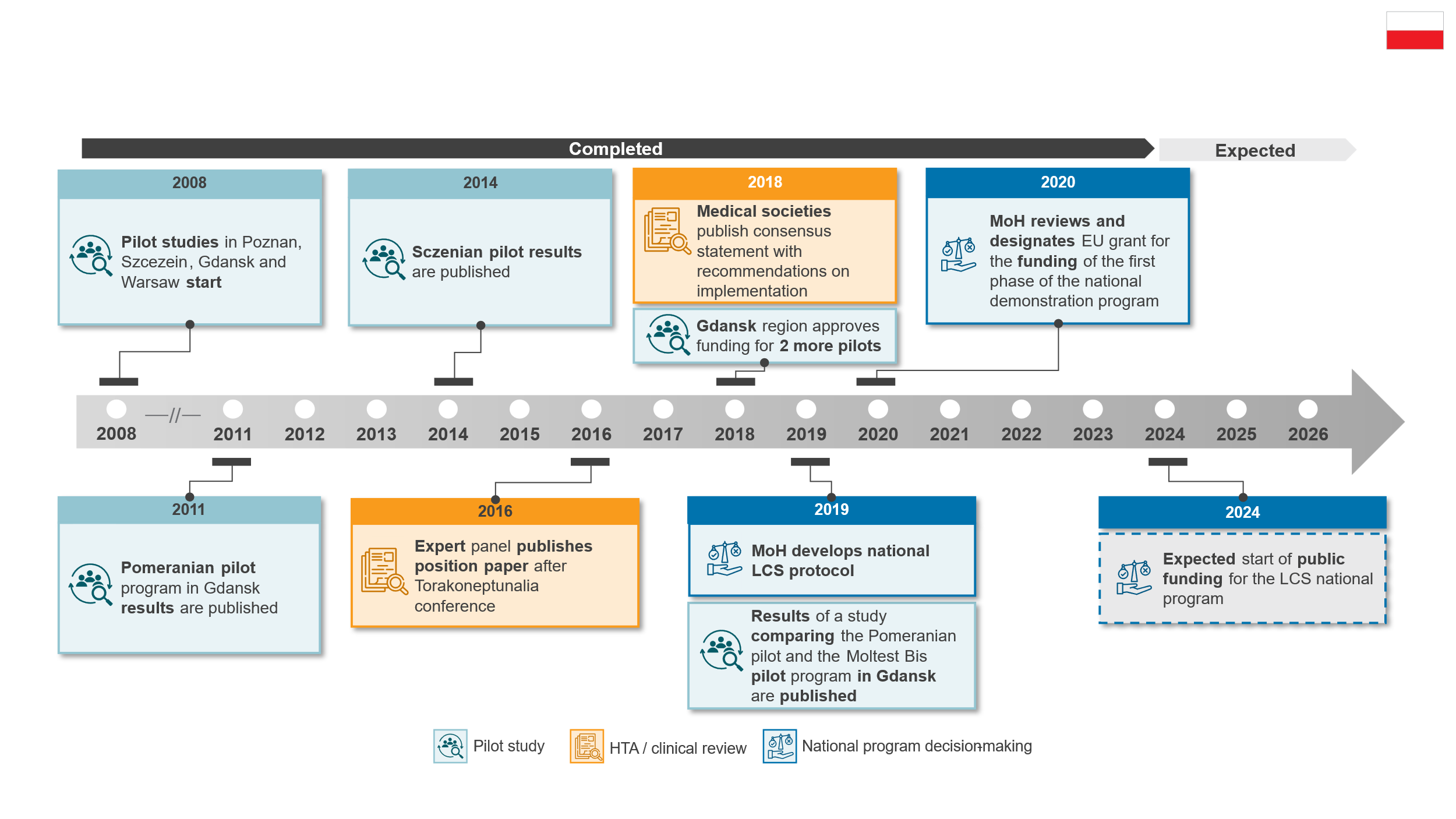


Figure 10a. Assessment for Poland for policy prioritisation & governance and clinical review dimensions


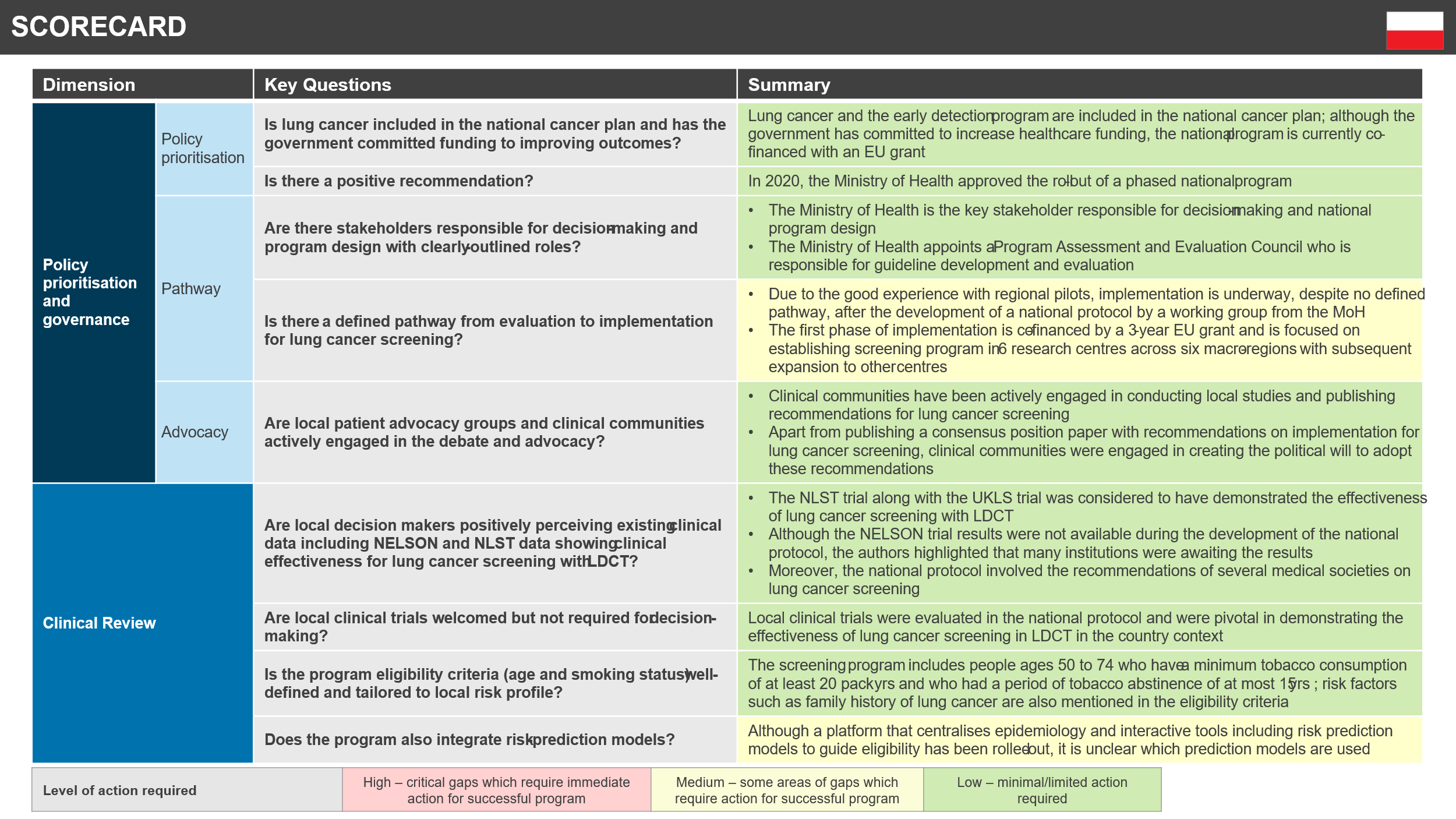


Figure 10b. Assessment for Poland for program design dimension


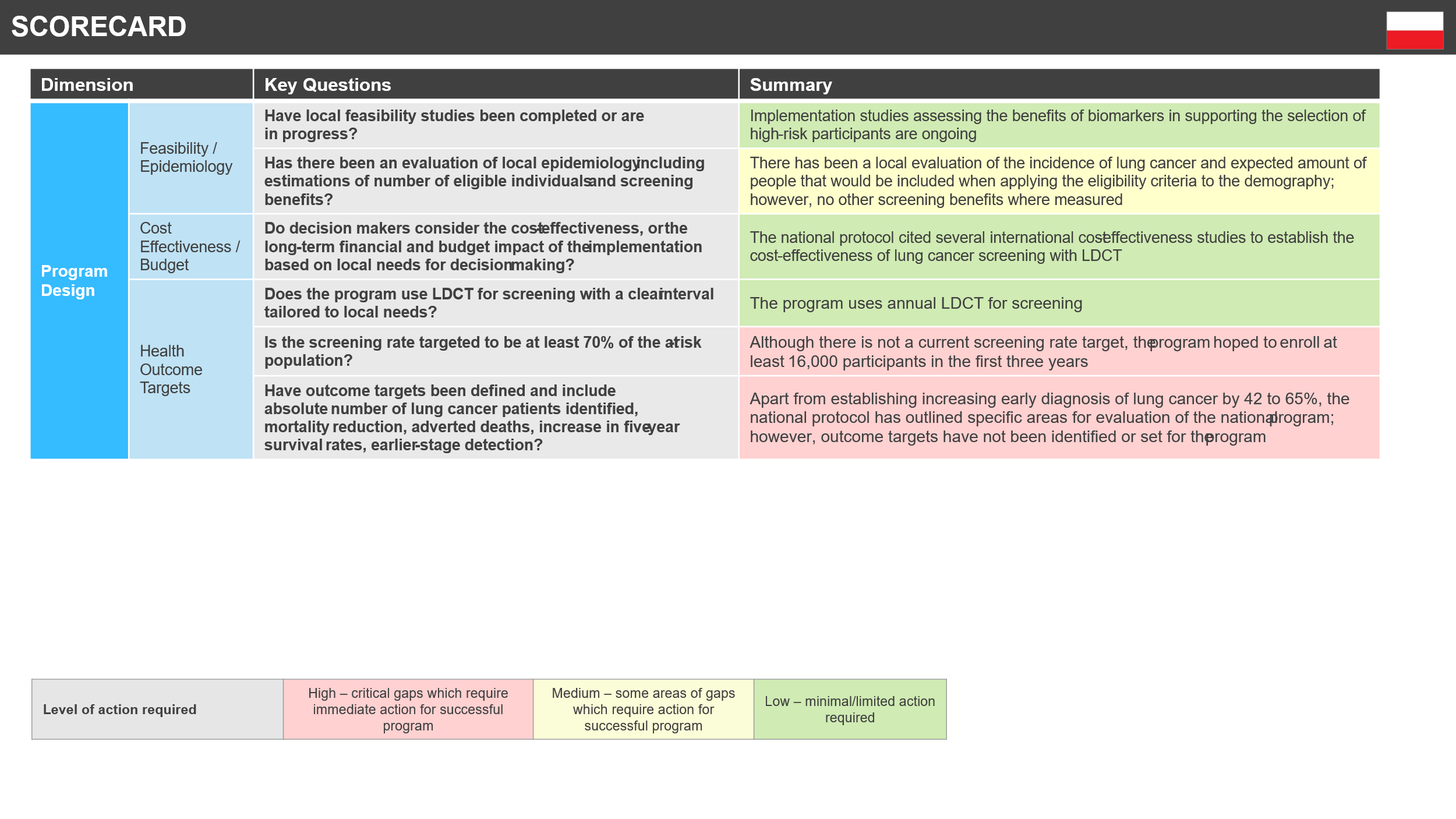


Figure 10c. Assessment for Poland for implementation and awareness dimensions


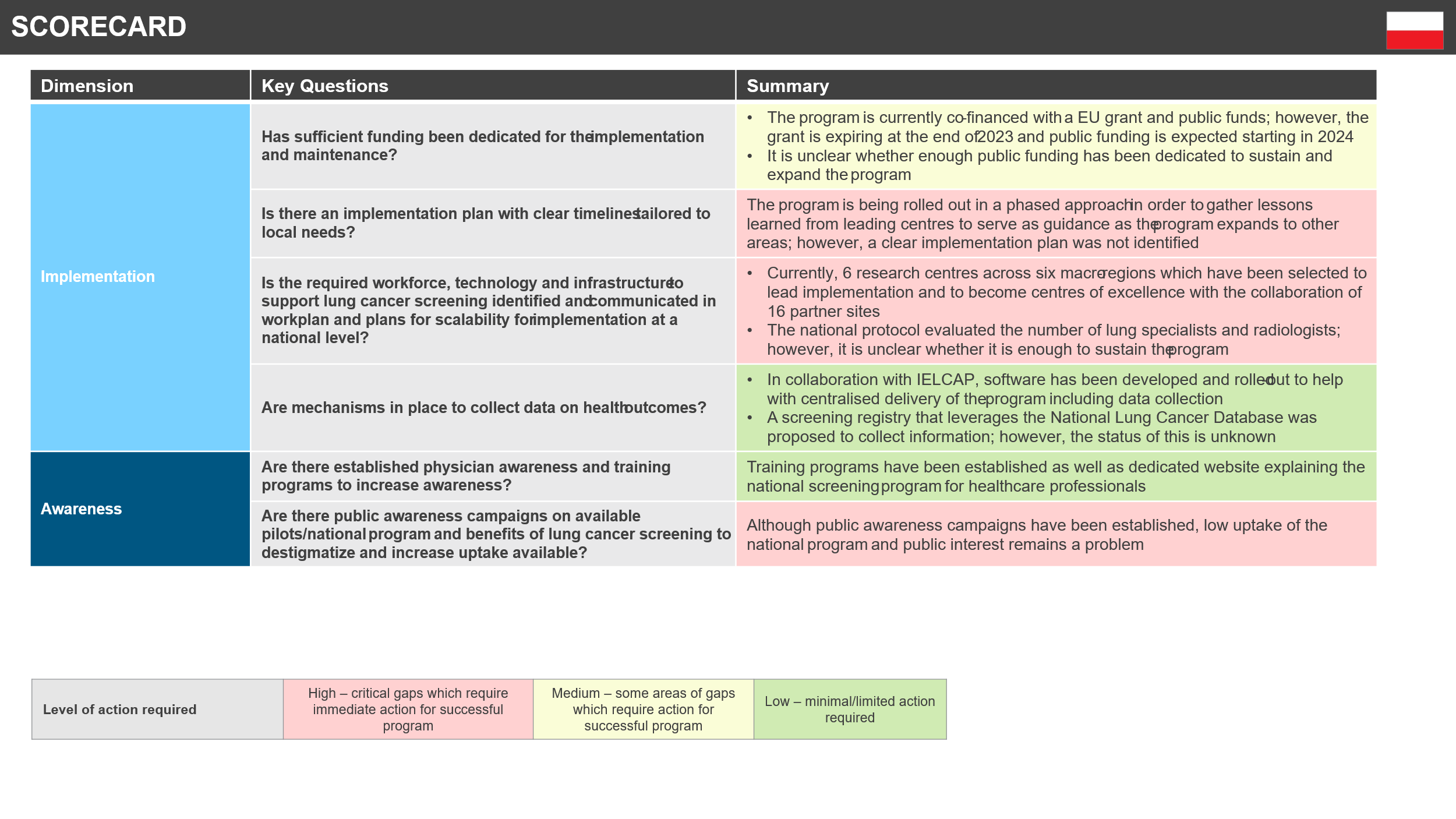


## Belgium

Figure 11. Timeline of key events in the implementation of a national LCS program in Belgium


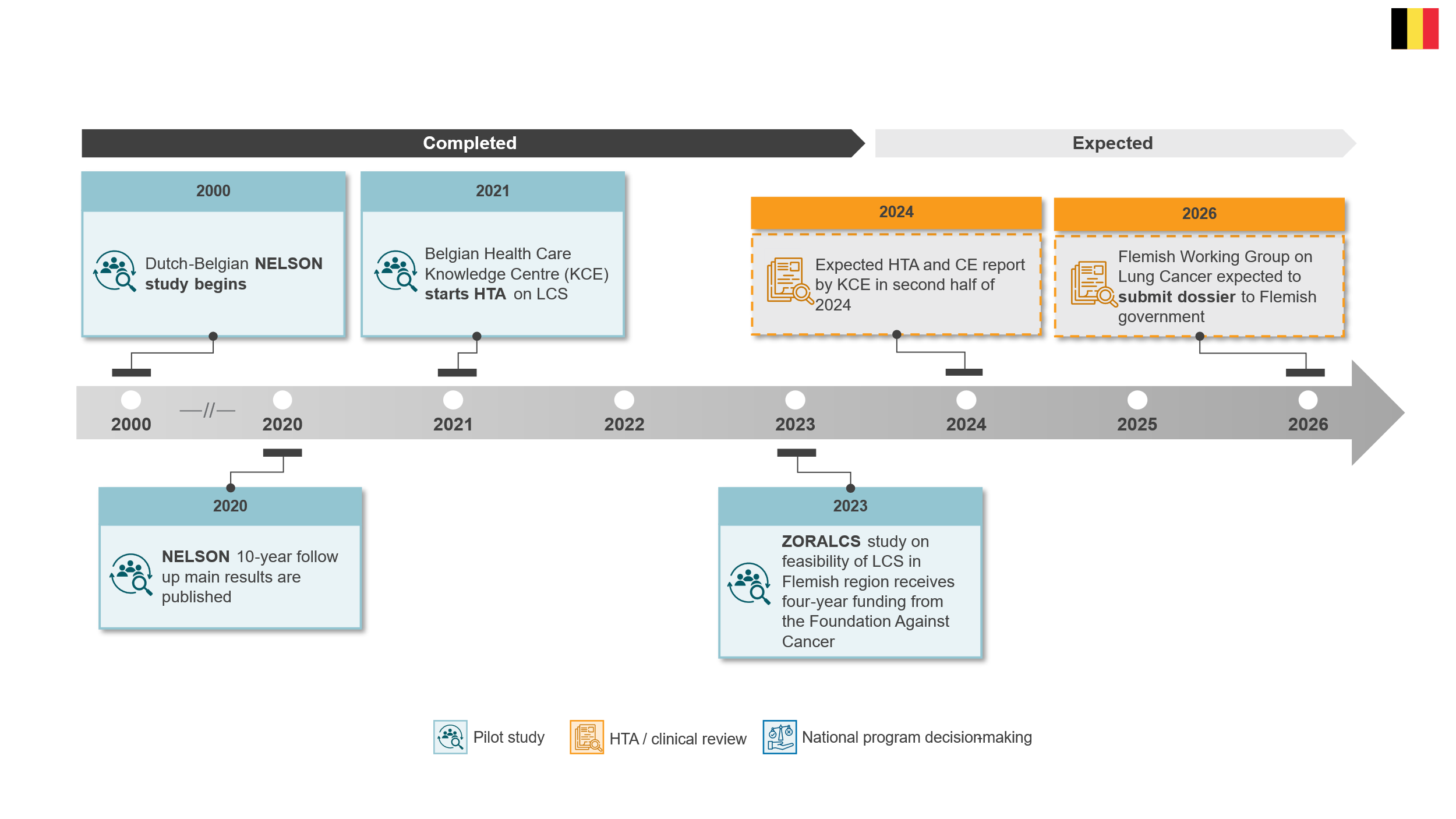


Figure 12a. Assessment for Belgium for policy prioritisation & governance and clinical review dimensions


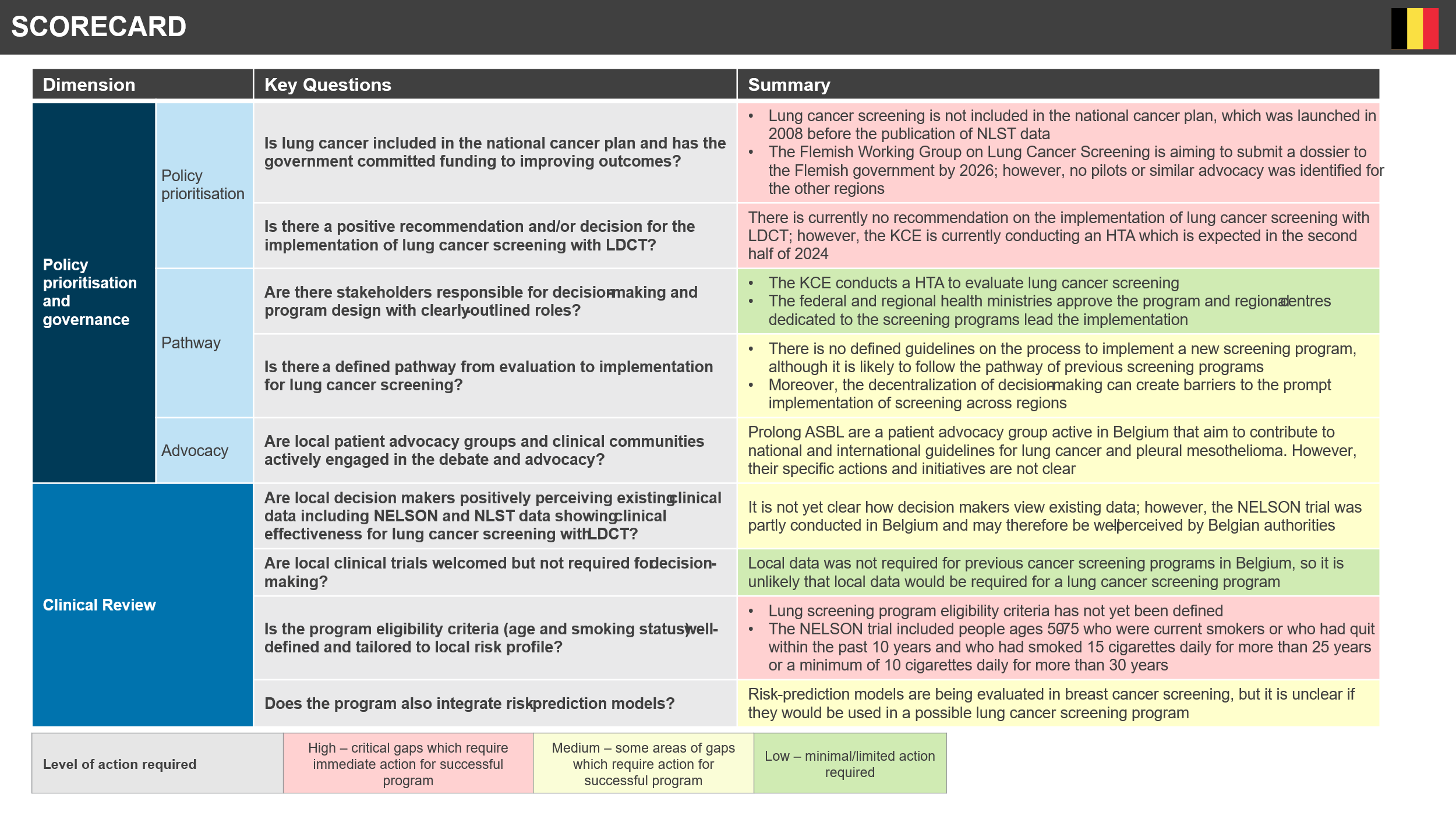


Figure 12b. Assessment for Belgium for program design dimension


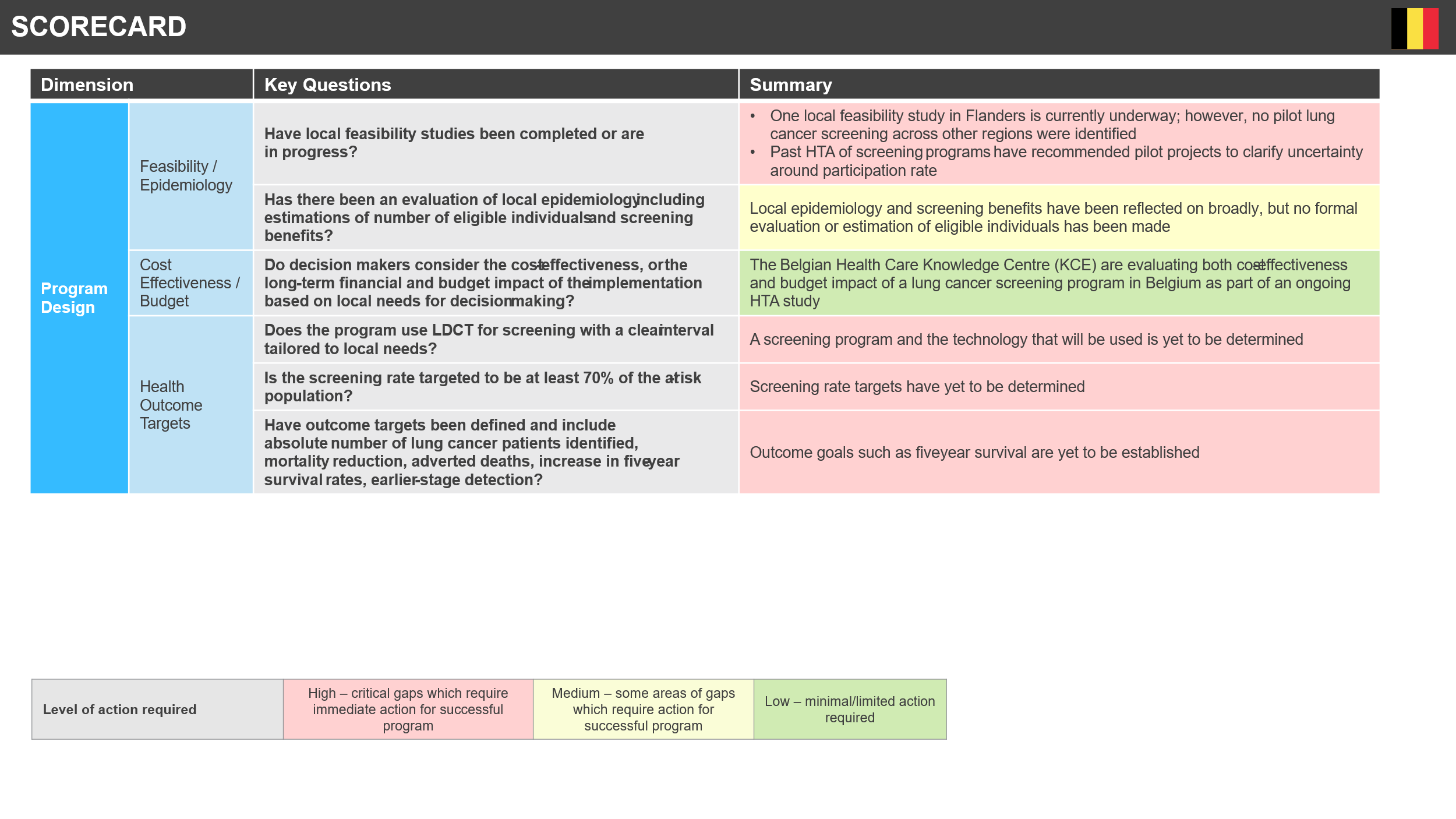


Figure 12c. Assessment for Belgium for implementation and awareness dimensions


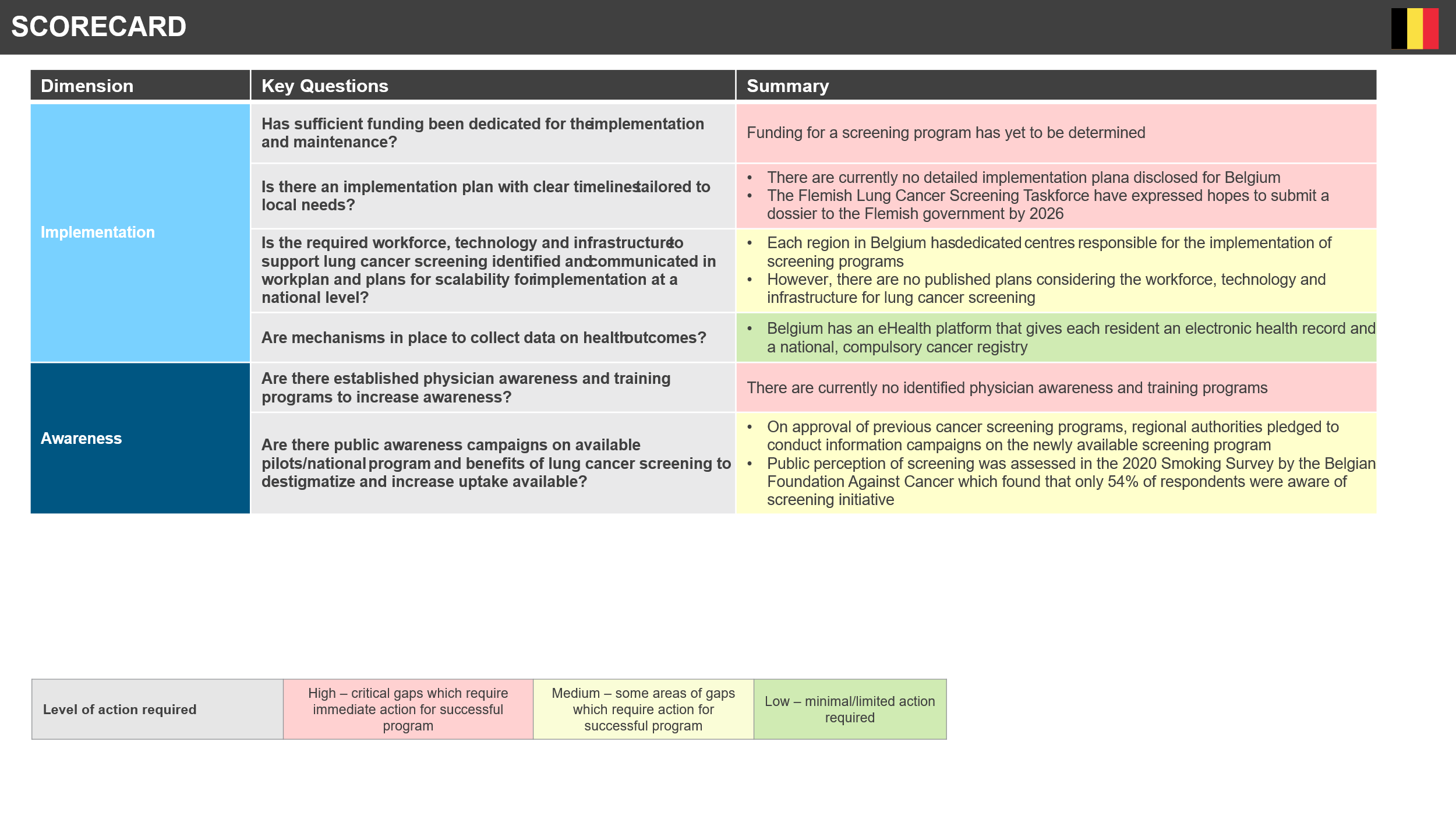


## Germany


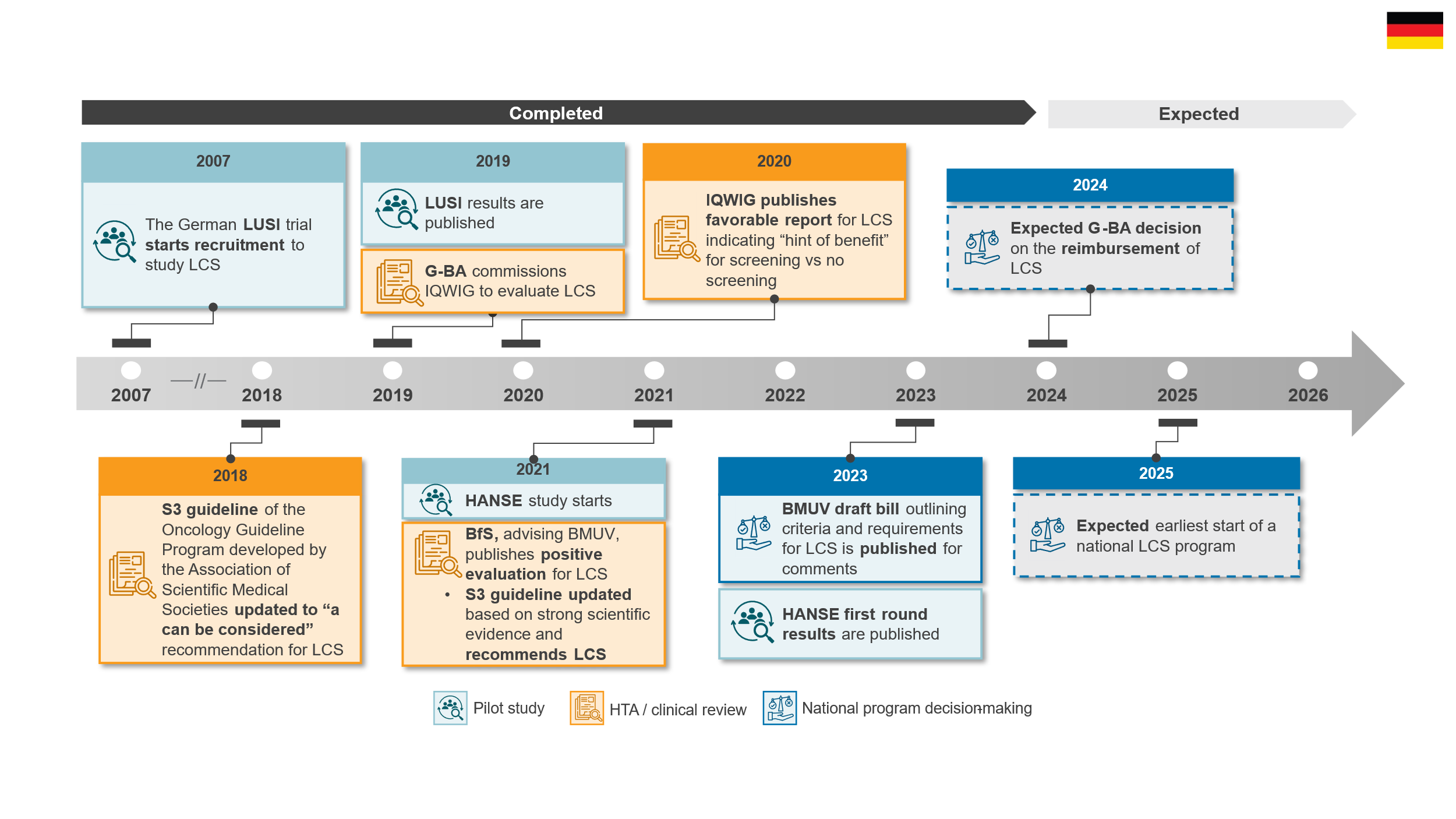
Figure 13. Timeline of key events in the implementation of a national LCS program in Germany


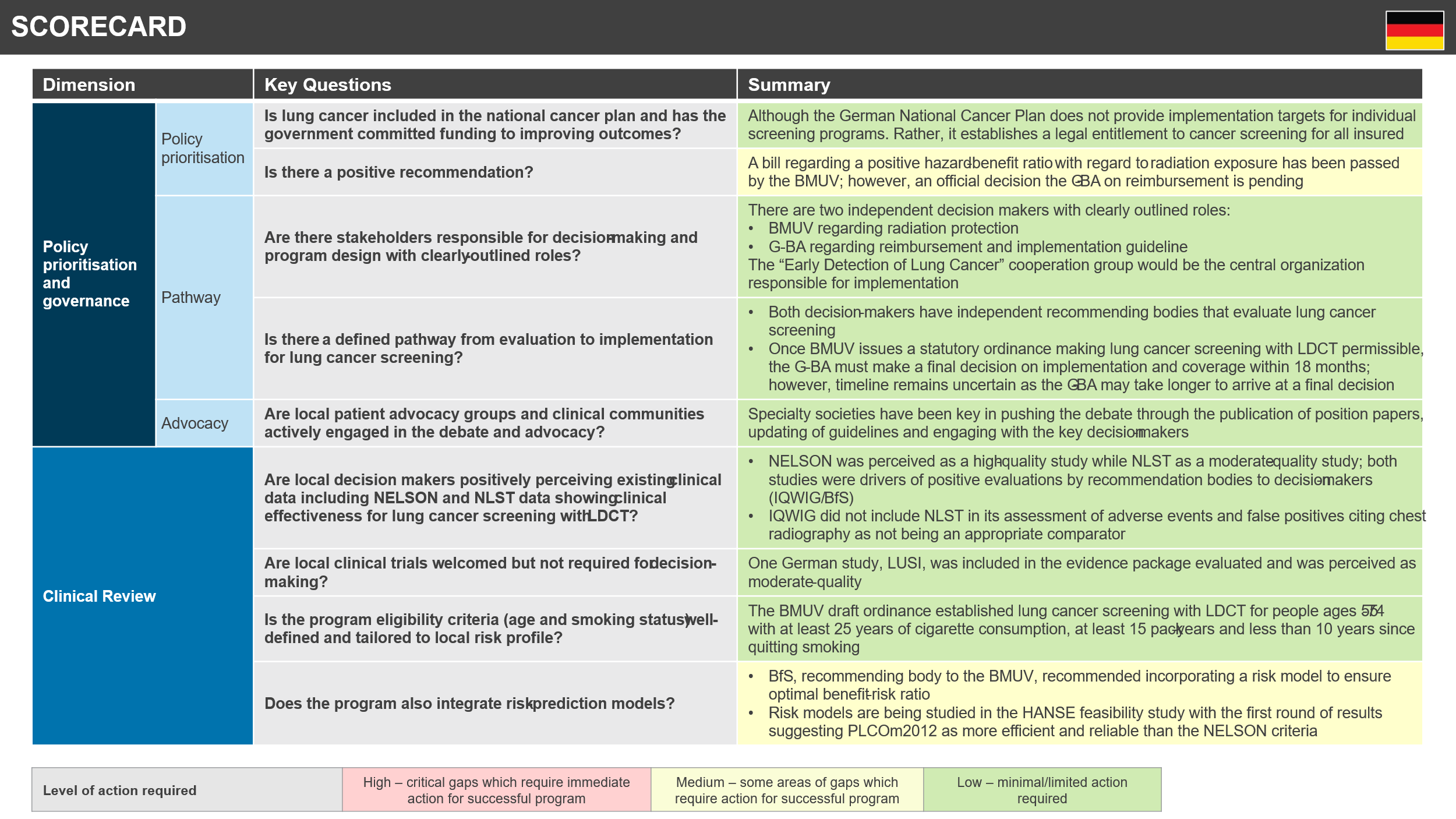
Figure 14a. Assessment for Germany for policy prioritisation & governance and clinical review dimensions

Figure 14b. Assessment for Germany for program design dimension


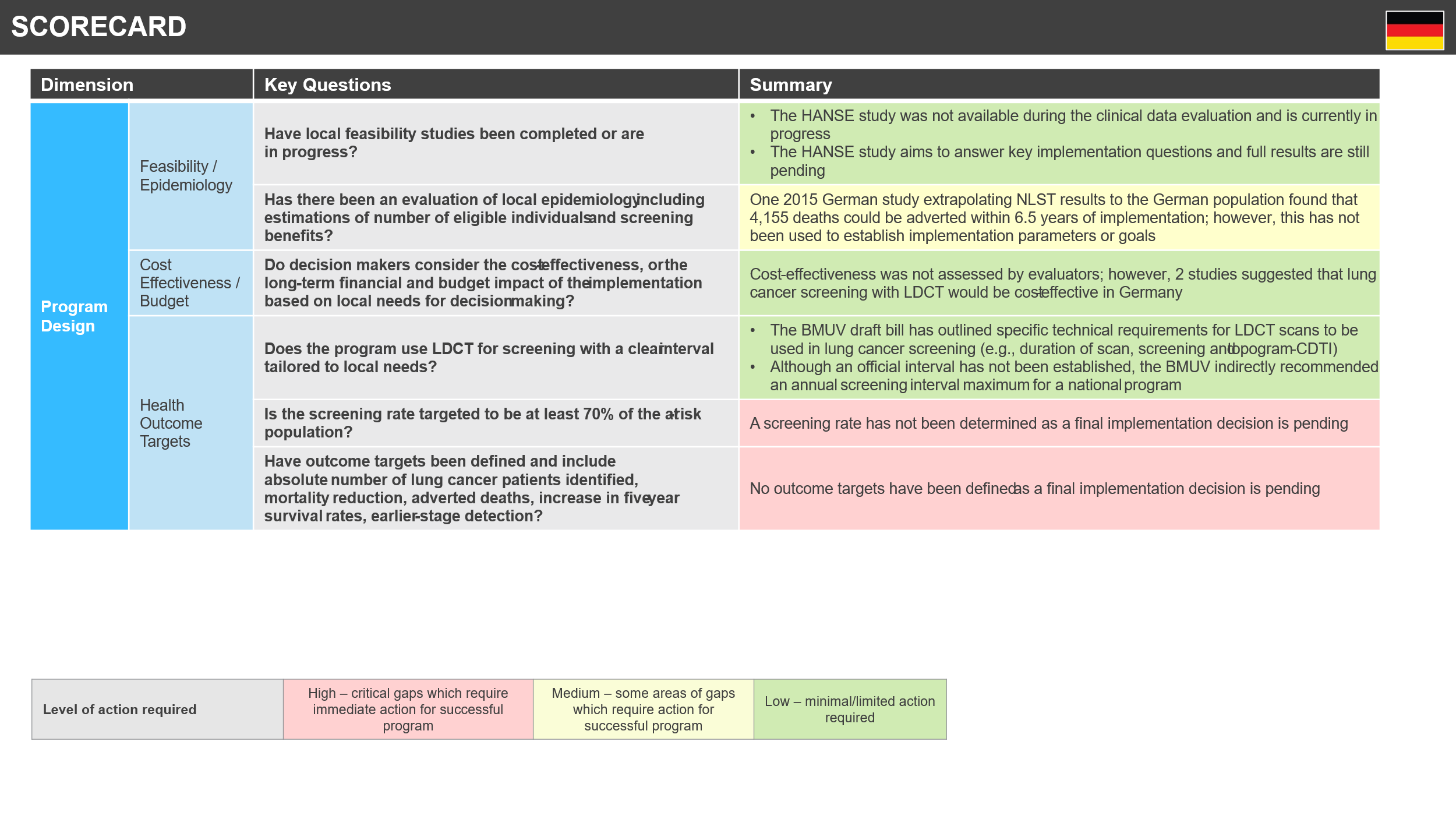


Figure 14c. Assessment for Poland for implementation and awareness dimensions


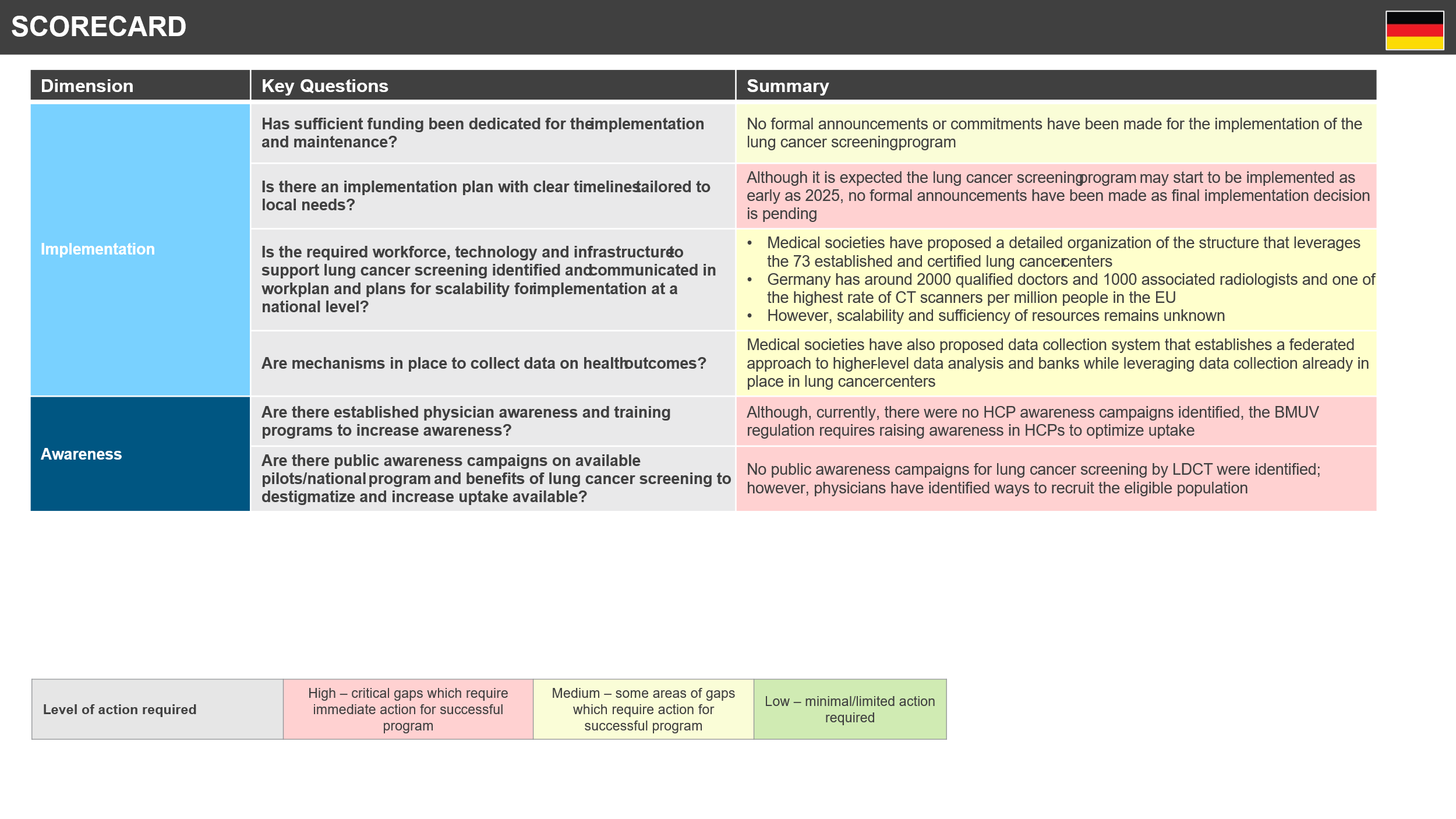


## Italy


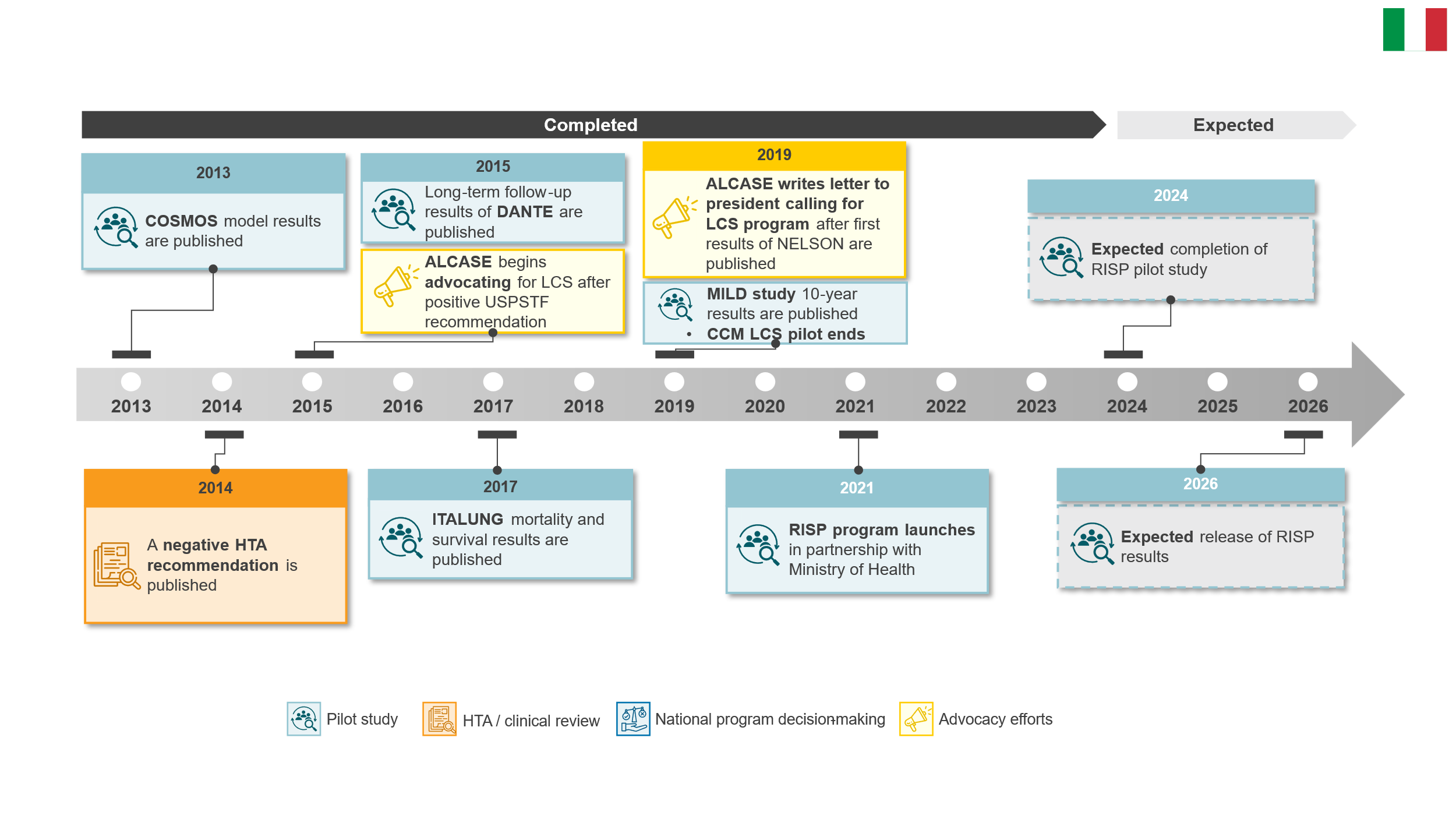
Figure 15. Timeline of key events in the implementation of a national LCS program in Italy


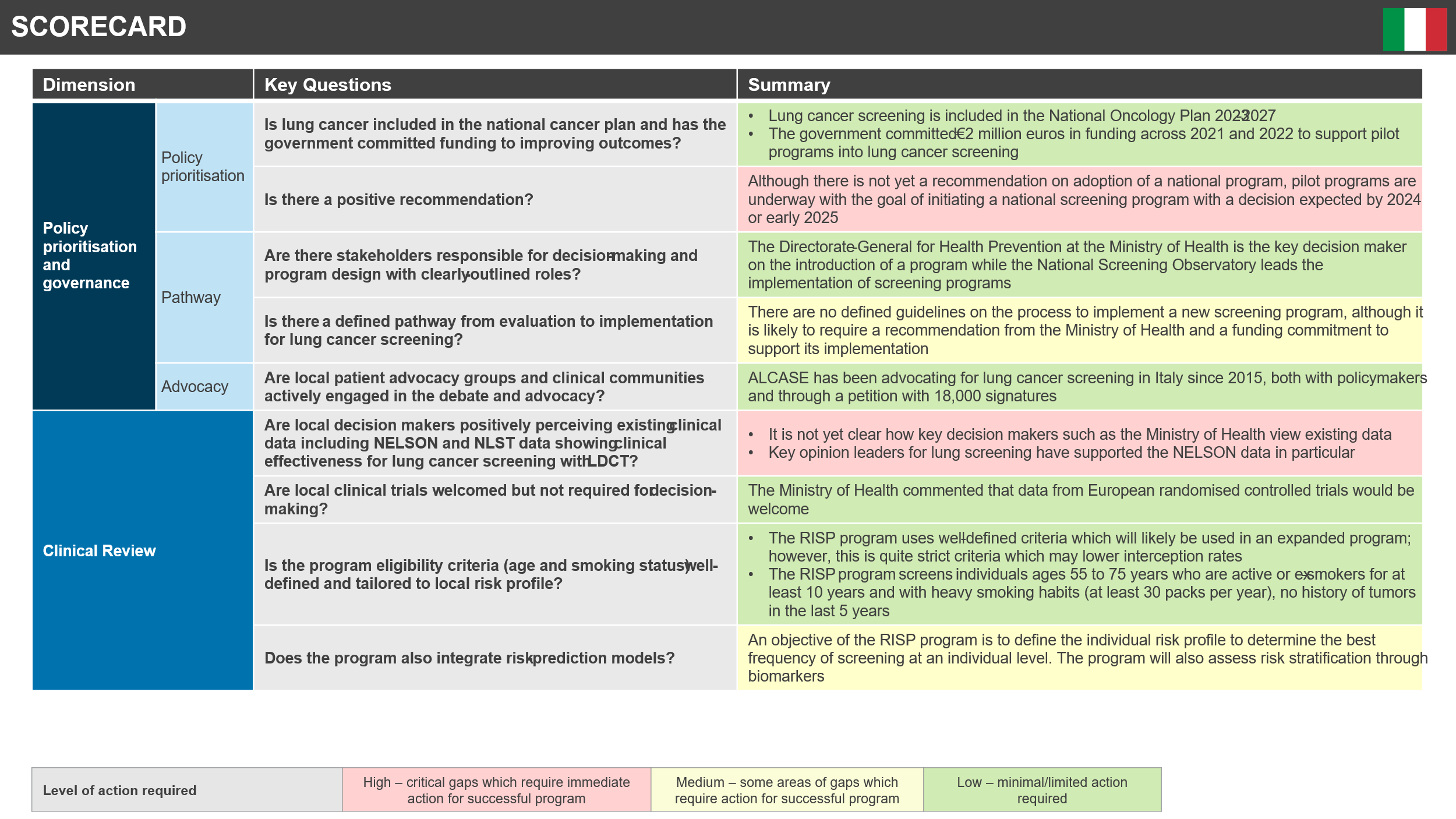
Figure 16a. Assessment for Italy for policy prioritisation & governance and clinical review dimensions

Figure 16b. Assessment for Italy for program design dimension


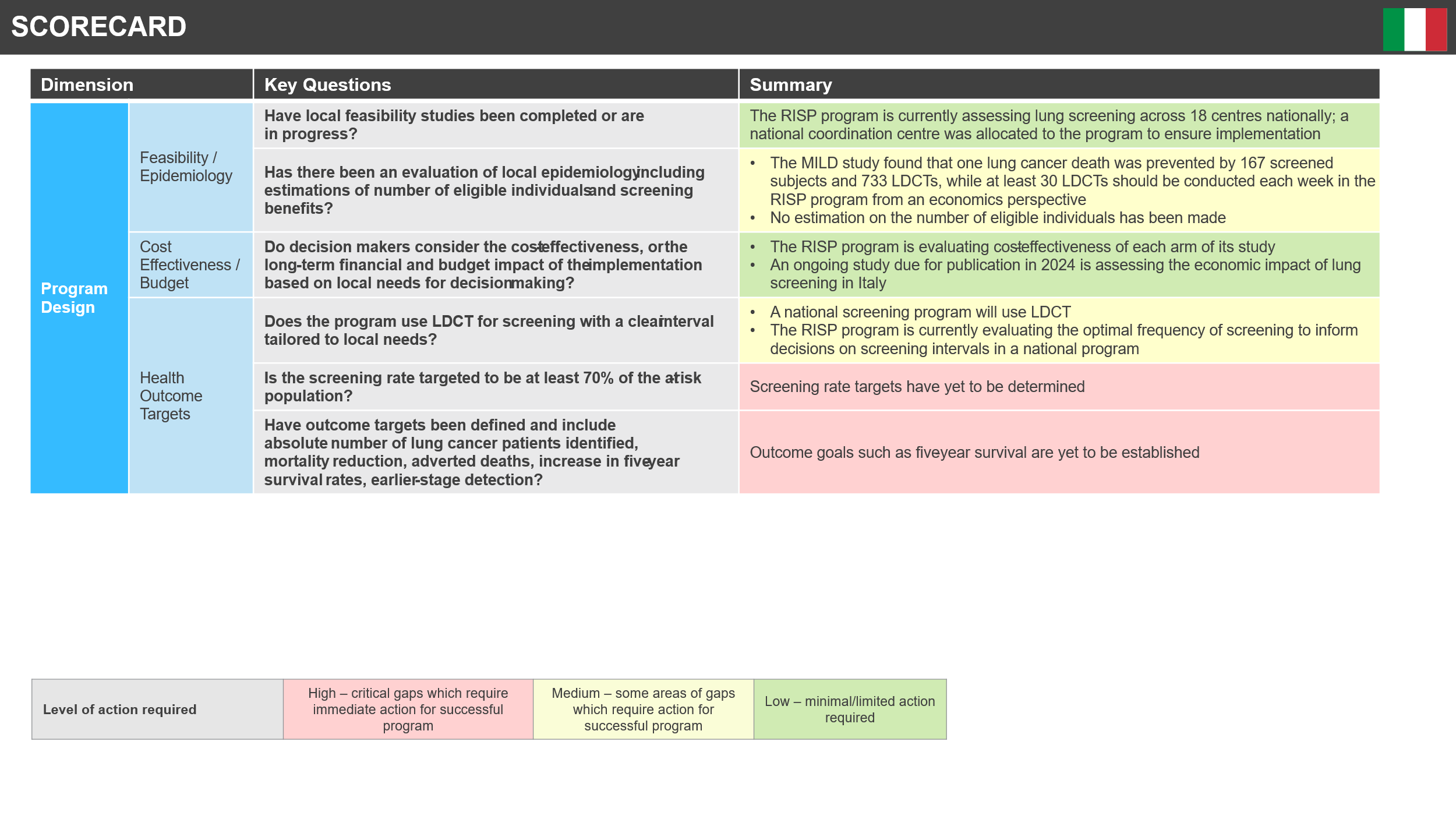


Figure 16c. Assessment for Italy for implementation and awareness dimensions


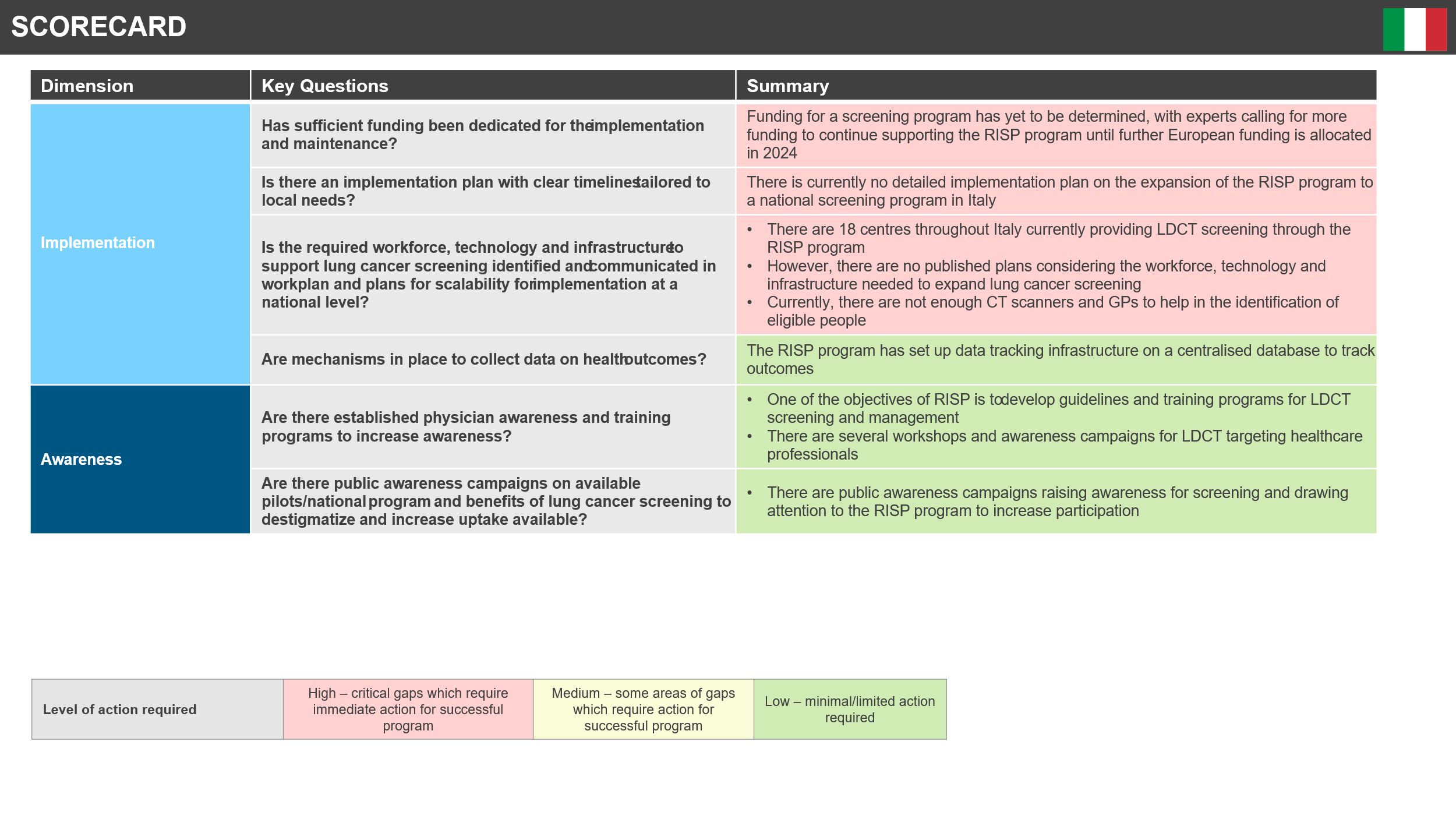


## Spain

Figure 17. Timeline of key events in the implementation of a national LCS program in Spain


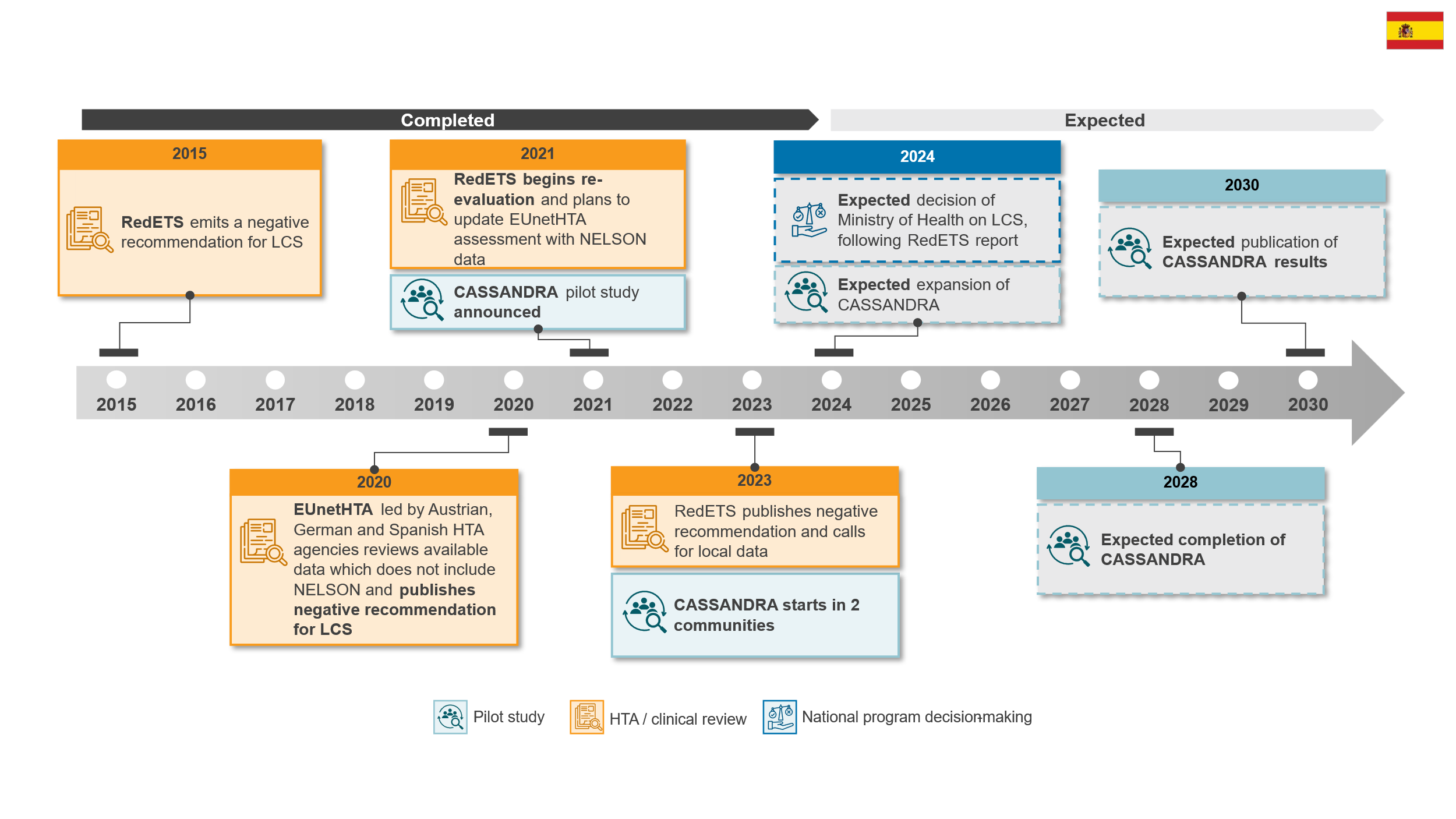


Figure 18a. Assessment for Spain for policy prioritisation & governance and clinical review dimensions


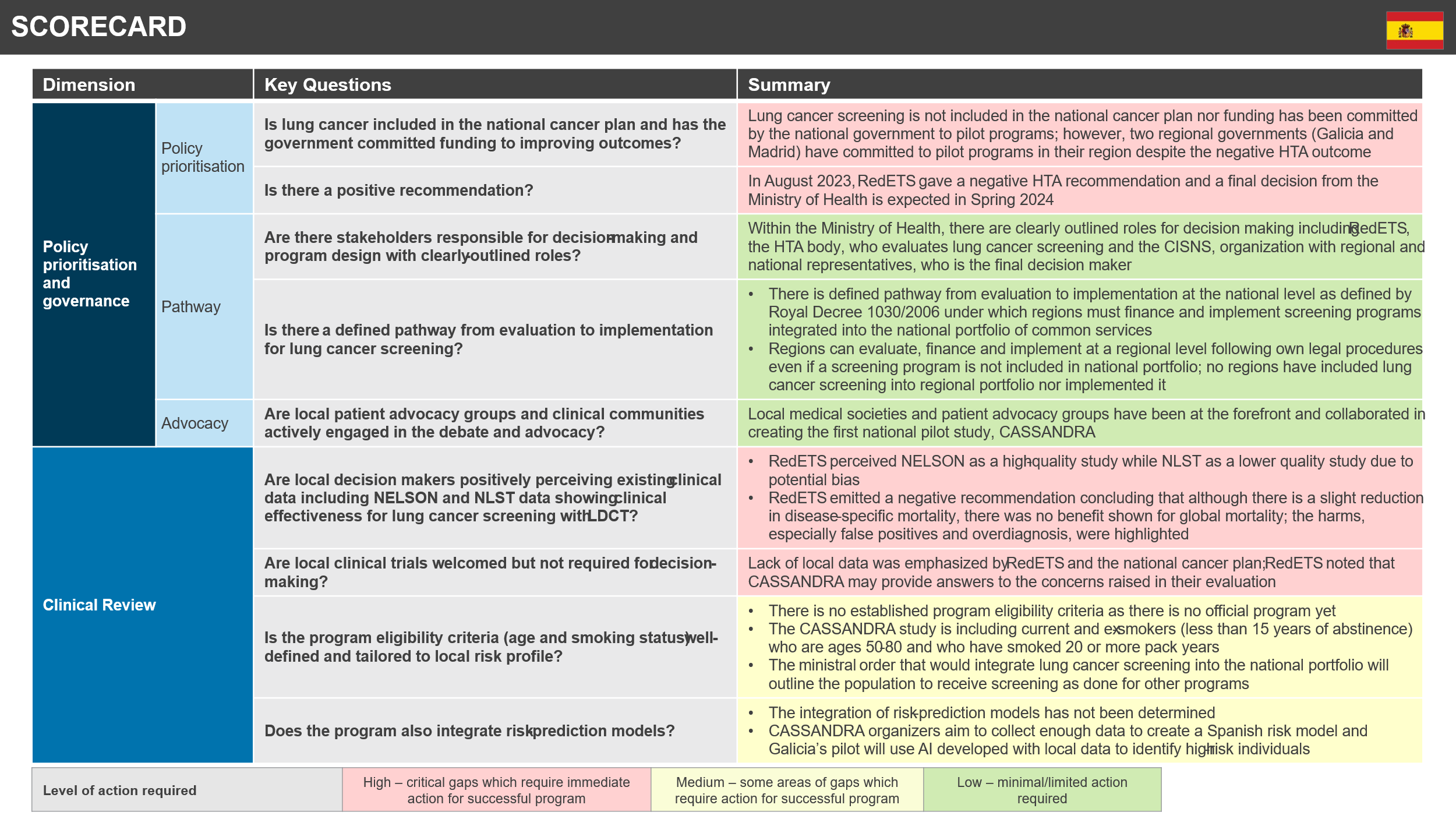


Figure 18b. Framework assessment for Spain for program design dimension


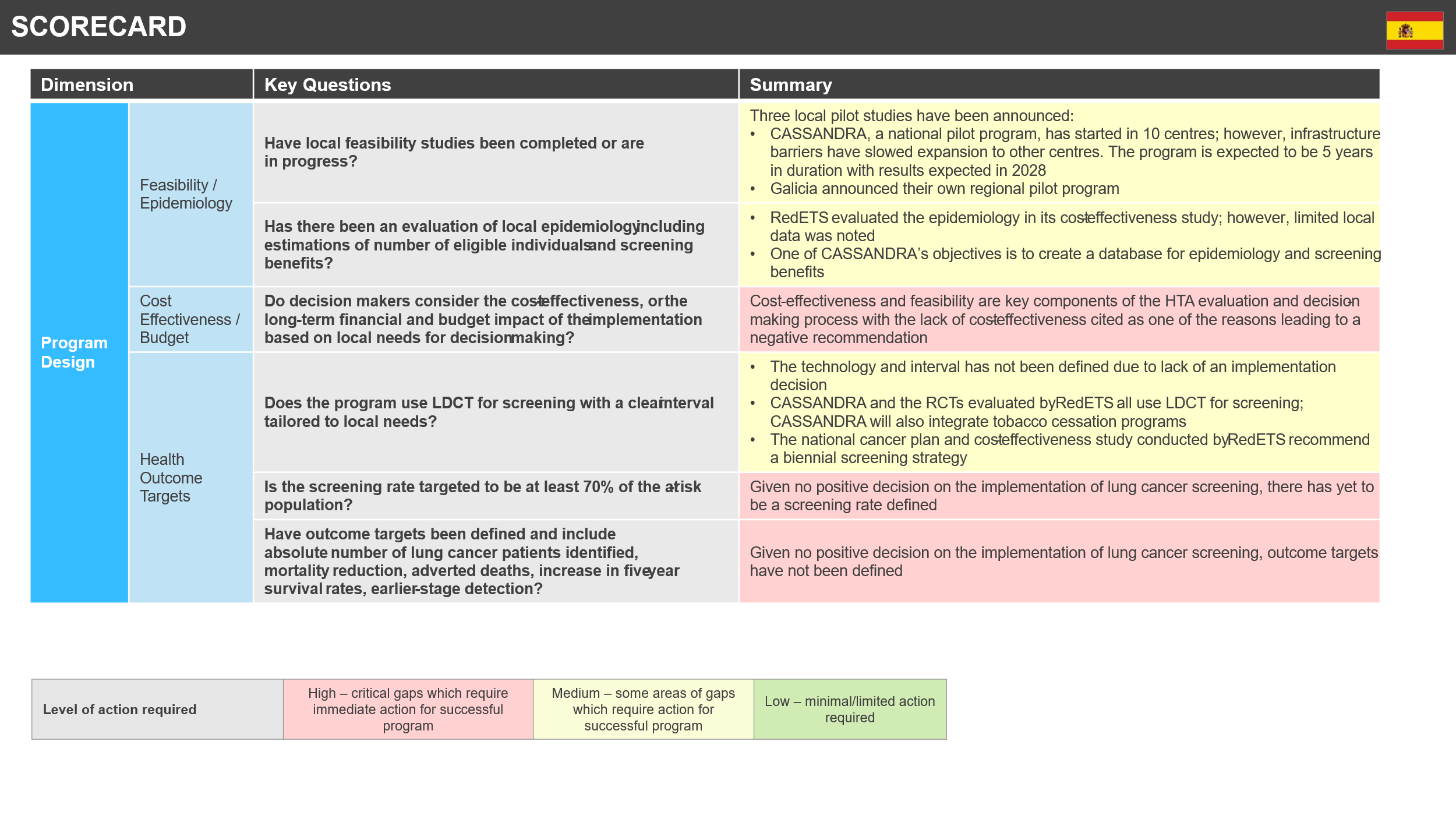


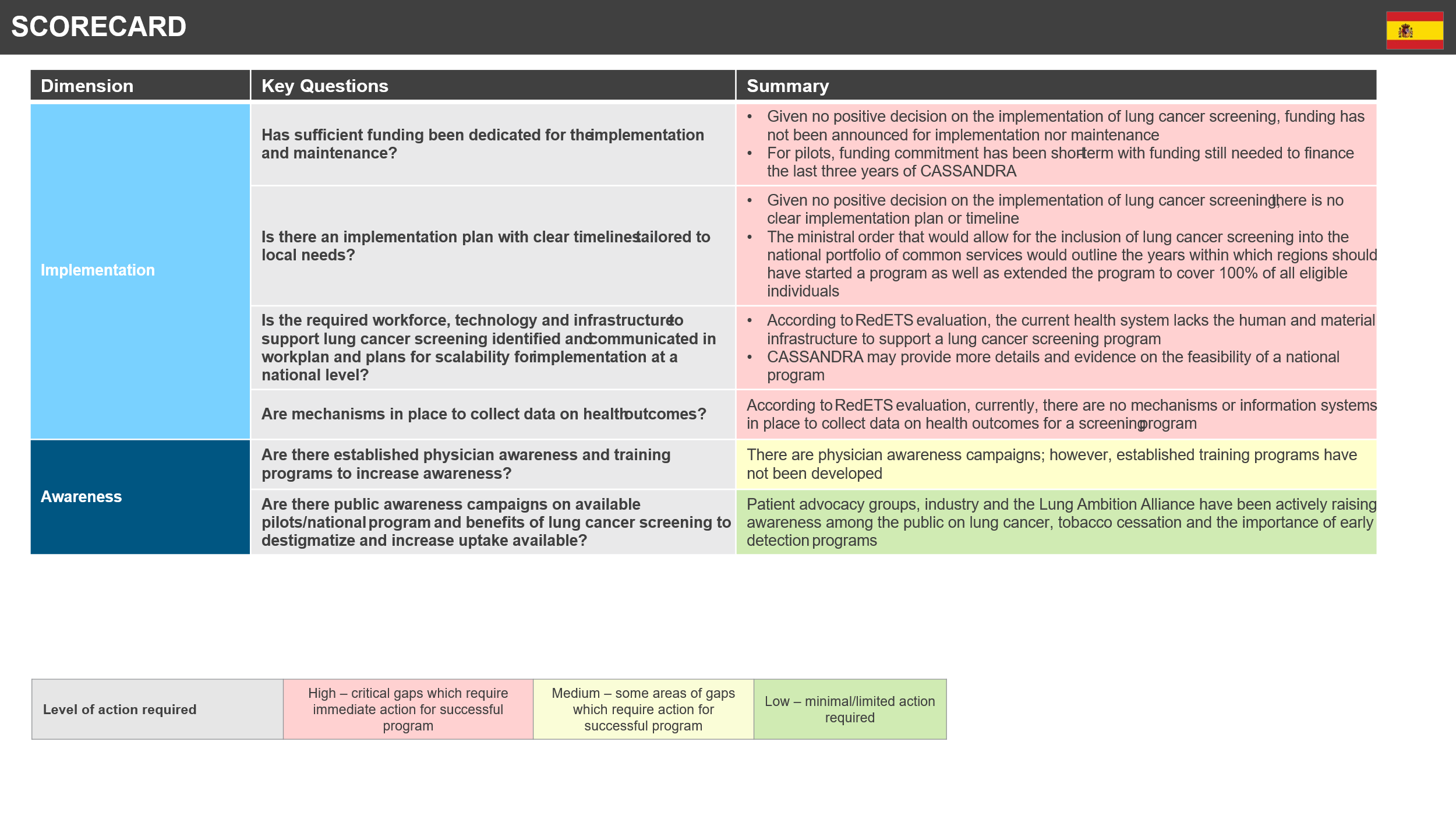
Figure 18c. Assessment for Spain for implementation and awareness dimensions

## Sweden


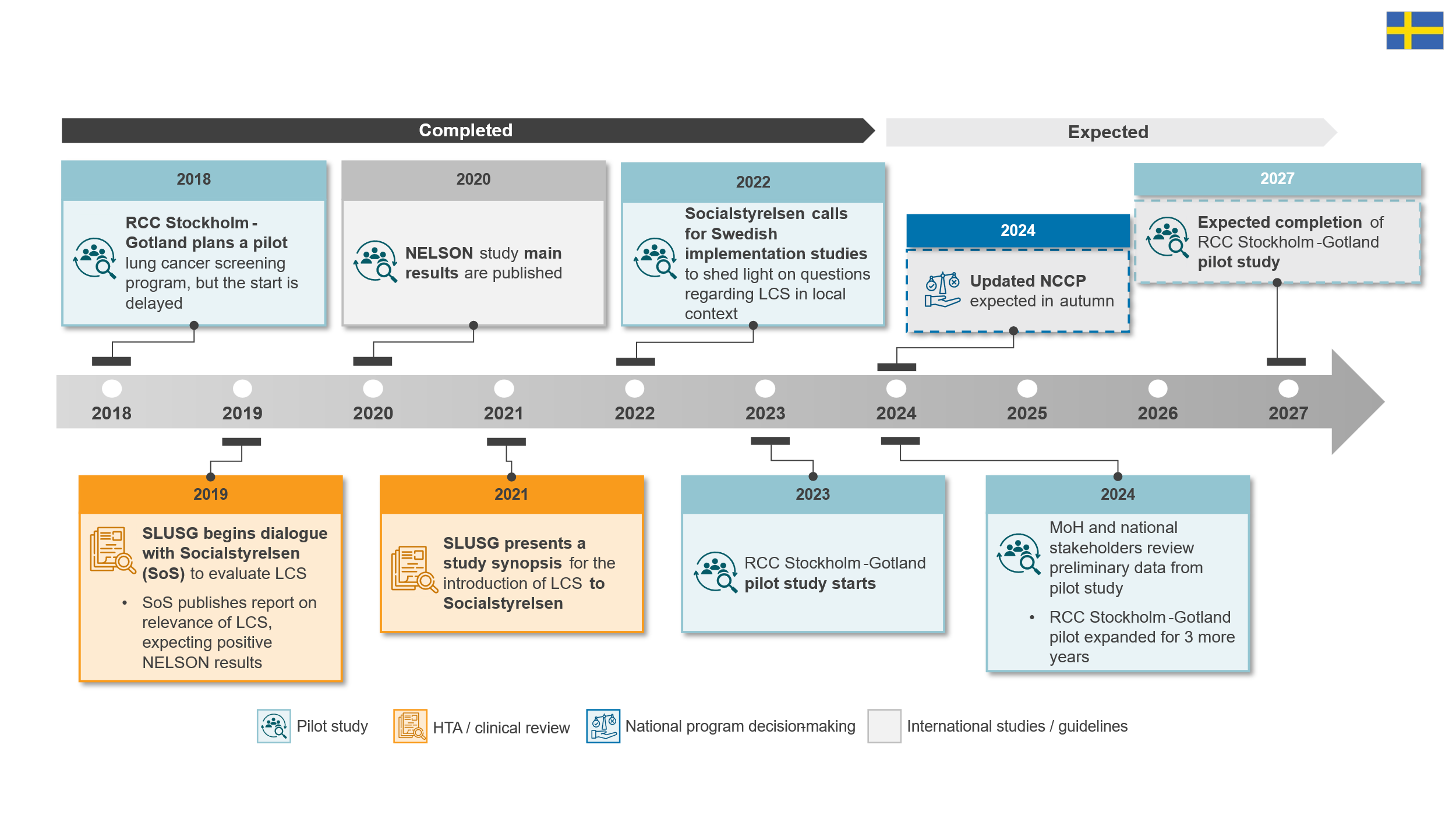
Figure 19. Timeline of key events in the implementation of a national LCS program in Sweden


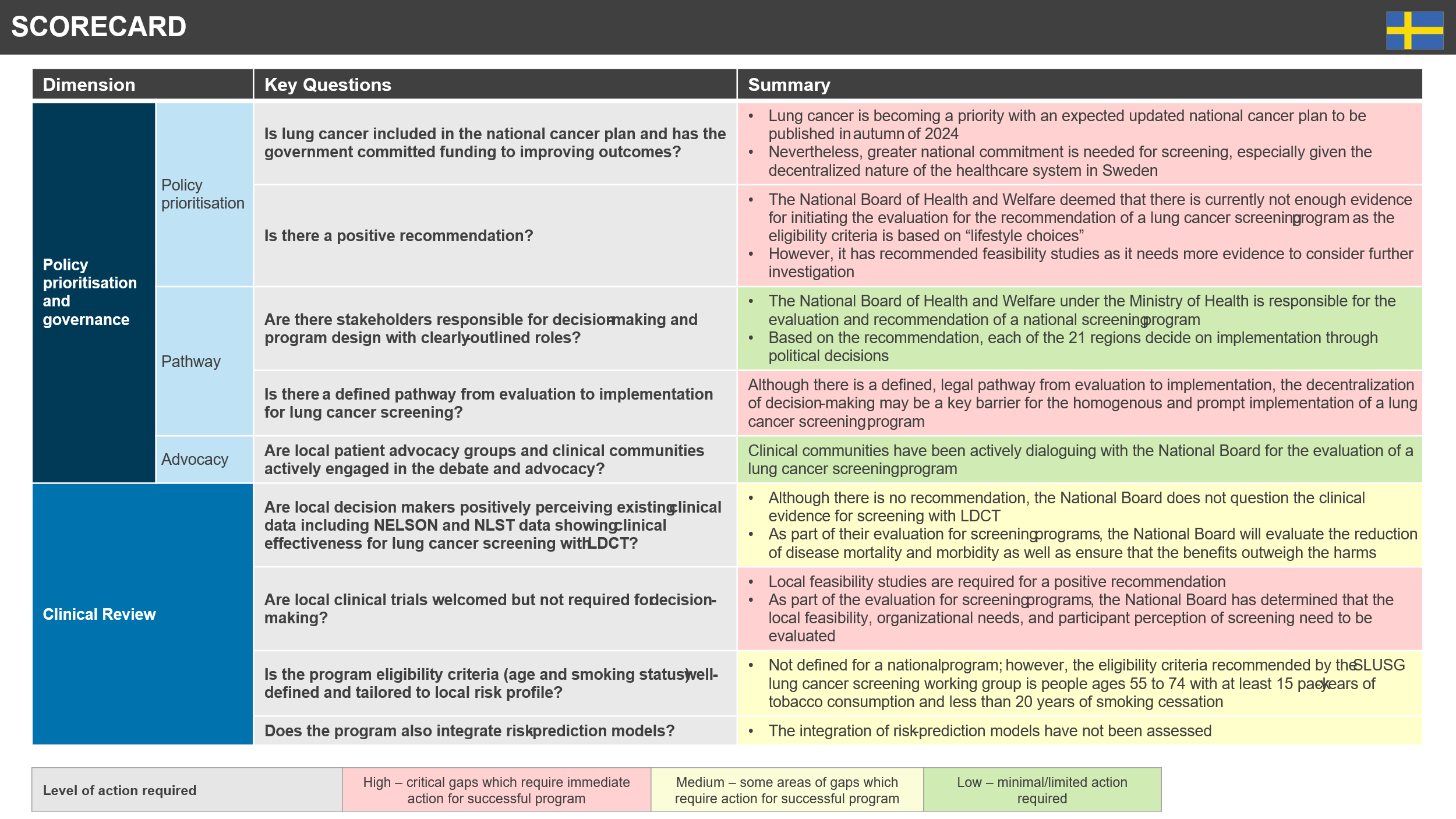
Figure 20a. Assessment for Sweden for policy prioritisation & governance and clinical review dimensions


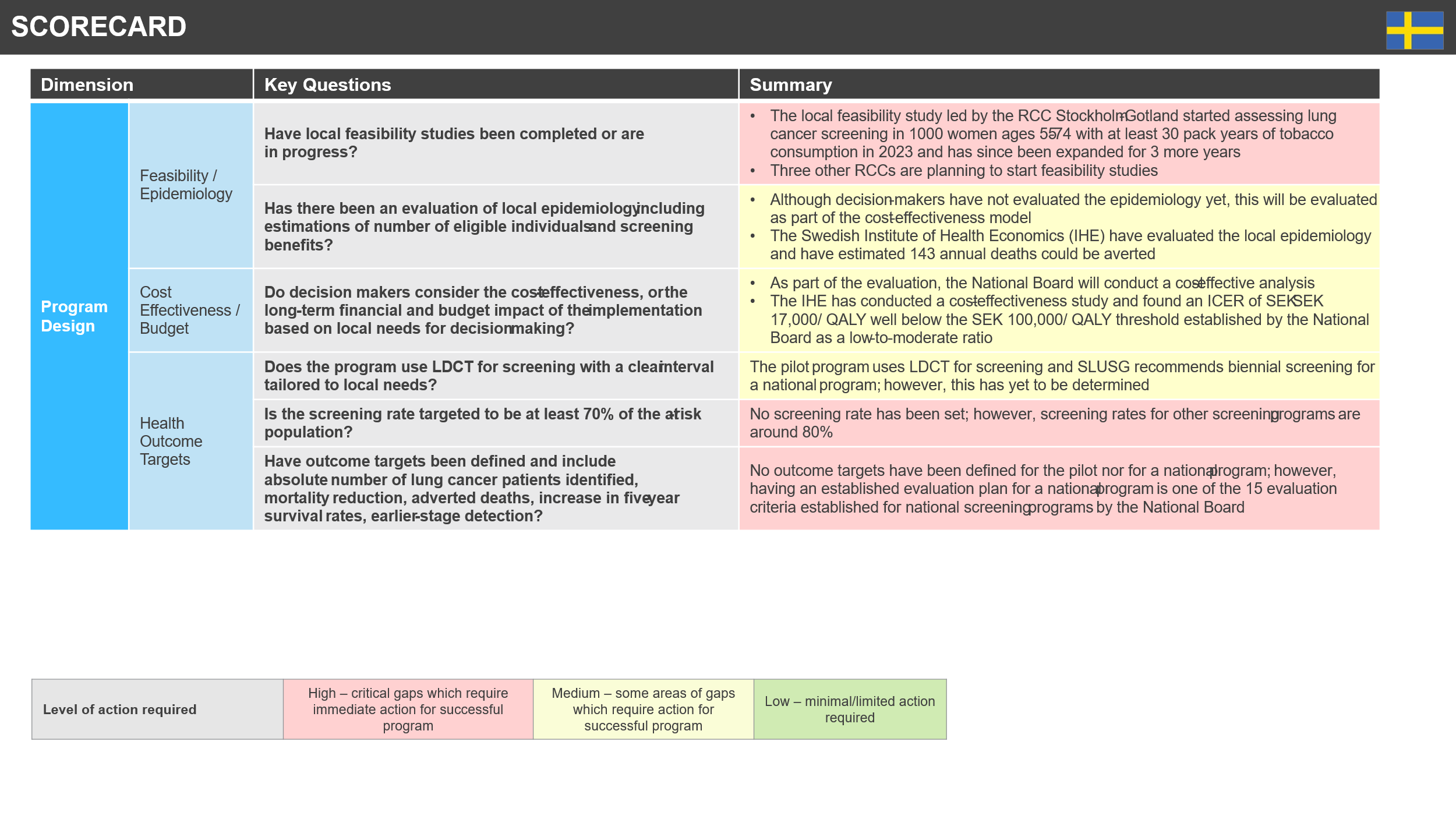
Figure 20b. Assessment for Sweden for program design dimension


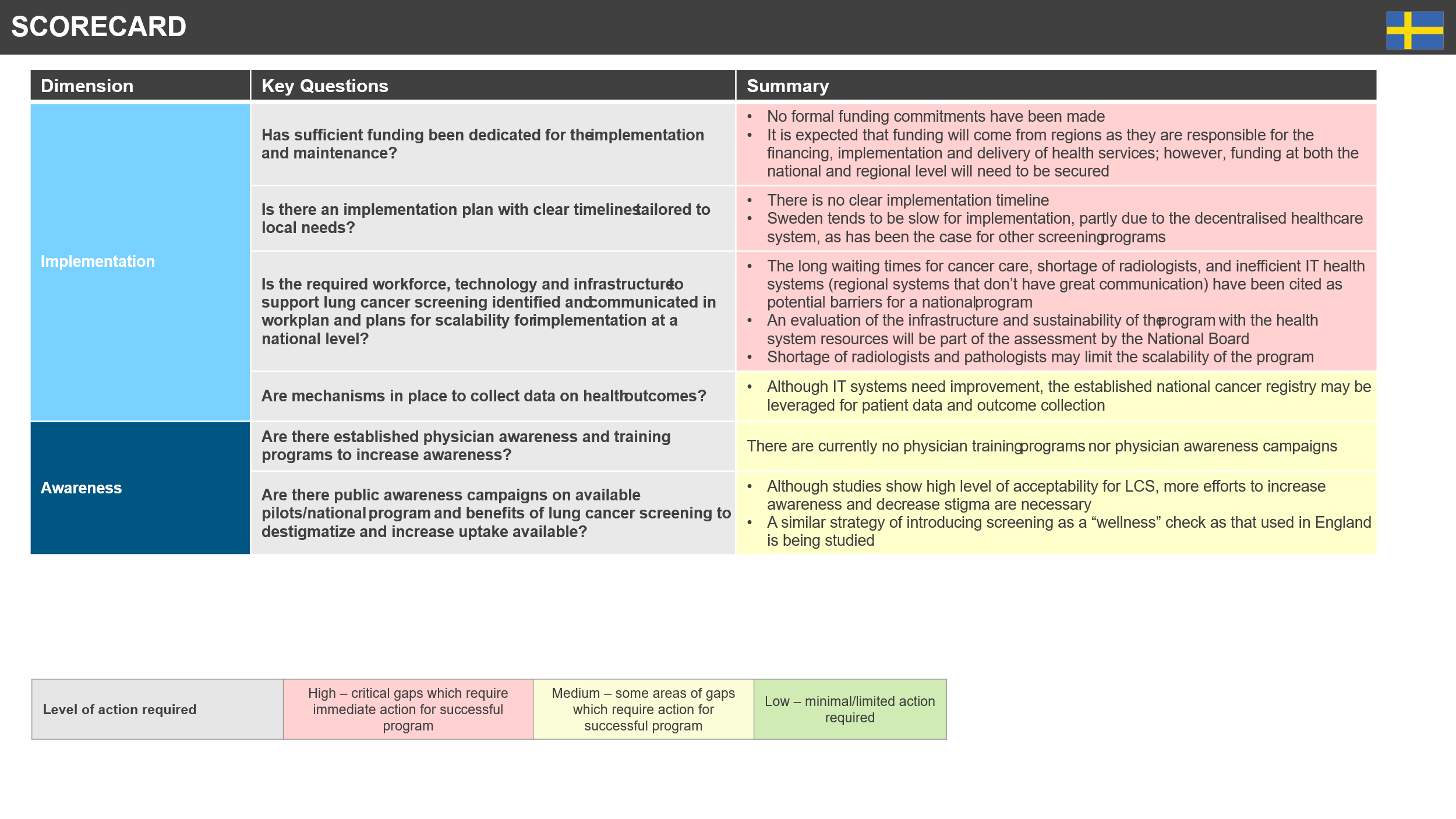
Figure 20c. Assessment for Sweden for implementation and awareness dimensions

## France


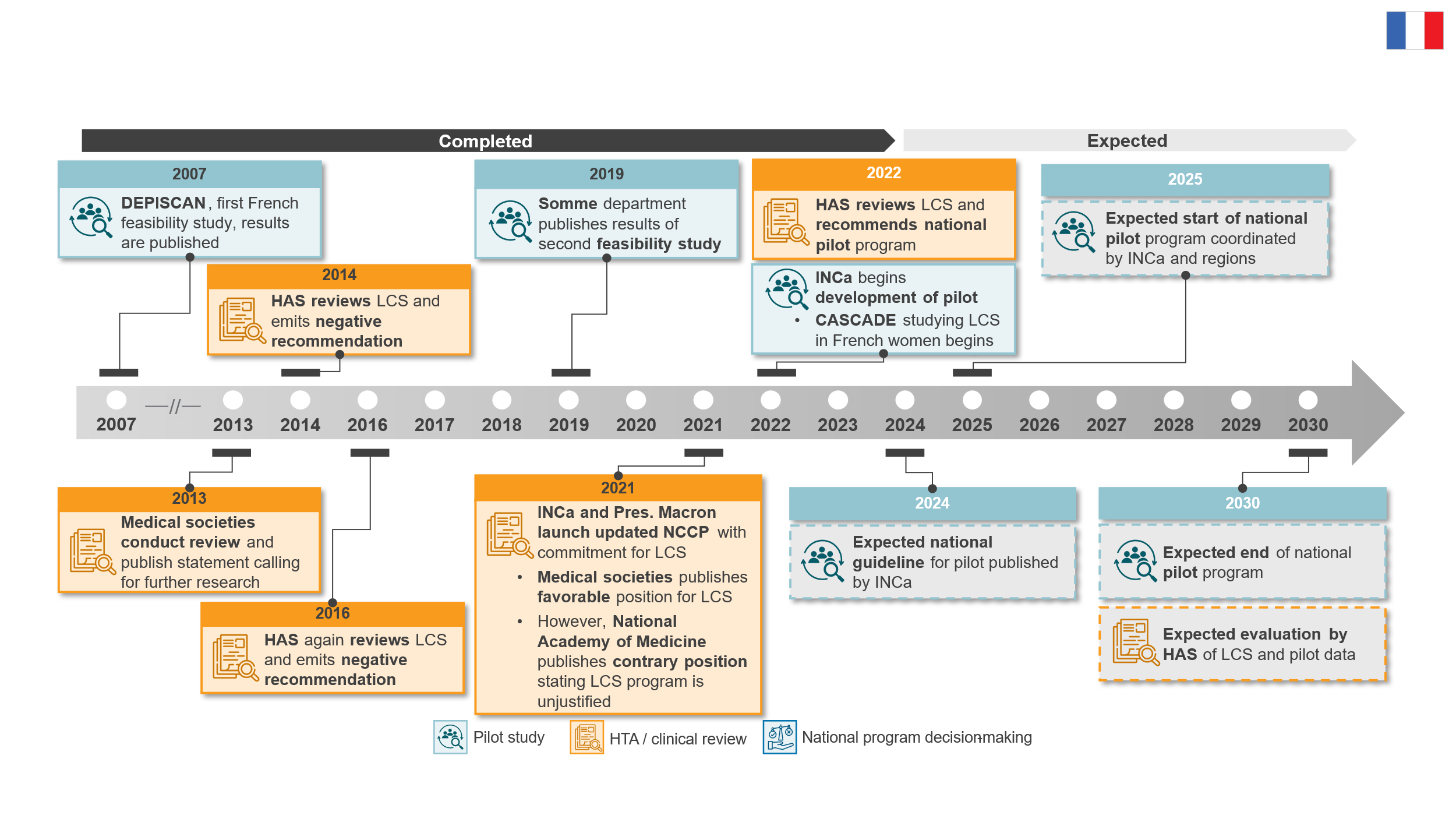
Figure 21. Timeline of key events in the implementation of a national LCS program in France


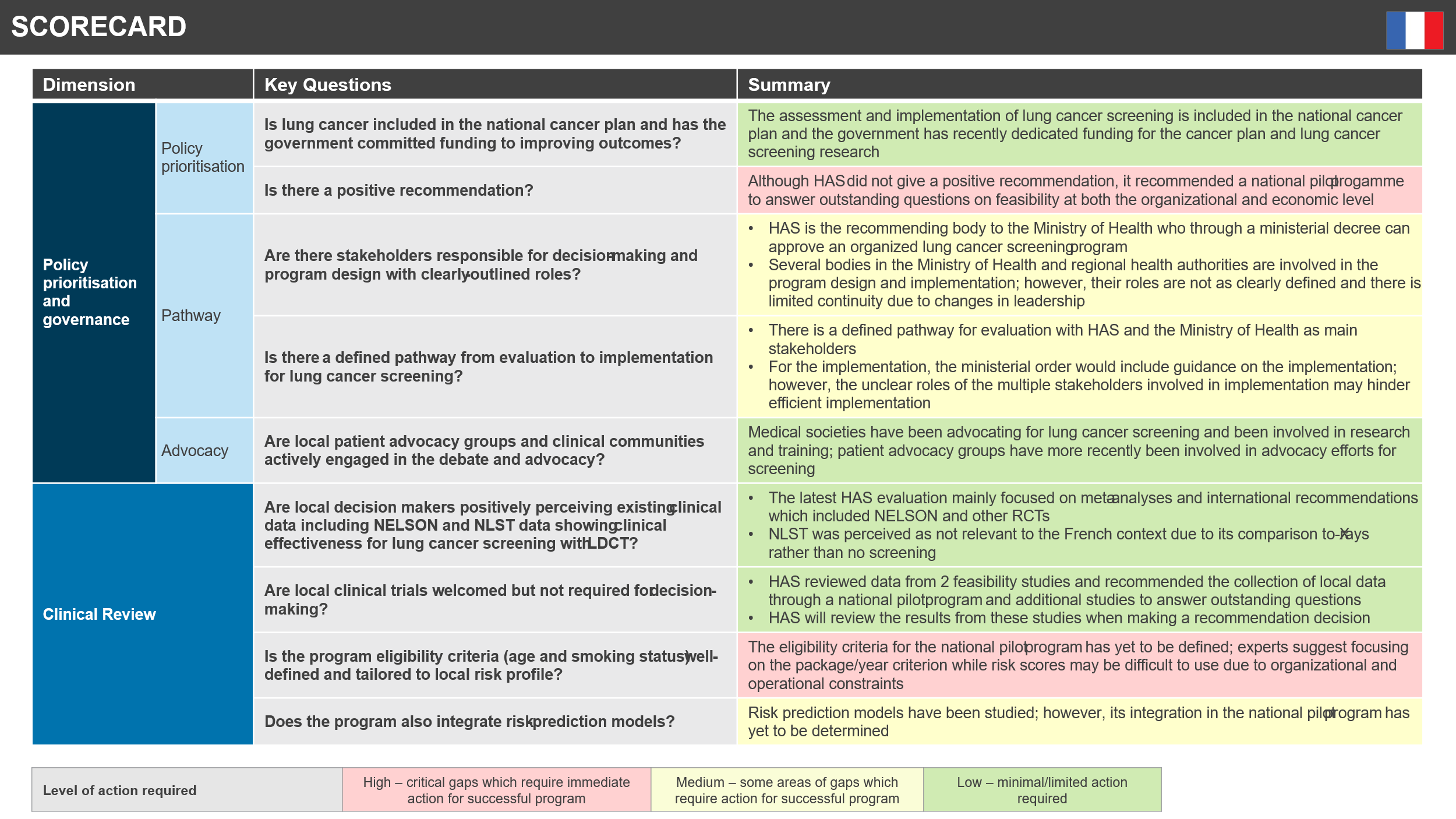
Figure 22a. Assessment for France for policy prioritisation & governance and clinical review dimensions

Figure 22b. Assessment for France for program design dimension


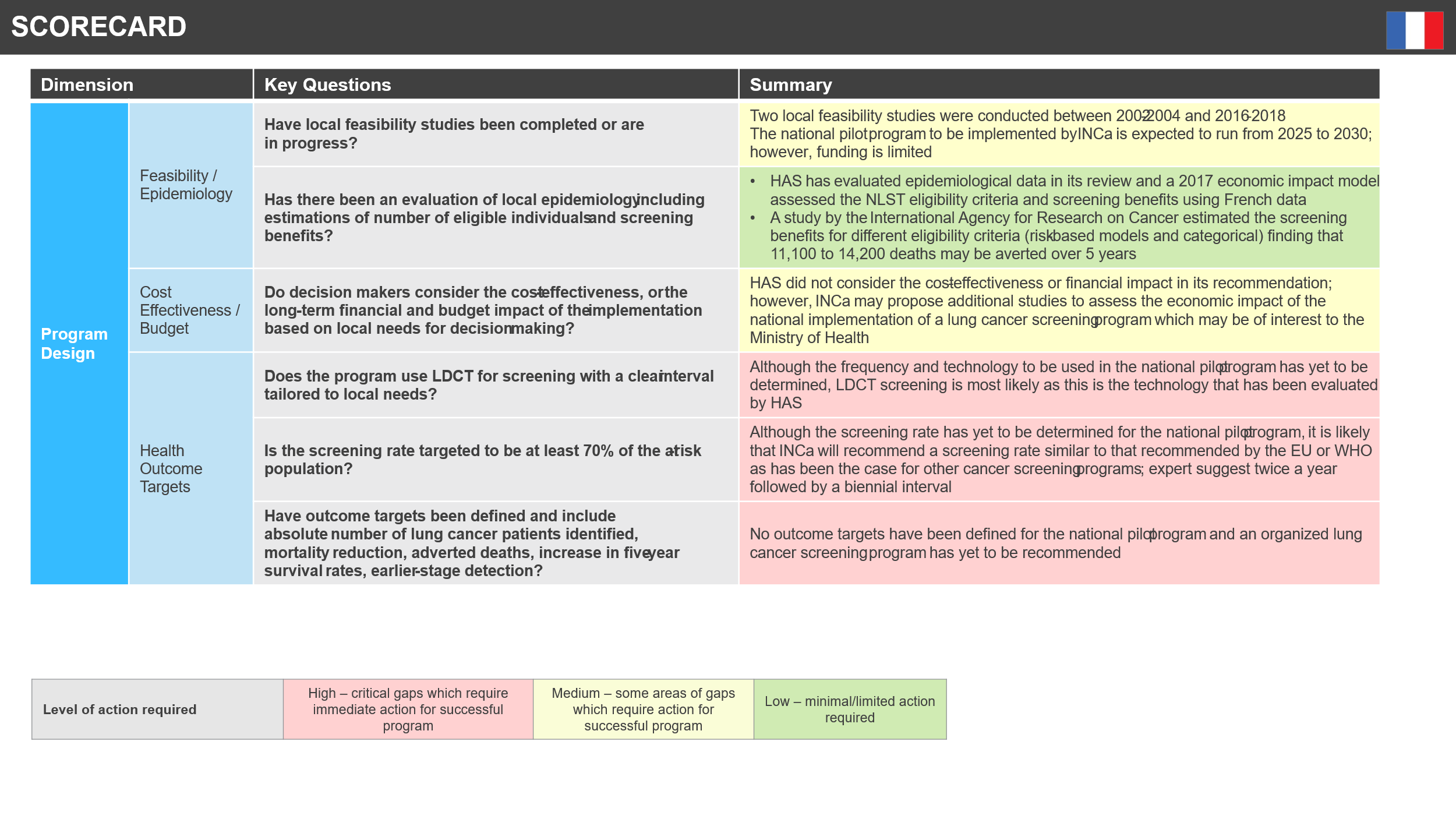


Figure 22c. Framework assessment for France for implementation and awareness dimensions


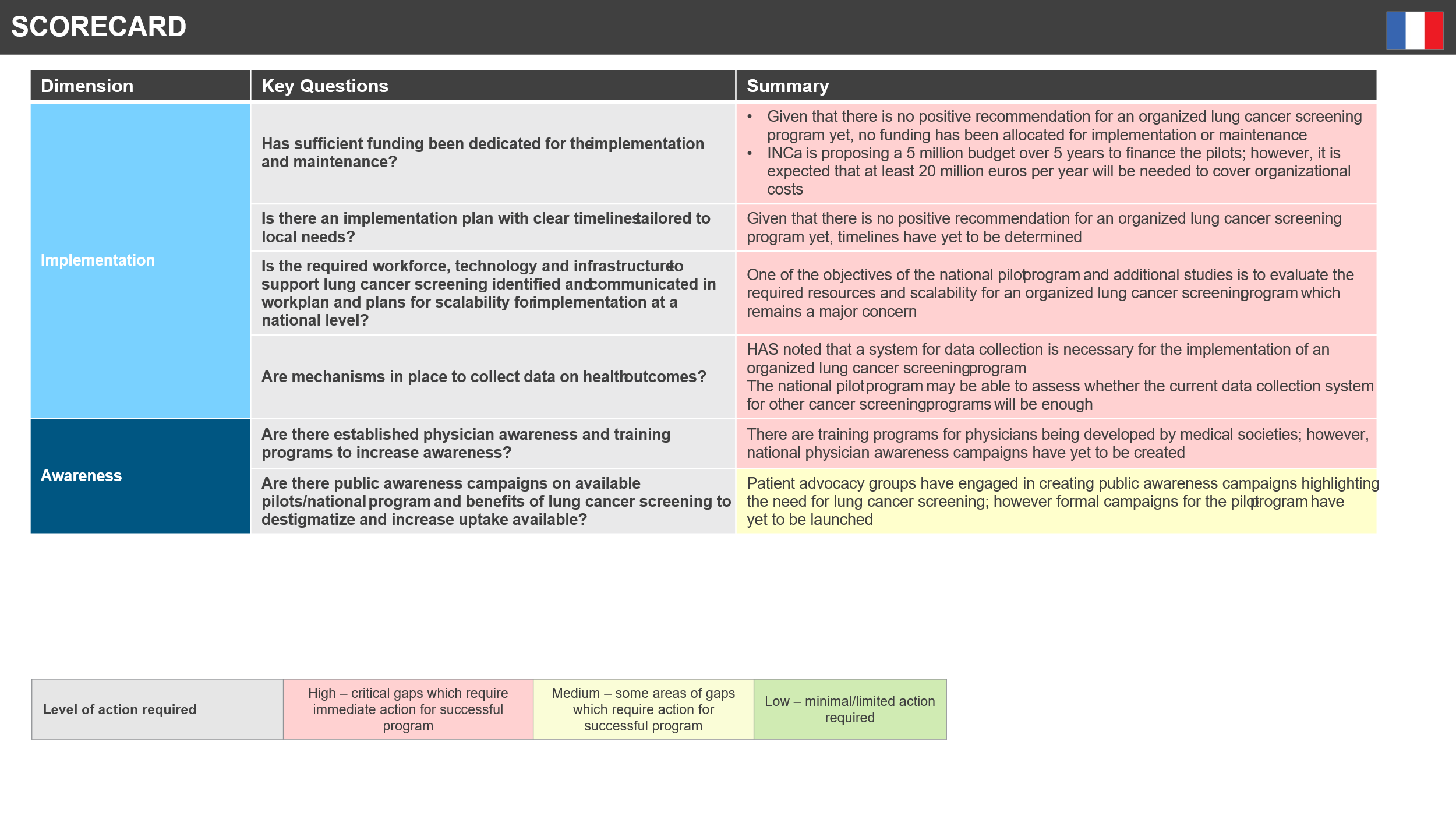


## Greece


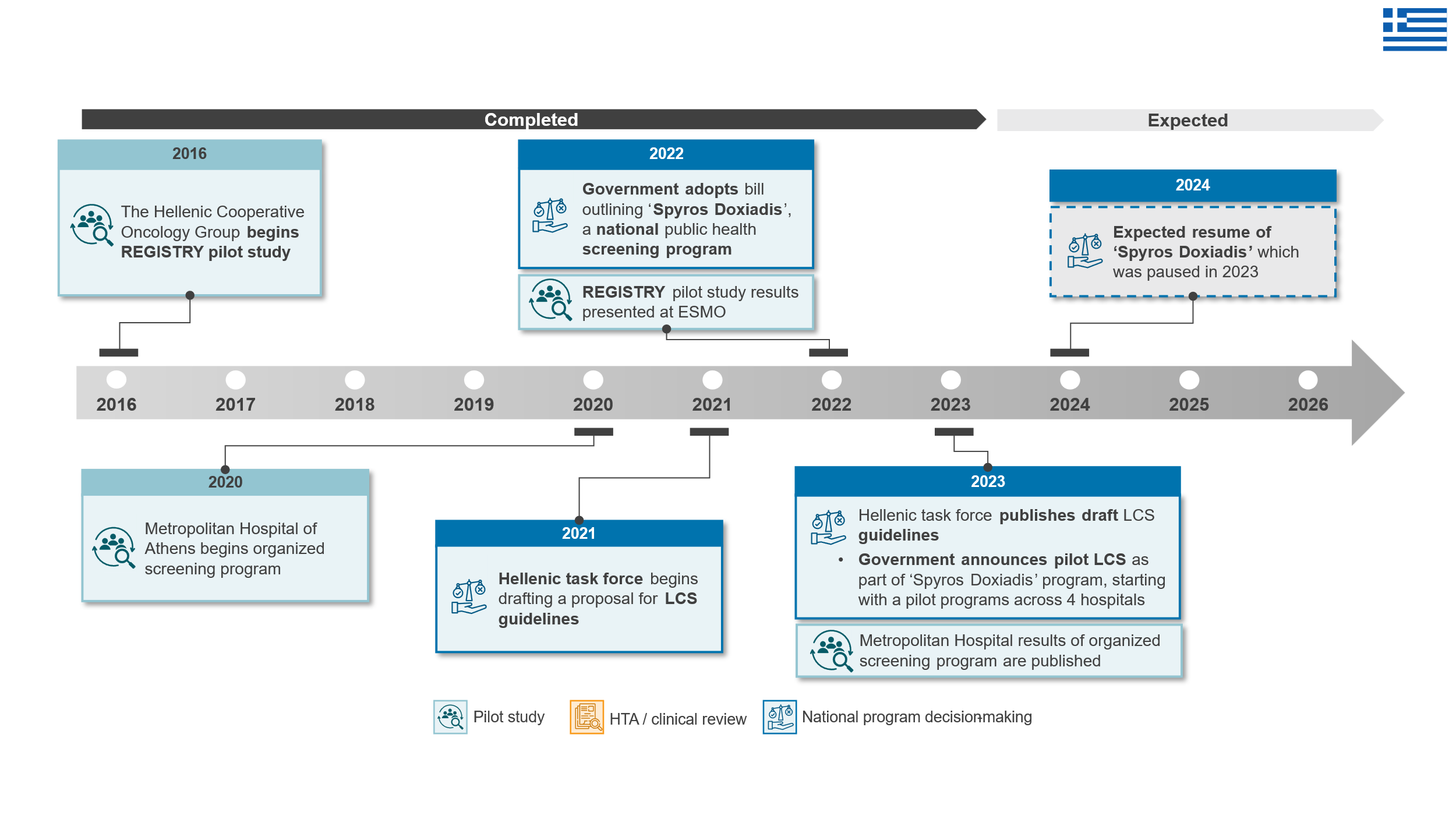
Figure 23. Timeline of key events in the implementation of a national LCS program in Greece


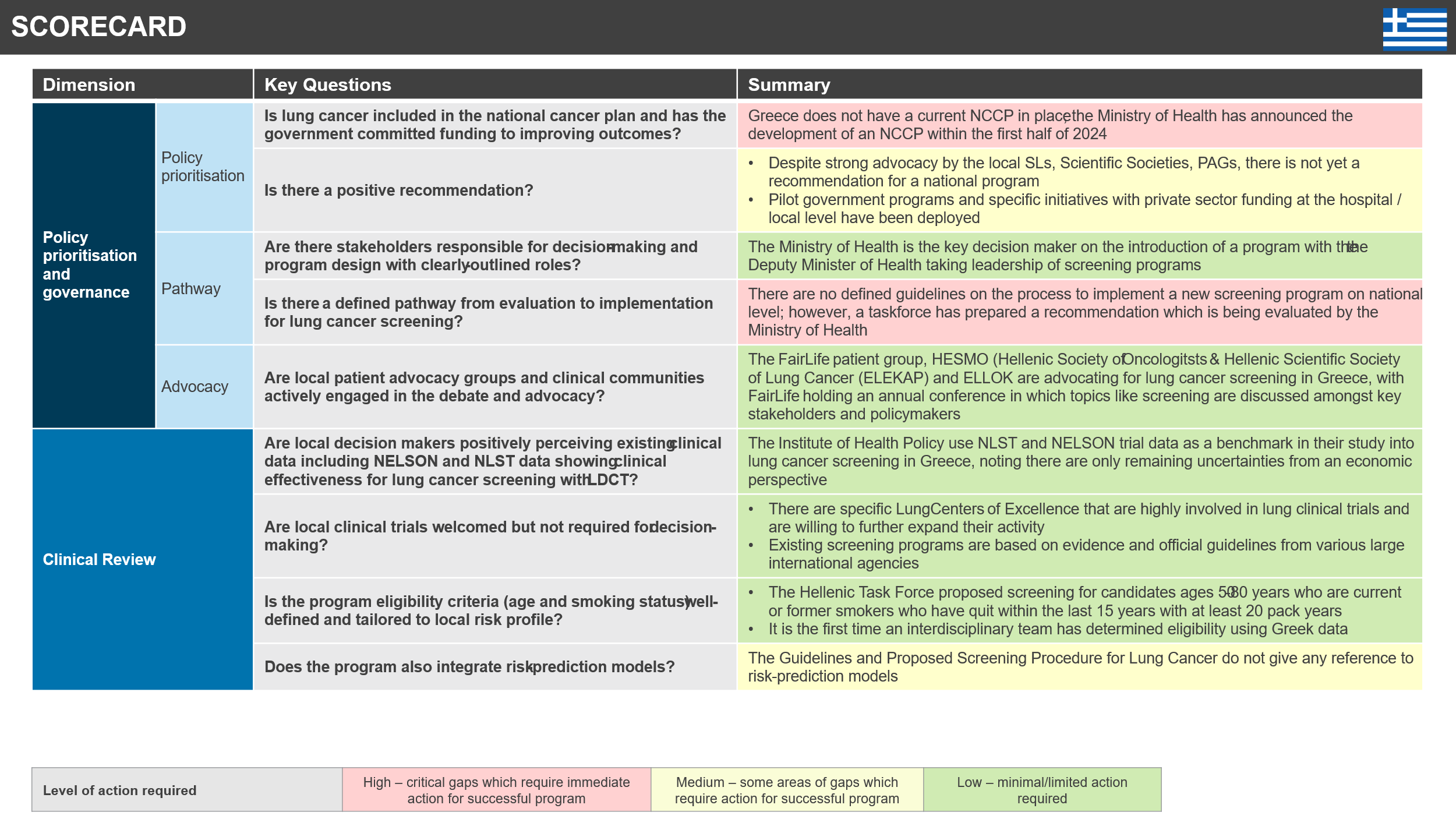
Figure 24a. Assessment for Greece for policy prioritisation & governance and clinical review dimensions

Figure 24b. Assessment for Greece for program design dimension


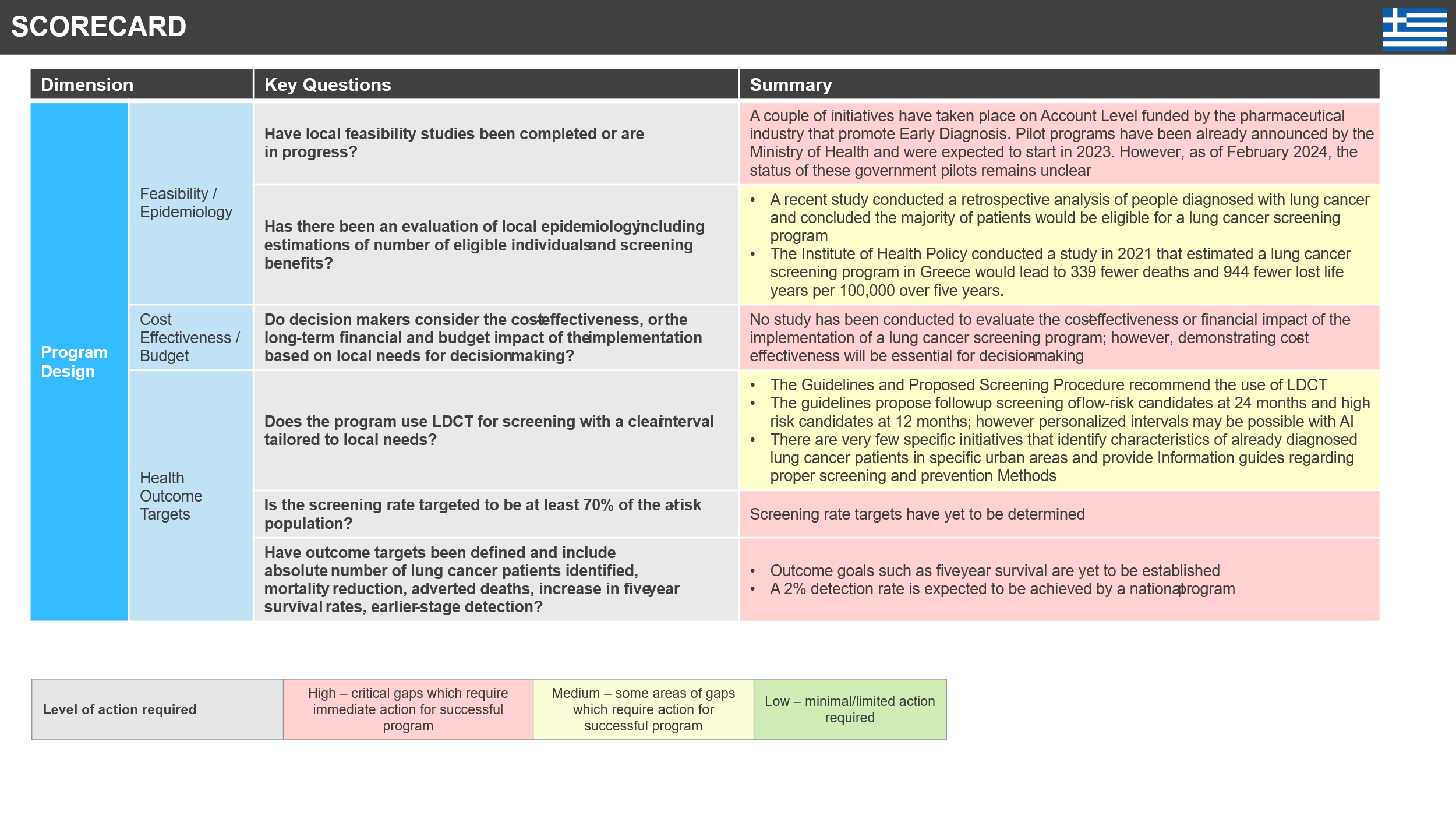


Figure 24c. Assessment for Greece for implementation and awareness dimensions


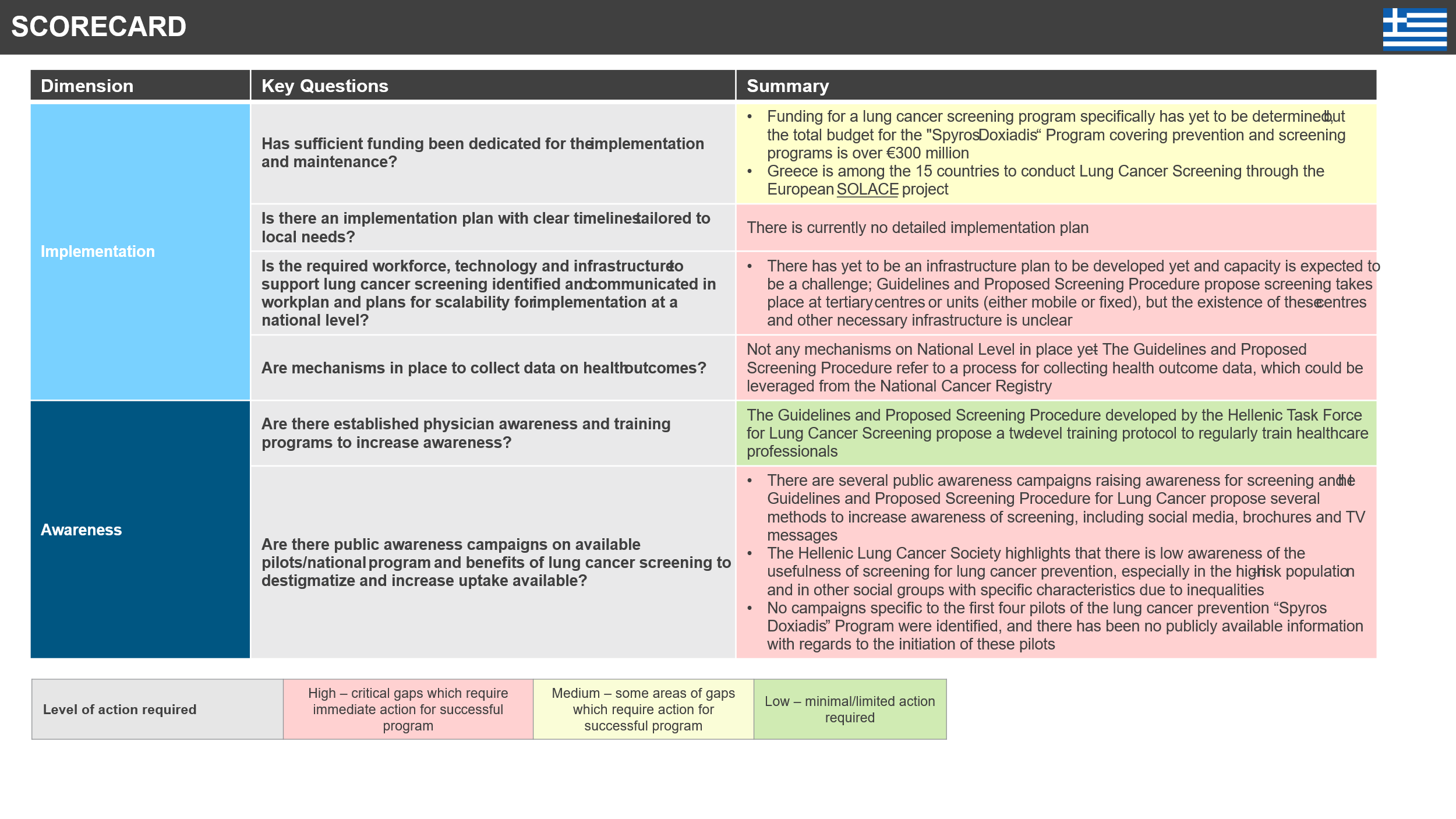


**Bibliography**

*04/07/2022 - Webinaire “Programme Pilote Dépistage Des Cancers Du Poumon Pour Fumeurs et Ex-Fumeurs,”* 2022. https://www.youtube.com/watch?v=LWaBVaNCFIE.

“44th Conference – I-ELCAP.” Accessed October 16, 2024. https://www.ielcap.org/home/ielcap/events/previous-meetings/44th-conference/.

“Adapted HTA REPORT 2014 - Screening per Il Tumore Del Polmone.” Accessed October 16, 2024. https://www.salute.gov.it/imgs/C_17_ReportDispositivi_17_0_documentoITA.pdf.

AEACAP. “AEACaP exige soluciones para afrontar el cáncer de pulmón como la nueva epidemia oculta.” AEACAP - Asociación Afectados Cáncer de Pulmón, May 25, 2023. https://afectadoscancerdepulmon.com/aeacap-exige-soluciones-para-afrontar-el-cancer-de-pulmon-como-la-nueva-epidemia-oculta/.

———. “AEACaP exige soluciones para afrontar el cáncer de pulmón como la nueva epidemia oculta.” AEACAP - Asociación Afectados Cáncer de Pulmón, May 25, 2023. https://afectadoscancerdepulmon.com/aeacap-exige-soluciones-para-afrontar-el-cancer-de-pulmon-como-la-nueva-epidemia-oculta/.

———. “AEACaP participa en el lanzamiento de Alia , una plataforma digital al servicio de pacientes y profesionales de cáncer de pulmón.” AEACAP - Asociación Afectados Cáncer de Pulmón, February 3, 2023. https://afectadoscancerdepulmon.com/aeacap-participa-en-el-lanzamiento-de-alia-una-plataforma-digital-al-servicio-de-pacientes-y-profesionales-de-cancer-de-pulmon/.

Arnold, Henry. “Lung Cancer Screening in Poland.” The Lung Cancer Policy Network, November 28, 2022. https://www.lungcancerpolicynetwork.com/lung-cancer-screening-in-poland/.

“Belgium National Cancer Plan.” Accessed October 15, 2024. https://www.iccp-portal.org/system/files/plans/Belgium_National_Cancer_Plan_2008-2010_English.pdf.

Berges, Cristina Armunia. “Se pone en marcha Cassandra, un proyecto piloto de cribado de cáncer de pulmón.” *Gaceta Médica* (blog), November 16, 2023. https://gacetamedica.com/investigacion/se-pone-en-marcha-cassandra-un-proyecto-piloto-de-cribado-de-cancer-de-pulmon/.

“Breathing a New Era: A Comparative Analysis of Lung Cancer Policies across Europe.” Accessed October 15, 2024. https://www.eiu.com/graphics/marketing/pdf/Lung-Cancer%20in-Europe-EIU-2019-9-10-final.pdf.

Bundesumweltministeriums. “Bundesumweltministerium lässt künftig Lungenkrebsfrüherkennung mittels Niedrigdosis-Computertomographie zu- BMUV - Pressemitteilung.” Bundesministerium für Umwelt, Naturschutz, nukleare Sicherheit und Verbraucherschutz, February 28, 2024. https://www.bmuv.de/PM11003.

———. “Referentenentwurf einer Verordnung über die Zulässigkeit der Anwendung der Niedrigdosis-Computertomographie zur Früherkennung von Lungenkrebs bei Rauchern (Lungenkrebs-Früherkennungs-Verordnung)- BMUV - Gesetze und Verordnungen.” bmuv.de, June 21, 2024. https://www.bmuv.de/GE1017.

“Considerations to Ensure Optimum Roll-out of Targeted Lung Cancer Screening over the next Five Years.” British Society of Thoracic Imaging and The Royal College of Radiologists, n.d. https://www.rcr.ac.uk/sites/default/files/final_pdf_considerations_to_ensure_optimum_roll-out_of_targeted_lung_cancer_screening.pdf.

Consilium. “Council Updates Its Recommendation to Screen for Cancer.” Accessed October 16, 2024. https://www.consilium.europa.eu/en/press/press-releases/2022/12/09/council-updates-its-recommendation-to-screen-for-cancer/.

“Cribado Del Cáncer de Pulmón Mediante TC de Baja Dosis - Proyecto Piloto Nacional - Documento de Actuacion.” Accessed October 15, 2024. https://sect.es/images/site/boletines/2022/enero/img/Documento_resumen.pdf.

“Croatia National Cancer Control Plan 2020 – 2030.” Accessed October 15, 2024. https://www.iccp-portal.org/system/files/plans/NPPR_ENG_final.pdf.

“Croatia National Protocol - NACIONALNI PROGRAM PREVENCIJE RAKA PLUĆA.” Accessed October 15, 2024. https://zdravlje.gov.hr/UserDocsImages/2019%20Programi%20i%20projekti/NACIONALNI%20PROGRAM%20PREVENCIJE%20RAKA%20PLU%C4%86A.pdf.

Deanna. “ALCASE Italia.” ALCASE Italia. Accessed October 16, 2024. https://alcase.it/.

“Dépistage Du Cancer Du Poumon : Lancement d’un Projet Pilote Par l’Institut - Actualités.” Accessed October 16, 2024. https://www.e-cancer.fr/Actualites-et-evenements/Actualites/Depistage-du-cancer-du-poumon-lancement-d-un-projet-pilote-par-l-Institut.

Desimpel, Fabian, Janis Luyten, Cécile Camberlin, Célia Primus-de Jong, Leen Verleye, and Mattias Neyt. *Lung Cancer Screening in a High-Risk Population*. 1st ed. KCE Reports - Health Technology Assessment (HTA). BE: Belgian Health Care Knowledge Centre (KCE), 2024. https://doi.org/10.57598/R379C.

“ESMO Congress 2022 | OncologyPRO.” Accessed October 16, 2024. https://oncologypro.esmo.org/meeting-resources/esmo-congress-2022/a-pilot-study-of-a-lung-cancer-screening-program-with-low-dose-computed-tomography-in-high-risk-individuals-in-greece.

“Estrategia En Cáncer Del Sistema Nacional de Salud.” Accessed October 15, 2024. https://www.sanidad.gob.es/areas/calidadAsistencial/estrategias/cancer/docs/ESTRATEGIA_EN_CANCER_DEL_SNS.pdf.

e.V, Deutsche Röntgengesellschaft. “Durchbruch Auf Dem Weg Zu Einem Früherkennungsprogramm Für Lungenkrebs | DRG.De,” n.d. https://www.drg.de.

Expert interview with Dr. Annemiek Snoeckx (Belgium). Moderated by CRA, Personal communication.

Expert interview with Dr. David Baldwin (England). Moderated by CRA, Personal communication.

Expert interview with Dr. David Baldwin (England). Moderated by CRA, Personal communication.

Expert interview with Dr. Eugenio Paci (Italy). Moderated by CRA, Personal communication.

Expert interview with Dr. Juan Carlos Trujillo (Spain). Moderated by CRA, Personal communication.

Expert interview with Dr. Sebastien Couraud (France). Moderated by CRA, Personal communication.

Expert interview with Dr. Sophia Lampaki (Greece). Moderated by CRA, Personal communication.

Expert interview with Mr. Ivica Belina (Croatia). Moderated by CRA, Personal communication.

Expert interview with Mr. Sebastien Schmidt (Germany). Moderated by CRA, Personal communication.

Expert interview with Ms. Ebba Hallersjö (Sweden). Moderated by CRA, Personal communication.

FairLife L.C.C. “Lung Cancer: 25% Mortality Reduction by Screening Implementation FairLife LCC’s Online Conference: «Lung Cancer – Prevention and Early Diagnosis: Lung Cancer Screening Programme in Greece, Are We Ready? »,” November 25, 2021. https://fairlifelcc.com/en/lung-cancer-25-mortality-reduction-by-screening-implementation-fairlife-lccs-online-conference-lung-cancer-prevention-and-early-diagnosis-lung-cancer-screening-programme-2/.

FairLife L.C.C. “Our Vision.” Accessed October 16, 2024. https://fairlifelcc.com/en/about/our-vision/.

FairLife L.C.C. “Πρόγραμμα - Ο Καρκίνος του Πνεύμονα σε Πρώτο Πλάνο.” Accessed October 16, 2024. https://fairlifelcc.com/o-karkinos-toy-pneymona-se-proto-plano/programma/.

Feng. “A Modeling Study of Eligibility Criteria for National Lung Cancer Screening in France,” n.d.

“Flagship Initiatives - European Commission,” October 15, 2024. https://health.ec.europa.eu/non-communicable-diseases/cancer/flagship-initiatives_en.

“Game Changers IPSOS.” Rookenquete, 2020. https://www.kanker.be/sites/default/files/rapport_2020.pdf.

Garcia Perez, Lidia. “Evaluación del programa de cribado de cáncer de pulmón.” *Ministerio de Sanidad, Santa Cruz de Tenerife: Servicio Canario de la Salud, Santiago de Compostela: Agencia Gallega para la Gestión del Conocimiento en Salud, ACIS*, n.d.

GOV.UK. “New Lung Cancer Screening Roll out to Detect Cancer Sooner,” n.d. https://www.gov.uk/government/news/new-lung-cancer-screening-roll-out-to-detect-cancer-sooner.

“Greece Lung Cancer Screening Medical Societies Recommendations - ΠΡΟΤΕΙΝΟΜΕΝΗ ΔΙΑΔΙΚΑΣΙΑ ΠΡΟΣΥΜΠΤΩΜΑΤΙΚΟΥ ΕΛΕΓΧΟΥ ΚΑΡΚΙΝΟΥ ΠΝΕΥΜΟΝΑ (ΠΕΚΠ).” Accessed October 15, 2024. https://ellok.org/wp-content/uploads/2023/11/%CE%A0%CE%A1%CE%9F%CE%A4%CE%95%CE%99%CE%9D%CE%9F%CE%9C%CE%95%CE%9D%CE%97-%CE%94%CE%99%CE%91%CE%94%CE%99%CE%9A%CE%91%CE%A3%CE%99%CE%91-%CE%A0%CE%95%CE%9A%CE%A0-19.04.2023.pdf.

“Greece National Cancer Plan - ΕΘΝΙΚΟ ΣΧΕΔΙΟ ΔΡΑΣΗΣ ΓΙΑ ΤΟΝ ΚΑΡΚΙΝΟ 2011-2015.” Accessed October 16, 2024. https://www.anti-cancer.gr/catalogue/SxedioDrasis_24selido.pdf.

Haute Autorité de Santé. “Dépistage du cancer bronchopulmonaire par scanner thoracique faible dose sans injection : actualisation de l’avis de 2016,” n.d. https://www.has-sante.fr/jcms/p_3310940/fr/depistage-du-cancer-bronchopulmonaire-par-scanner-thoracique-faible-dose-sans-injection-actualisation-de-l-avis-de-2016.

HealthReport.gr. “Δωρεάν Προληπτικές Εξετάσεις: Ξεκινούν Από Το Νέο Έτος Για Τον Καρκίνο Πνεύμονα Σε 4 Νοσοκομεία.” Accessed November 11, 2024. https://www.healthreport.gr/dorean-proliptikes-exetaseis-xekinoyn-apo-to-neo-etos-gia-ton-karkino-pneymona-se-4-nosokomeia/.

Iatropedia. “Πρόγραμμα ‘Σπύρος Δοξιάδης’: Ποιοι Πολίτες Δικαιούνται Δωρεάν Προληπτικές Εξετάσεις Τους Επόμενους Μήνες,” January 22, 2020. https://www.iatropedia.gr/eidiseis/programma-spyros-doksiadis-poioi-polites-dikaiountai-dorean-proliptikes-eksetaseis-tous-epomenous-mines/126246/.

“Initiation of the Consultation Procedure: Evaluation of Lung Cancer Early Detection Using Low-Dose Computed Tomography in Smokers - Federal Joint Committee.” Accessed October 15, 2024. https://www.g-ba.de/beschluesse/6390/.

Insider. “Πλεύρης: «Όχημα» για την καταπολέμηση του καρκίνου τα προγράμματα προληπτικού ελέγχου – Τα μεγάλα στοιχήματα,” January 23, 2023. https://www.insider.gr/politiki/260266/karkinos-ohima-gia-tin-katapolemisi-toy-ta-programmata-proliptikoy-eleghoy-ta.

IQWiG. “[S19-02] Lung Cancer Screening Using Low-Dose Computed Tomography.” Accessed October 15, 2024. https://www.iqwig.de/en/projects/s19-02.html.

Kaushal, Kanica, and Sunil K. Raina. “Biostatistics behind Risk Prediction Models.” *Journal of Family Medicine and Primary Care* 12, no. 5 (May 2023): 1016–17. https://doi.org/10.4103/jfmpc.jfmpc_2406_22.

Koning, Harry J. de, Carlijn M. van der Aalst, Pim A. de Jong, Ernst T. Scholten, Kristiaan Nackaerts, Marjolein A. Heuvelmans, Jan-Willem J. Lammers, et al. “Reduced Lung-Cancer Mortality with Volume CT Screening in a Randomized Trial.” *New England Journal of Medicine* 382, no. 6 (February 6, 2020): 503–13. https://doi.org/10.1056/NEJMoa1911793.

la Repubblica. “Tumore al polmone, è tempo di pensare a uno screening nazionale,” November 16, 2023. https://www.repubblica.it/salute/dossier/labrevolution/2023/11/16/news/tumore_polmone_screening_indagine_fumatori-420521803/.

la Repubblica. “Tumore al polmone, il programma di screening italiano fa scuola,” November 22, 2023. https://www.repubblica.it/salute/2023/11/22/news/tumore_al_polmone_il_programma_di_screening_italiano_fa_scuola-421017269/.

“La Stratégie Décennale de Lutte Contre Les Cancers 2021-2030 - Stratégie de Lutte Contre Les Cancers En France.” Accessed October 15, 2024. https://www.e-cancer.fr/Institut-national-du-cancer/Strategie-de-lutte-contre-les-cancers-en-France/La-strategie-decennale-de-lutte-contre-les-cancers-2021-2030.

La Voz de Galicia. “Galicia seleccionará con inteligencia artificial a los candidatos a un cribado de cáncer de colon y próstata,” October 14, 2023. https://www.lavozdegalicia.es/noticia/sociedad/2023/10/14/galicia-aplicara-inteligencia-artificial-cribados-cancer-pulmon/0003_202310G14P24991.htm.

“Le Mois sans Tabac Ravive Les Besoins de Scanner Low Dose Pour Le Dépistage Précoce Du Cancer Du Poumon.” Accessed October 16, 2024. https://www.thema-radiologie.fr/actualites/2815/le-mois-sans-tabac-ravive-les-besoins-de-scanner-low-dose-pour-le-depistage-precoce-du-cancer-du-poumon.html.

“Le Scanner Low Dose Recommandé Par Le Collectif Ensemble Nous Poumons.” Accessed October 16, 2024. https://www.thema-radiologie.fr/actualites/3096/le-scanner-low-dose-recommande-par-le-collectif-ensemble-nous-poumons.html.

Lederlin, Mathieu, Constance de Margerie-Mellon, Samia Boussouar, Sébastien Bommart, and Caroline Caramella. “Lung Cancer Screening: French Radiologists Should Prepare for It.” *Diagnostic and Interventional Imaging* 102, no. 4 (April 1, 2021): 197–98. https://doi.org/10.1016/j.diii.2021.02.004.

Leiter, Amanda, Rajwanth R. Veluswamy, and Juan P. Wisnivesky. “The Global Burden of Lung Cancer: Current Status and Future Trends.” *Nature Reviews Clinical Oncology* 20, no. 9 (September 2023): 624–39. https://doi.org/10.1038/s41571-023-00798-3.

“Les Sociétés Savantes Se Mobilisent à Nouveau En Faveur Du Dépistage Du Cancer Du Poumon Par Scanner Low Dose.” Accessed October 16, 2024. https://www.thema-radiologie.fr/actualites/3131/les-societes-savantes-se-mobilisent-a-nouveau-en-faveur-du-depistage-du-cancer-du-poumon-par-scanner-low-dose.html.

“Lung Ambition Alliance.” Accessed October 15, 2024. https://www.astrazeneca.es/areas-terapeuticas/oncologia/Lung_ambition_alliance.html.

“Lung Ambition Alliance.” Accessed October 16, 2024. https://www.astrazeneca.es/areas-terapeuticas/oncologia/Lung_ambition_alliance.html.

“Lung Cancer - UK National Screening Committee (UK NSC) - GOV.UK.” Accessed October 15, 2024. https://view-health-screening-recommendations.service.gov.uk/lung-cancer/.

“Lung Cancer Screening - Current Situation in Sweden - Lung & Allergy Forum.” Accessed October 15, 2024. https://etidning.slmf.se/p/lung-allergiforum/nr-4-2022-12-08/a/lungcancerscreening-aktuellt-lage-i-sverige/1915/815853/34423657.

“Lung Cancer Screening - RCC Knowledge Bank.” Accessed October 15, 2024. https://kunskapsbanken.cancercentrum.se/diagnoser/lungcancer/vardprogram/lungcancerscreening/.

“Lung Cancer Screening: 2022 Could Be a Turning Point for Europe | Cancerworld Magazine,” January 14, 2022. https://cancerworld.net/lung-cancer-screening-2022-could-be-a-turning-point-for-europe/.

Lung Check. “O projektcie.” Accessed October 15, 2024. https://www.lungcheck.pl/o-projekcie.

Lung Check. “Organizacja Programu.” Accessed October 15, 2024. https://www.lungcheck.pl/rada-programowa.

Lung Check. “Szkolenia.” Accessed October 15, 2024. https://www.lungcheck.pl/szkolenia.

“Lung Screening – from Clinical Studies to Established Programs.” Accessed October 15, 2024. https://events.siemens-healthineers.com/sessions/symposium/update-on-lung-cancer-screening-in-europe.

“Lungcancerscreening - RCC Kunskapsbanken,” n.d. https://kunskapsbanken.cancercentrum.se/diagnoser/lungcancer/vardprogram/lungcancerscreening/.

Madrid, Comunidad de. “Díaz Ayuso anuncia un programa piloto propio de cribado de cáncer de pulmón para incluirlo como prestación de cartera básica de servicios.” Comunidad de Madrid, September 25, 2023. https://www.comunidad.madrid/noticias/2023/09/25/diaz-ayuso-anuncia-programa-piloto-propio-cribado-cancer-pulmon-incluirlo-prestacion-cartera-basica-servicios.

“Manchester’s Lung Health Check Pilot.” Accessed October 15, 2024. https://mft.nhs.uk/app/uploads/sites/12/2019/02/lung-health-check-manchester-report_tcm9-309848.pdf.

Mar 2024, 4. “Mef Nilbert Leads the Update of a New Swedish Cancer Strategy | LUCC,” August 12, 2024. https://www.lucc.lu.se/article/mef-nilbert-leads-update-new-swedish-cancer-strategy.

———. “Mef Nilbert Leads the Update of a New Swedish Cancer Strategy | LUCC,” August 12, 2024. https://www.lucc.lu.se/article/mef-nilbert-leads-update-new-swedish-cancer-strategy.

Marien, Annelin, and |Annelin Marien|. “Tegen 2026 willen longartsen screening naar longkanker bij Vlaamse bevolking: ‘Vroegtijdig opsporen zorgt voor kwart minder sterfgevallen.’” hln.be, August 18, 2022. https://www.hln.be/medisch/tegen-2026-willen-longartsen-screening-naar-longkanker-bij-vlaamse-bevolking-vroegtijdig-opsporen-zorgt-voor-kwart-minder-sterfgevallen~a8751d0e/.

“Ministerio de Sanidad - Áreas - Cartera de Servicios Complementaria de Las Comunidades Autónomas y de Las Mutualidades de Funcionarios.” Accessed October 15, 2024. https://www.sanidad.gob.es/profesionales/prestacionesSanitarias/CarteraDeServicios/ComplemenariaCS/CS-Complementaria.htm.

“MInistero Della Salute - Piano Oncologico Nazionale: Documento Di Pianificazione e Indirizzo per La Prevenzione e Il Contrasto Del Cancro 2023-2027.” Accessed October 15, 2024. https://www.salute.gov.it/imgs/C_17_pubblicazioni_3291_allegato.pdf.

Ministerstwo Zdrowia. “Narodowa Strategia Onkologiczna - Ministerstwo Zdrowia - Portal Gov.pl,” n.d. https://www.gov.pl/web/zdrowie/narodowa-strategia-onkologiczna-nso.

“NHS England » NHS Launches Awareness Campaign for England’s Most Deadly Cancer.” Accessed October 15, 2024. https://www.england.nhs.uk/2022/08/nhs-launches-awareness-campaign-for-englands-most-deadly-cancer/.

Ocak, Sebahat, Kurt Tournoy, Thierry Berghmans, Ingel Demedts, Rodolphe Durieux, Annelies Janssens, Luigi Moretti, et al. “Lung Cancer in Belgium.” *Journal of Thoracic Oncology* 16, no. 10 (October 1, 2021): 1610–21. https://doi.org/10.1016/j.jtho.2021.07.022.

O’Dowd, Emma L, Richard W Lee, Ahsan R Akram, Emily C Bartlett, Stephen H Bradley, Kate Brain, Matthew E J Callister, et al. “Defining the Road Map to a UK National Lung Cancer Screening Programme.” *The Lancet Oncology* 24, no. 5 (May 2023): e207–18. https://doi.org/10.1016/S1470-2045(23)00104-3.

OECD. *EU Country Cancer Profile: Belgium 2023*. EU Country Cancer Profiles. OECD, 2023. https://doi.org/10.1787/9a976db3-en.

———. *EU Country Cancer Profile: Croatia 2023*. EU Country Cancer Profiles. OECD, 2023. https://doi.org/10.1787/372db8b8-en.

———. *EU Country Cancer Profile: Greece 2023*. EU Country Cancer Profiles. OECD, 2023. https://doi.org/10.1787/30b7e1f9-en.

Ops, Dev. “Lung Cancer Screening in Poland Pilot Program Is Restarted After COVID-19 Lockdown.” *ILCN.Org (ILCN/WCLC)* (blog), January 21, 2021. https://www.ilcn.org/lung-cancer-screening-in-poland-pilot-program-is-restarted-after-covid-19-lockdown/.

Org_SG. “Lungenkrebsfrüherkennung mittels Niedrigdosis-Computertomographie - Wissenschaftliche Bewertung des Bundesamtes für Strahlenschutz gemäß § 84 Absatz 3 Strahlenschutzgesetz.” Bundesamt für Strahlenschutz (BfS), August 20, 2021. https://doris.bfs.de/jspui/handle/urn:nbn:de:0221-2021082028027.

“Our Role in Lung Health Checks - Roy Castle Lung Cancer Foundation,” July 27, 2021. https://roycastle.org/lung-health-checks/our-role-in-lung-health-checks/.

Pelekanaki, Marianthi. “Greek Cancer Plan to Be Tabled Soon amid Concerning Mortality Rates.” www.euractiv.com, December 18, 2023. https://www.euractiv.com/section/health-consumers/news/greek-cancer-plan-to-be-tabled-soon-amid-concerning-mortality-rates/.

“Poland Lung Cancer Screening Protocol - Ogólnopolski Program Wczesnego Wykrywania Raka Płuca (WWRP) Za Pomocą Niskodawkowej Tomografii Komputerowej (NDTK) – Połączenie Prewencji Wtórnej z Pierwotną w Celu Poprawy Świadomości Dotyczącej Raka Płuca Wśród Społeczeństwa i Personelu Ochrony Zdrowia.” Accessed October 15, 2024. https://www.power.gov.pl/media/72320/Zalacznik_17_Ogolnopolski_Program_WWRP.pdf.

Poon, Charlotte, Artes Haderi, Alexander Roediger, and Megan Yuan. “Should We Screen for Lung Cancer? A 10-Country Analysis Identifying Key Decision-Making Factors.” *Health Policy* 126, no. 9 (September 2022): 879–88. https://doi.org/10.1016/j.healthpol.2022.06.003.

Poon, Charlotte, Tim Wilsdon, Iqra Sarwar, Alexander Roediger, and Megan Yuan. “Why Is the Screening Rate in Lung Cancer Still Low? A Seven-Country Analysis of the Factors Affecting Adoption.” *Frontiers in Public Health* 11 (November 9, 2023): 1264342. https://doi.org/10.3389/fpubh.2023.1264342.

“¿Por Qué No Se Implanta El Cribado de Cáncer de Pulmón En España? | Salud.” Accessed October 15, 2024. https://www.elmundo.es/ciencia-y-salud/salud/2023/09/22/650c773921efa0b9398b457c.html.

“Progetto Pilota Di Un Programma Di Screening per Il Tumore Polmonare Integrato Con La Cessazione Del Fumo: Percorsi, Selezione Dei Soggetti e Protocolli Diagnostici, in Vista Di Una Valutazione HTA | CCM - Network.” Accessed January 15, 2025. https://www.ccm-network.it/progetto.jsp?id=node/2046&idP=740.

“Programma R.I.S.P. - Rete Italiana Screening Polmonare.” Accessed October 16, 2024. https://programmarisp.it/.

“Programme Pilote Du Dépistage Des Cancers Du Poumon : L’Institut National Du Cancer Publie l’appel à Candidatures - Dossiers et Communiqués de Presse.” Accessed October 15, 2024. https://www.e-cancer.fr/Presse/Dossiers-et-communiques-de-presse/Programme-pilote-du-depistage-des-cancers-du-poumon-l-Institut-national-du-cancer-publie-l-appel-a-candidatures.

ProtoThema. “Ασημένια διάκριση για το Πρόγραμμα Προληπτικού Ελέγχου «Πνευμόνων Υγεία» για τον καρκίνο του πνεύμονα στο Metropolitan Hospital,” July 7, 2022. https://www.protothema.gr/ugeia/article/1262344/asimenia-diakrisi-programma-proliptikou-eleghou-pneumonon-ugeia-karkino-tou-pneumona-sto-metropolitan-hospital/.

Radosavljevic, Zoran. “Croatian MEP: Public Health System Facing Big Cancer Crisis.” www.euractiv.com, May 3, 2023. https://www.euractiv.com/section/diabetes-cancer-hepatitis/news/croatian-mep-public-health-system-facing-big-cancer-crisis/.

“Rete italiana screening polmonare | cancro del polmone | alcase.eu.” Accessed October 16, 2024. https://www.alcase.eu/home/rete-italiana-screening-polmonare/.

Revel, Marie-Pierre, Hendy Abdoul, Guillaume Chassagnon, Emma Canniff, Isabelle Durand-Zaleski, and Marie Wislez. “Lung CAncer SCreening in French Women Using Low-Dose CT and Artificial Intelligence for DEtection: The CASCADE Study Protocol.” *BMJ Open* 12, no. 12 (December 8, 2022): e067263. https://doi.org/10.1136/bmjopen-2022-067263.

“Risk-Model – The Roy Castle Lung Cancer Research Program.” Accessed October 16, 2024. https://liverpoollungproject.org.uk/risk-model/.

“RISP - Progetto Della Rete Italiana Screening-Polmonare.Pdf.” Accessed October 15, 2024. https://www.myecole.it/ooc/wp-content/uploads/2021/02/Progetto-della-Rete-Italiana-Screening-Polmonare.pdf.

Rzyman, Witold, Joanna Didkowska, Robert Dziedzic, Tomasz Grodzki, Tadeusz Orłowski, Edyta Szurowska, Renata Langfort, et al. “Consensus Statement on a Screening Programme for the Detection of Early Lung Cancer in Poland.” *Advances in Respiratory Medicine* 86, no. 1 (2018): 53–74. https://doi.org/10.5603/ARM.2018.0009.

“Screening - RCC.” Accessed October 15, 2024. https://www.cancercentrum.se/samverkan/regional-cancer-centres/screening/.

Silva, Mario, Giulia Picozzi, Nicola Sverzellati, Sandra Anglesio, Maurizio Bartolucci, Edoardo Cavigli, Annalisa Deliperi, et al. “Low-Dose CT for Lung Cancer Screening: Position Paper from the Italian College of Thoracic Radiology.” *La Radiologia Medica* 127, no. 5 (May 1, 2022): 543–59. https://doi.org/10.1007/s11547-022-01471-y.

Souliotis, Kyriakos, Christina Golna, Pavlos Golnas, Ioannis-Anestis Markakis, Helena Linardou, Dimitra Sifaki-Pistolla, and Evi Hatziandreou. “Lung Cancer Screening in Greece: A Modelling Study to Estimate the Impact on Lung Cancer Life Years.” *Cancers* 14, no. 22 (November 8, 2022): 5484. https://doi.org/10.3390/cancers14225484.

SPF Santé publique. “Cancer,” November 29, 2016. https://www.health.belgium.be/fr/cancer.

“Standard Protocol Prepared for the Targeted Lung Health Checks Programme.” Accessed October 15, 2024. https://www.england.nhs.uk/wp-content/uploads/2019/02/B1646-standard-protocol-targeted-lung-health-checks-programme-v2.pdf.

“State of Health in the EU Croatia Country Health Profile 2021.” Accessed October 15, 2024. https://health.ec.europa.eu/system/files/2021-12/2021_chp_hr_english.pdf.

Svenska Lungcancerstudiegruppen. “Svenska Lungcancerstudiegruppens insamlingsstiftelse.” Accessed October 16, 2024. https://slusg.org/om-oss/.

The British Medical Association is the trade union and professional body for doctors in the UK. “Health Funding Data Analysis.” Accessed October 15, 2024. https://www.bma.org.uk/advice-and-support/nhs-delivery-and-workforce/funding/health-funding-data-analysis.

“The NHS Long Term Plan.” Accessed October 15, 2024. https://www.longtermplan.nhs.uk/wp-content/uploads/2019/08/nhs-long-term-plan-version-1.2.pdf.

The PLOS Medicine Staff. “Correction: Evaluation of the Lung Cancer Risks at Which to Screen Ever- and Never-Smokers: Screening Rules Applied to the PLCO and NLST Cohorts.” *PLOS Medicine* 12, no. 1 (January 28, 2015): e1001787. https://doi.org/10.1371/journal.pmed.1001787.

Trysell, Katrin. “Lungcancerscreening: Snart Går Startskottet i Stockholm.” *Läkartidningen* (blog), May 11, 2022. https://lakartidningen.se/aktuellt/nyheter/2022/05/lungcancerscreening-snart-gar-startskottet-i-stockholm/.

———. “Socialstyrelsen: För tidigt att ta ställning till screening.” *Läkartidningen* (blog), May 11, 2022. https://lakartidningen.se/aktuellt/nyheter/2022/05/socialstyrelsen-for-tidigt-att-ta-stallning/.

“Une nouvelle stratégie nationale pour faire reculer le cancer en France.” February 4, 2021. https://www.lemonde.fr/planete/article/2021/02/04/une-nouvelle-strategie-nationale-pour-faire-reculer-le-cancer_6068728_3244.html.

University Hospital, Antwerp. “Feasibility Study of Lung Cancer Screening in the Flemish Region, the ZORALCS Study.” Clinical trial registration. clinicaltrials.gov, February 27, 2024. https://clinicaltrials.gov/study/NCT06293833.

Veronesi, Giulia, Niccolò Navone, Pierluigi Novellis, Elisa Dieci, Luca Toschi, Laura Velutti, Michela Solinas, Elena Vanni, Marco Alloisio, and Simone Ghislandi. “Favorable Incremental Cost-Effectiveness Ratio for Lung Cancer Screening in Italy.” *Lung Cancer* 143 (May 1, 2020): 73–79. https://doi.org/10.1016/j.lungcan.2020.03.015.

Vogel-Claussen, J., B. Bollmann, K. May, S. Stiebeler, S. Dettmer, A. Faron, A. Kuhlmann, et al. “MA18.05 Effectiveness of NELSON vs PLCOm2012 Lung Cancer Screening Eligibility Criteria: Final Analysis of the Prospective German HANSE Study.” *Journal of Thoracic Oncology* 19, no. 10 (October 2024): S129–30. https://doi.org/10.1016/j.jtho.2024.09.233.

Vogel-Claussen, Jens, Torsten Gerriet Blum, Stefan Andreas, Torsten T. Bauer, Jörg Barkhausen, Volker Harth, Hans-Ulrich Kauczor, et al. “Positionspapier zur Implementierung eines nationalen organisierten Programms in Deutschland zur Früherkennung von Lungenkrebs in Risikopopulationen mittels Low-dose-CT-Screening inklusive Management von abklärungsbedürftigen Screeningbefunden.” *RöFo - Fortschritte auf dem Gebiet der Röntgenstrahlen und der bildgebenden Verfahren* 196, no. 02 (February 2024): 134–53. https://doi.org/10.1055/a-2178-2846.

Vogel-Claussen, Jens, Florian Lasch, Benjamin-Alexander Bollmann, Katharina May, Alexander Kuhlmann, Gerald Schmid-Bindert, Rudolf Kaaks, Jörg Barkhausen, Sabine Bohnet, and Martin Reck. “Design and Rationale of the HANSE Study: A Holistic German Lung Cancer Screening Trial Using Low-Dose Computed Tomography.” *RöFo - Fortschritte auf dem Gebiet der Röntgenstrahlen und der bildgebenden Verfahren* 194, no. 12 (December 2022): 1333–45. https://doi.org/10.1055/a-1853-8291.

Vrachnis, Nikolaos, and Nikolaos Vlachadis. “Guidelines on Cervical and Breast Cancer Screening in Greece.” *The Lancet* 385, no. 9970 (February 28, 2015): 772. https://doi.org/10.1016/S0140-6736(15)60434-2.

Wait, Suzanne, Arturo Alvarez-Rosete, Tasnime Osama, Dani Bancroft, Robin Cornelissen, Ante Marušić, Pilar Garrido, et al. “Implementing Lung Cancer Screening in Europe: Taking a Systems Approach.” *JTO Clinical and Research Reports* 3, no. 5 (April 22, 2022): 100329. https://doi.org/10.1016/j.jtocrr.2022.100329.

webteam. “Ενημερωτικό σημείωμα για τη σύσκεψη υπό τον Πρωθυπουργό Κυριάκο Μητσοτάκη με αντικείμενο τις προληπτικές εξετάσεις και την πρωτοβάθμια φροντίδα | Ο Πρωθυπουργός της Ελληνικής Δημοκρατίας,” April 20, 2023. https://www.primeminister.gr/2023/04/20/31715.

Wickens, Charlotte. “Why Do Diagnostics Matter?,” n.d.

Wigzell, Olivia. “Nationella screeningprogram.” *Socialstyrelsen*, n.d. https://www.socialstyrelsen.se/globalassets/sharepoint-dokument/artikelkatalog/nationella-screeningprogram/2019-4-12.pdf.

“Workshop Screening Cancro Polmone Razionale.” Accessed October 15, 2024. https://www.alcase.eu/wp-content/uploads/2019/01/WS_screening-cancro-polmone-RAZIONALE_14-diic2018.pdf.

Wormanns, Dag, Hans-Ulrich Kauczor, Gerald Antoch, Jürgen Biederer, Felix J. F. Herth, Jens Vogel-Claussen, Niels Reinmuth, Michael Pfeifer, Board of Directors, Deutsche Röntgengesellschaft, and Board of Directors, Deutsche Gesellschaft für Pneumologie und Beatmungsmedizin. “Joint Statement of the German Radiological Society and the German Respiratory Society on a Quality-Assured Early Detection Program for Lung Cancer with Low-Dose CT.” *RöFo - Fortschritte auf dem Gebiet der Röntgenstrahlen und der bildgebenden Verfahren* 191, no. 11 (November 2019): 993–97. https://doi.org/10.1055/a-0998-4399.

Το site για την καλή Υγεία, την σωστή Διατροφή και την Ευεξία - ygeiamou.gr. “Metropolitan Hospital: Το Πρόγραμμα «Πνευμόνων Υγεία» Που Σώζει Ζωές,” December 1, 2022. https://www.ygeiamou.gr/επιστημονικές-εξελίξεις/309190/metropolitan-hospital-to-programma-pnevmonon-igia-pou-sozi-zoes/.

Το site για την καλή Υγεία, την σωστή Διατροφή και την Ευεξία - ygeiamou.gr. “Καρκίνος Πνεύμονα: Η Κοινωνική Ανισότητα Εμπόδιο Στην Αντιμετώπιση Των Ασθενών,” November 28, 2023. https://www.ygeiamou.gr/ειδήσεις/361984/karkinos-pnevmona-i-kinoniki-anisotita-empodio-stin-antimetopisi-ton-asthenon/.

Υπουργείο Υγείας. “Εθνικό Σχέδιο Δράσης για την πρόληψη και την αντιμετώπιση του καρκίνου,” n.d. https://www.moh.gov.gr/articles/ministry/grafeio-typoy/press-releases/11810-ethniko-sxedio-drashs-gia-thn-prolhpsh-kai-thn-antimetwpish-toy-karkinoy.

Υπουργείο Υγείας. “Εθνικό Σχέδιο Δράσης για την πρόληψη και την αντιμετώπιση του καρκίνου.” Accessed October 16, 2024. https://www.moh.gov.gr/articles/ministry/grafeio-typoy/press-releases/11810-ethniko-sxedio-drashs-gia-thn-prolhpsh-kai-thn-antimetwpish-toy-karkinoy.
